# Marine organisms as potential sources of natural products for the prevention and treatment of malaria

**DOI:** 10.1039/d2ra07977a

**Published:** 2023-02-02

**Authors:** Walaa A. Negm, Shahira M. Ezzat, Ahmed Zayed

**Affiliations:** a Department of Pharmacognosy, Tanta University, College of Pharmacy El-Guish Street Tanta 31527 Egypt walaa.negm@pharm.tanta.edu.eg ahmed.zayed1@pharm.tanta.edu.eg; b Department of Pharmacognosy, Faculty of Pharmacy, Cairo University Kasr El-Aini Street Cairo 11562 Egypt shahira.ezzat@pharma.cu.edu.eg; c Department of Pharmacognosy, Faculty of Pharmacy, October University for Modern Sciences and Arts (MSA) Giza 12451 Egypt

## Abstract

Vector-borne diseases (VBDs) are a worldwide critical concern accounting for 17% of the estimated global burden of all infectious diseases in 2020. Despite the various medicines available for the management, the deadliest VBD malaria, caused by *Plasmodium* sp., has resulted in hundreds of thousands of deaths in sub-Saharan Africa only. This finding may be explained by the progressive loss of antimalarial medication efficacy, inherent toxicity, the rise of drug resistance, or a lack of treatment adherence. As a result, new drug discoveries from uncommon sources are desperately needed, especially against multi-drug resistant strains. Marine organisms have been investigated, including sponges, soft corals, algae, and cyanobacteria. They have been shown to produce many bioactive compounds that potentially affect the causative organism at different stages of its life cycle, including the chloroquine (CQ)-resistant strains of *P. falciparum*. These compounds also showed diverse chemical structures belonging to various phytochemical classes, including alkaloids, terpenoids, polyketides, macrolides, and others. The current article presents a comprehensive review of marine-derived natural products with antimalarial activity as potential candidates for targeting different stages and species of *Plasmodium* in both *in vitro* and *in vivo* and in comparison with the commercially available and terrestrial plant-derived products, *i.e.*, quinine and artemisinin.

## Abstract 2

Vector-borne diseases (VBDs) are a worldwide critical concern accounting for 17% of the estimated global burden of all infectious diseases in 2020.

## Graphical abstract

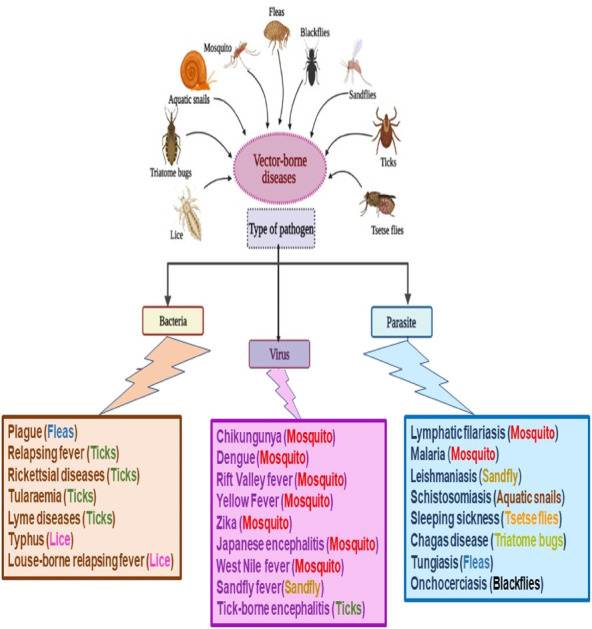

## 1. Introduction

**Fig. 1:**
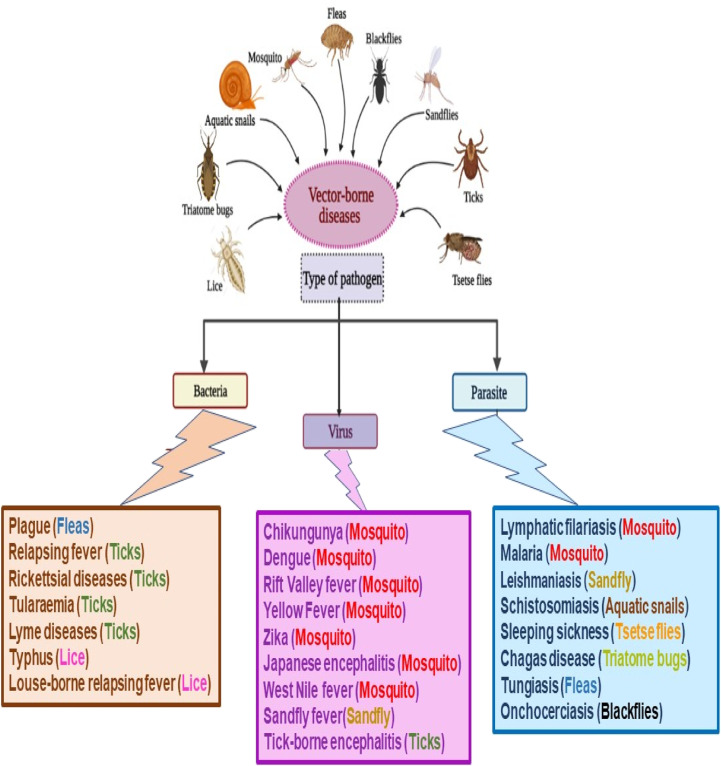
Classification of vector-borne diseases (VBDs) according to pathogen types.

Vector-borne diseases (VBDs) are infectious diseases caused by parasites, bacteria, and viruses transmitted *via* vectors. About 700 000 deaths are reported officially by the World Health Organization (WHO) from these diseases per year worldwide, including malaria, dengue, schistosomiasis, human African trypanosomiasis, leishmaniasis, Chagas disease, chikungunya fever, Zika virus fever, yellow fever, West Nile fever, Japanese encephalitis, and onchocerciasis, Fig. 1. Based on the WHO reports released in 2020, VBDs are a worldwide concern that accounts for 17% of the estimated global burden of all infectious diseases.^1^

VBDs are commonly associated with weather and climate, where the incidence of these diseases is mainly in the tropics and subtropical regions. The low hygiene, sanitation, waste management, and housing in these urban areas help also spread such diseases between the world's poorest people, communities, and countries.^2^ Various native to these regions as arthropods, including mosquitoes, ticks, sand flies, triatomine bugs, cockroaches, lice, fleas, and aquatic snails, are involved as mediators transmitting VBDs.^3,4^

Particularly, malaria is the most challenging VBD that leads to health problems worldwide, especially in developing countries. It is a mosquito-borne infectious disease that affects humans and other animals. An estimated 405 000 malaria deaths worldwide were registered, along with 228 million cases in 2018, compared to 229 million cases and 409 000 deaths with more than 400 000 deaths in 2019, based on the WHO report.^5,6^ In other words, malaria accounts for more than 50% of VBDs deaths.

**Fig. 2:**
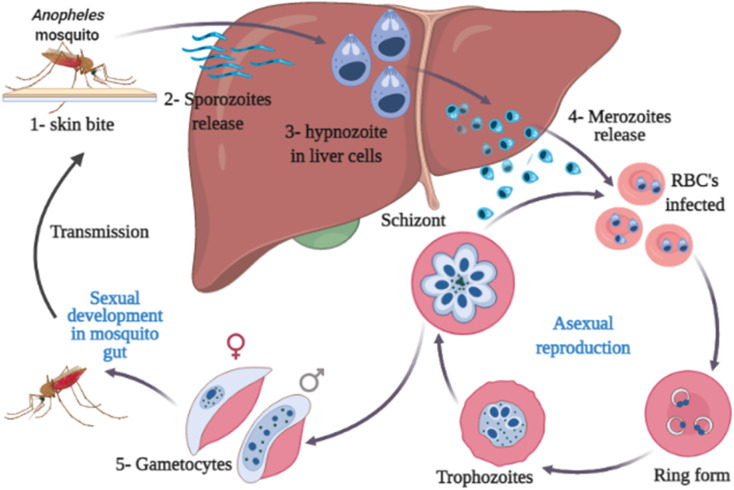
Life cycle of the malaria parasite.

Malaria is transmitted through the bite of an infected Anopheles female mosquito. The infected mosquitoes carry one of several protozoans belonging to the genus *Plasmodium* (*P*. *falciparum*, *P*. *ovale*, *P*. *vivax*, *P. knowlesi*, and *P*. *malariae*).^7,8^ The parasite is then released into the bloodstream causing severe anemia and other signs and symptoms, including chills, fever, profuse sweating, headache, nausea, vomiting, abdominal pain, diarrhea, muscle pain, convulsions, coma, bloody stools.^6,9,10^ Serious complications or even death can occur in case of improper diagnosis or treatment.^11–13^ The life cycle of the malaria parasite of *Plasmodium* sp. is illustrated in Fig. 2. This figure is of great importance for helping drug discovery processes of novel drugs targeting critical stages in the parasite life cycle.

**Fig. 3:**
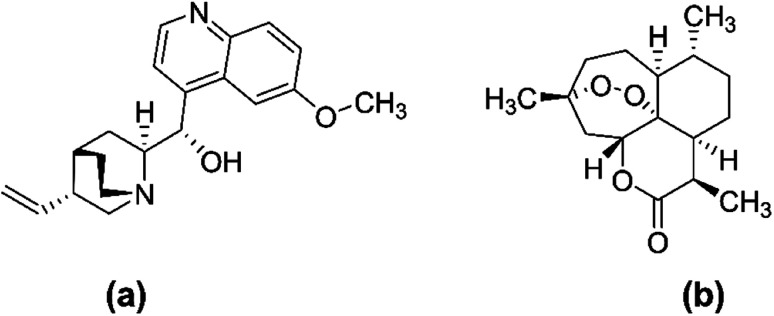
Chemical structure of quinine (a) and artemisinin (b).

Chloroquine (CQ) and hydroxychloroquine are two existing chemical medicines that have limited usefulness and efficacy as antimalarials due to their high cost, unpleasant side effects, and evolution of multi-drug resistance associated with them.^14^ Hence, there has been an urgent need and continuous search for novel sources of more efficacious drugs to combat the disease. Nevertheless, natural products, including terrestrial medicinal plants, have a long history in the treatment of malaria owing to their relative efficacy, safety, reasonable cost, and availability.^15,16^ The two most successful antimalarial drugs; namely artemisinin and quinine (Fig. 3), were sourced from medicinal plants of cinchona qinghao (*Artemisia annua*, Family Asteraceae) and (*Cinchona officinalis*, Family Rubiaceae), respectively, and have been used for hundreds of years and before the mosquito cycle was explored. Even today, in the fight against malaria, both quinine and artemisinin are still of prime importance.^17^

In contrast to terrestrial plants, marine organisms do not have a remarkable history of use in traditional medicine. However, recent advances in marine biology and engineering have helped investigation and scientific exploration of the marine environment to identify and isolate novel compounds, which have proven their potential bioactivities against life-threatening diseases, including tumor and viral infections.^18–20^ More than 30 000 compounds have been identified from about 240 000 known species of marine organisms.^21^ Few of them have been approved by the Food and Drug Administration (FDA), including ziconotide (Prialt®) as a potent analgesic, trabectedin (Yondelis®), and cytarabine or ara-C (Cytosar-U®) as anti-tumor agents, vidarabine or ara-A (Vira-A^®^) and iota-carrageenan (Carragelose^®^) as an antiviral, and omega-3-acid ethyl ester (Lovaza^®^) for treating hypertriglyceridemia (Table 1).^22–24^

**Table 1:** A list of some examples of approved marine-derived drugs currently on the market

Trade name	Scientific name	Source	Family	Indication	Ref.
Prialt^®^	Ziconotide	Cone snail species *Conus magus*	Conidae	potent analgesic	22–24
Yondelis^®^	Trabectedin	*Candidatus Endoecteinascidia frumentensis*	Unclassified family candidatus endolissoclinum	Anti-tumor	22–24
Cytosar-U^®^	Cytarabine or ara-C	*Cryptotethia crypta* sponge	Tethyidae	Anti-tumor	22–24
Carragelose^®^	Iota-carrageenan	*Eucheuma denticulatum* sponge	Solieriaceae	Antiviral	22–24
Vira-A^®^	Vidarabine or ara-A	*Tectitethya crypta* sponges	Tethyidae	Antiviral	22–24

Recently, Nweze, *et al.* published a review article highlighting the potential of marine-derived natural products for the treatment of some examples of diseases for neglected communities, including malaria, leishmaniasis, and trypanosomiasis.^25^ Although some of the previous studies could not identify the chemical structure of bioactive components that acted significantly against malaria,^26^ the current article focuses on malaria. It reviews the different chemical classes, *i.e.*, alkaloids, terpenoids, endoperoxides, phosphotriesters, peptides and depsipeptides, and macrolides, derived from marine organisms, including sponges, cyanobacteria, actinomycete bacteria, soft corals, and algae. These bioactive have been confirmed to be potential candidates for managing malaria compared to commercially available products by targeting various stages in *Plasmodium* sp. life cycle. Moreover, the half maximum cytotoxic (CC_50_) and inhibitory concentration (IC_50_) against the different stages of the malaria parasite shall be highlighted, in addition to the possible mechanism of action and structure-activity relationships (SAR) in previous reports investigated the antiplasmodium activity. Hence, the current review may open new frontiers for discovery and approval of novel potent drugs for this life-threatening disease.

## 2. Alkaloids

Various classes of marine-derived alkaloids have shown potent antimalarial activity. β-Carboline, indole, imidazole, and pyrrole alkaloids are mostly found. They showed bioactivities against different stages of the *Plasmodium* parasite with a unique mechanism of action. Fifteen classes represented by 67 compounds were reviewed. Among them are manzamine alkaloids which showed inhibitory activity against glycogen synthase 3 (GSK-3) topoisomerase. In addition, salinosporamide showed a potent protease inhibitory effect. Numerous marine-derived alkaloids shall be discussed in detail in the following sub-sections and Table 2, including their sources, IC_50_, chemical structures, SAR, and mechanism of action.

**Table 2:** A list of marine-derived antimalarial alkaloids showing their IC_50_ against various strains of *Plasmodium* sp., chemical structure and biogenic source^a^

Compound	Antiplasmodial activity (IC_50_ value)	Structure	Source	Marine class	Ref.
Manzamine A (1)	W2 = 0.015 μM D6 = 0.0082 μM	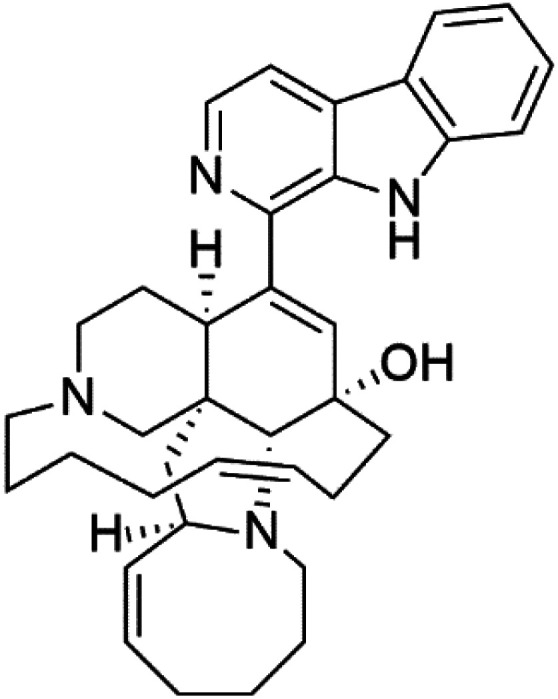	Okinawan *Haliclona*	Sponge	27
8-Hydroxymanzamine (2)	W2 = 0.014 μM D6 = 0.010 μM	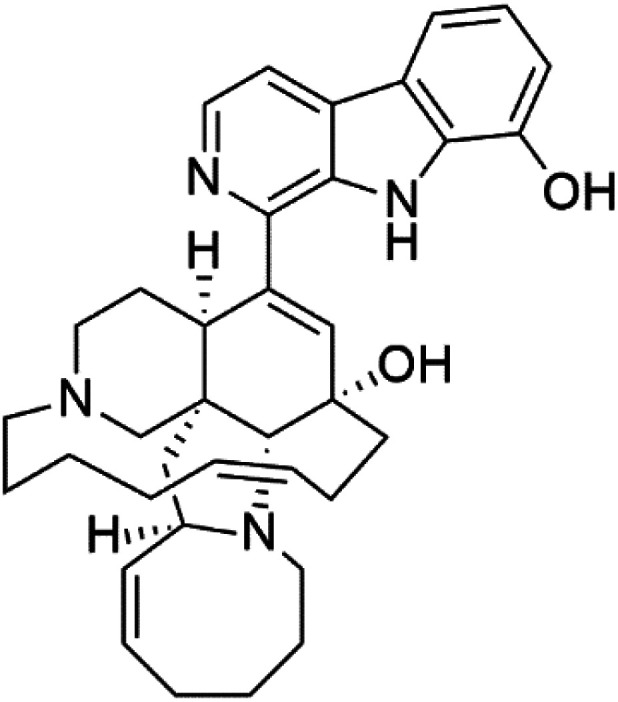		Sponge	27
Manzamine F (3)	W2 = 2.93 μM 6 = 1.34 μM	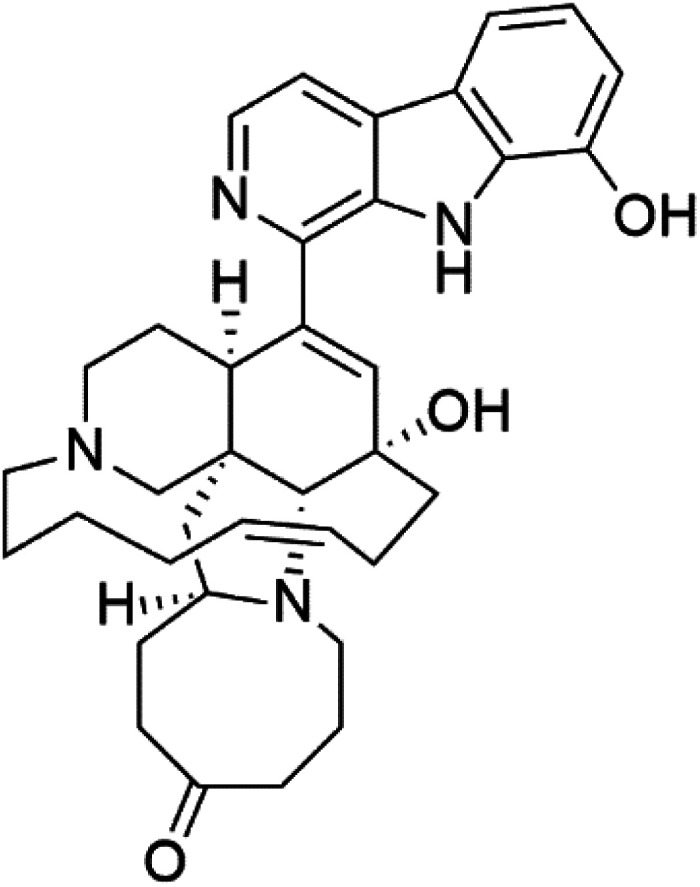		Sponge	27
6-Hydroxymanzamine (4)	W2 = 1.5 μM D6 = 1.36 μM	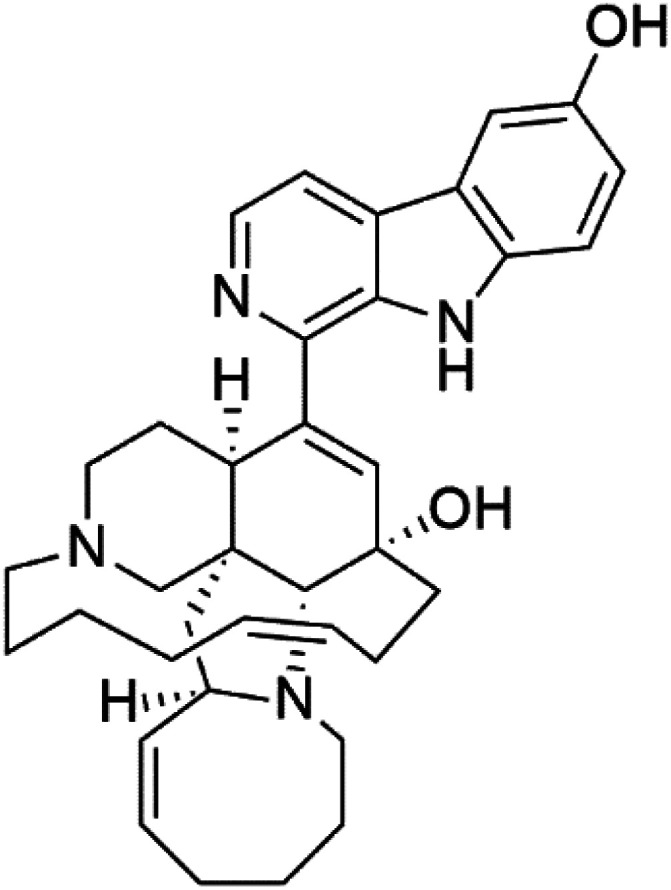		Sponge	27
Neo-kauluamine (5)	D6 = 1.46 μM W2 = 2.41 μM	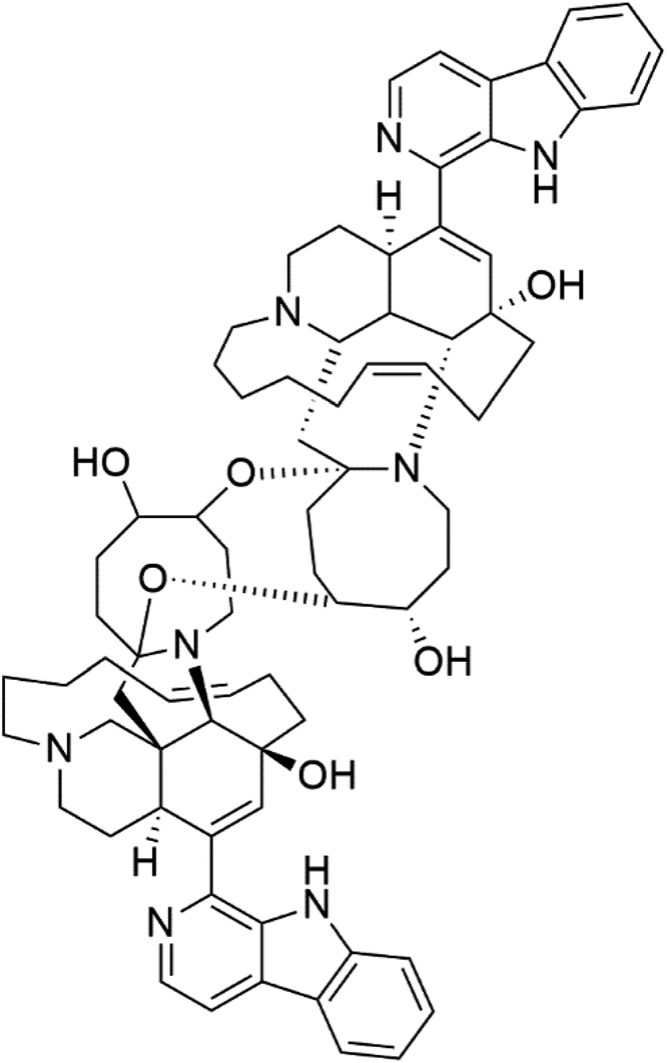	Indo-pacific sponge	Sponge	28
12,34-Oxamanzamine A (6)	D6 = 8.97 μM	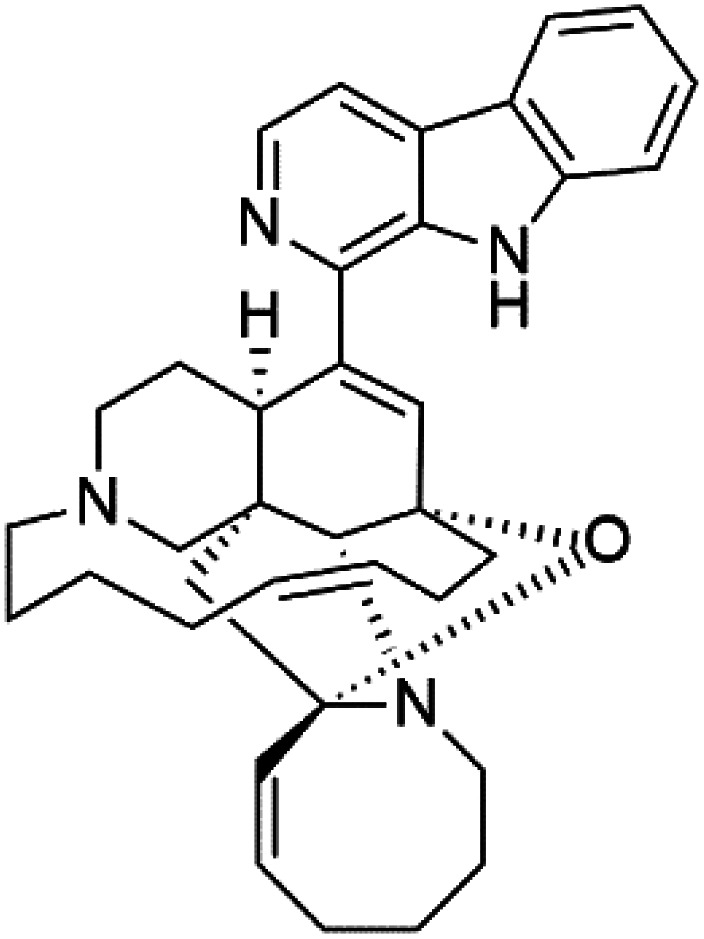		Sponge	28
Zamamidine A (7)	0.0008 to 0.016 μM	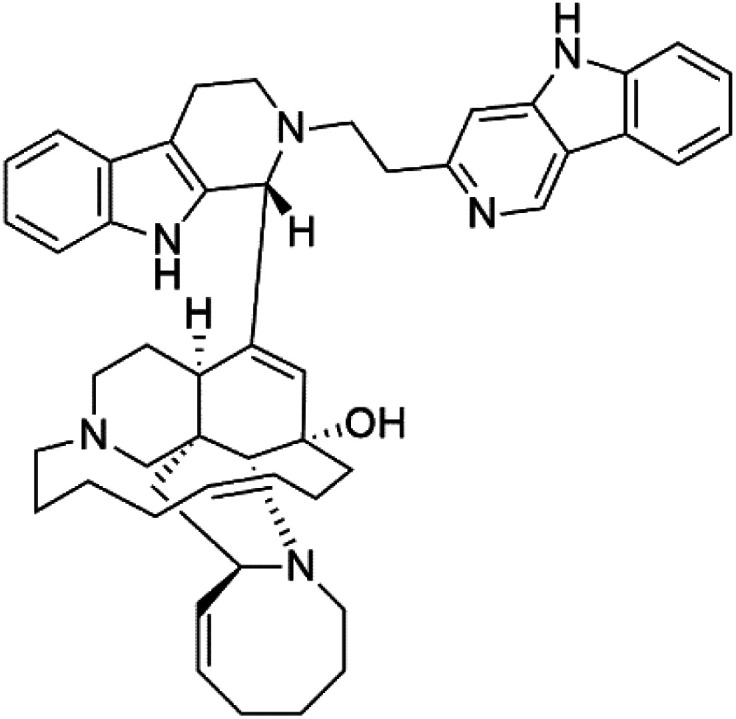	*Amphimedon* sp.	Sponge	35
Zamamidine B (8)		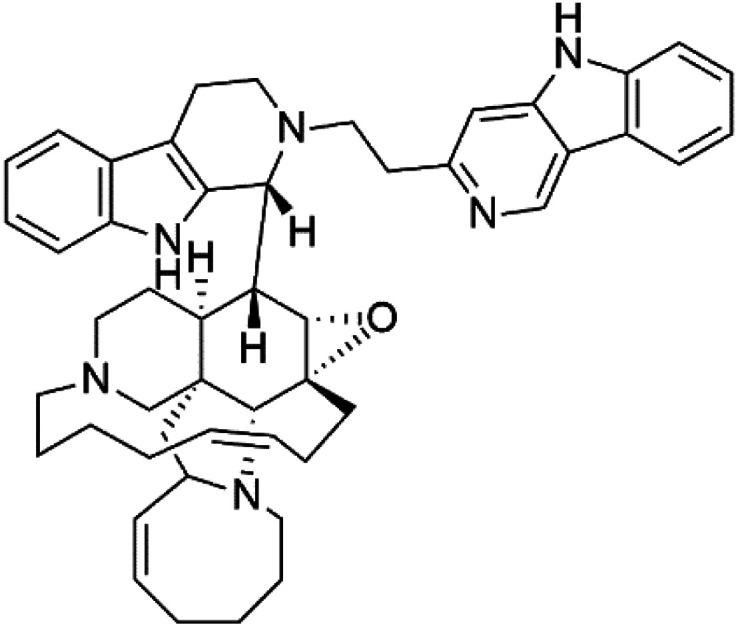		Sponge	
Zamamidine C (9)		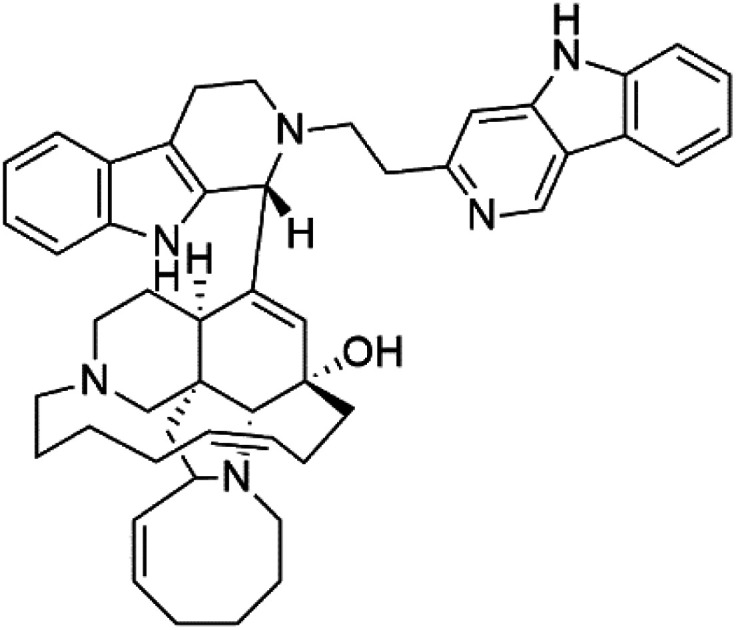		Sponge	
Zamamidine D (10)		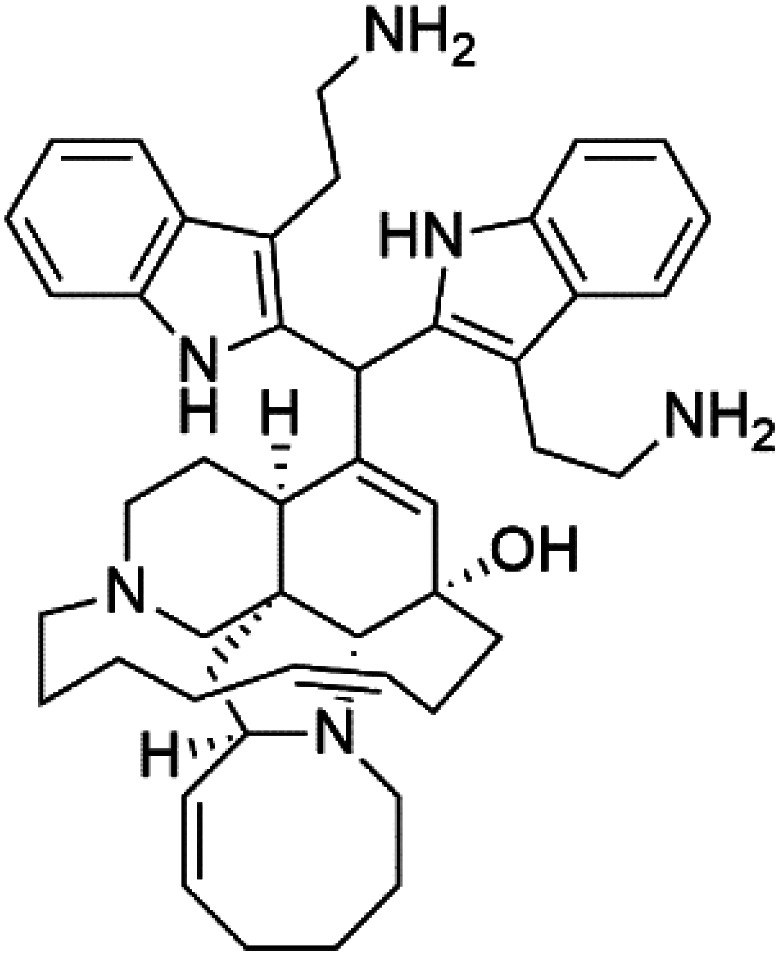		Sponge	
Homofascaplysin (11)	K1 = 0.04 μM NF54 = 0.07 μM	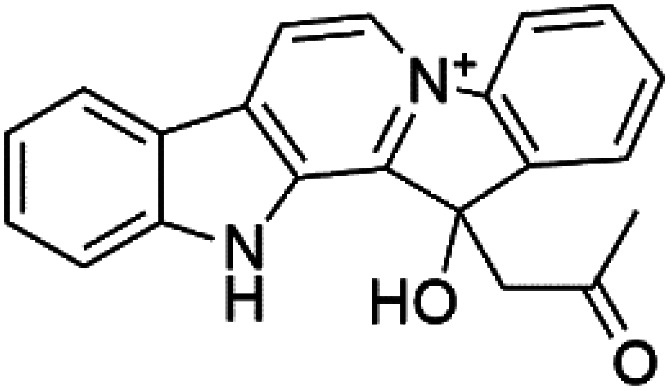	*Hyrtios* sponge	Sponge	38
Marinacarboline A (12) Marinacarboline B (13) Marinacarboline C (14)	3D7 and Dd2 IC_50_ from 1.92 to 36.03 μM	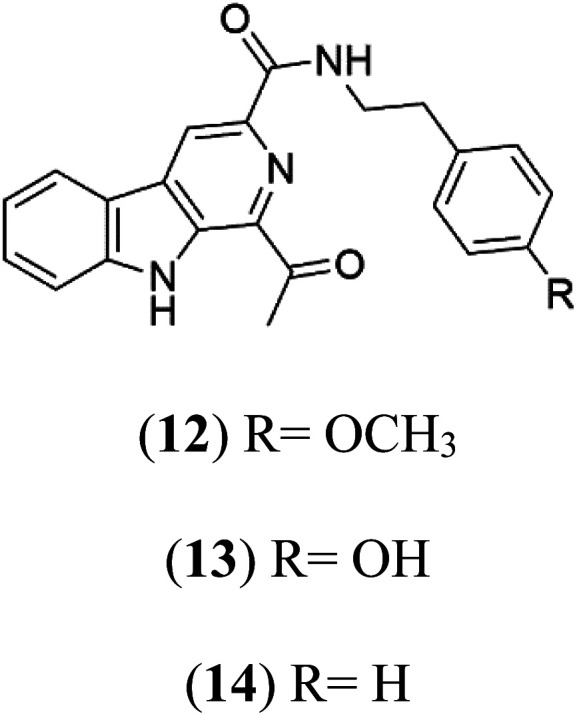	*Marinactinospora thermotolerans*	*Actinomycete bacteria*	39
Marinacarboline D (15)		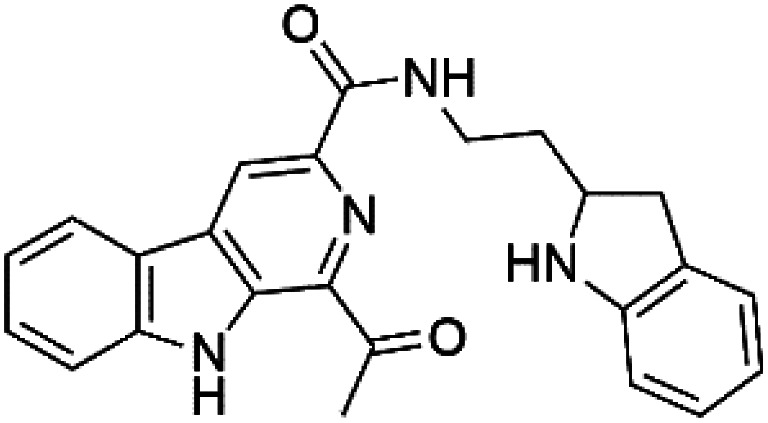			
13-*N*-Demethyl-methylpendolmycin (16) methylpendolmycin-14-*O*-α-glucoside (17)	3D7 = 20.75 and 10.43 μM Dd2 = 18.67 and 5.03 μM	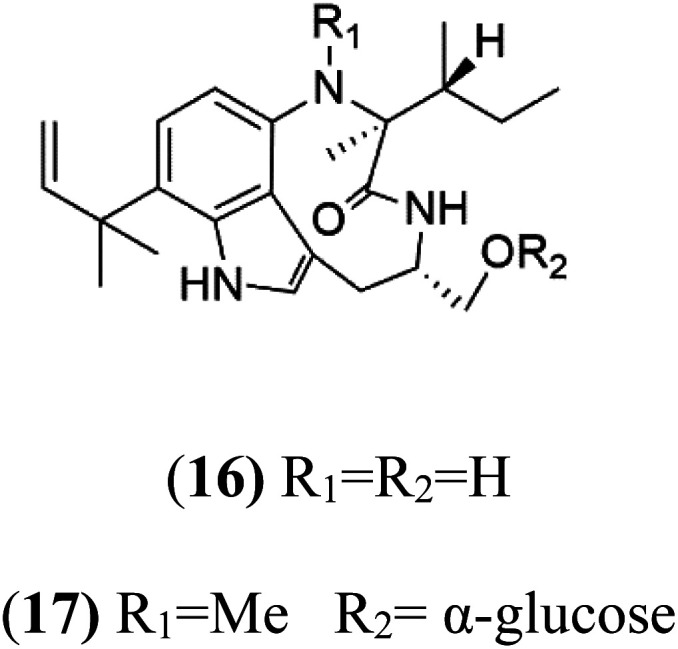	*Marinactinospora thermotolerans*	*Actinomycete bacteria*	38
Crambescidin 800 (18)	3D7 = 0.16 μM FCR3 = 0.24 μM	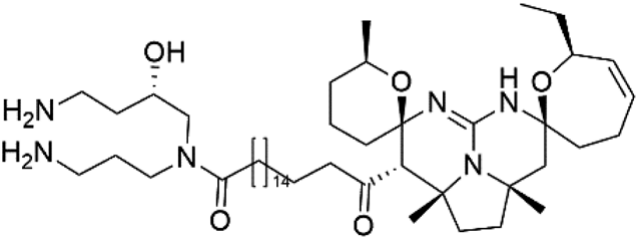	*Monanchora unguiculate*	Sponge	39 and 40
Crambescidin 359 (19) crambescidin acid (20)	3D7 = 20.75 and 10.43 μM Dd2 = 18.67 and 5.03 μM	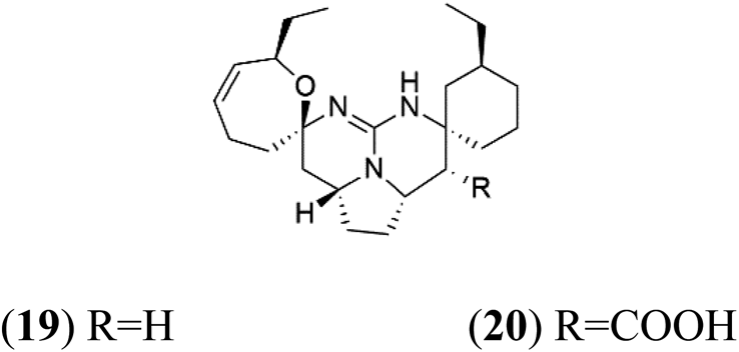			
Fromiamycalin (21)	3D7 = 0.24 μM	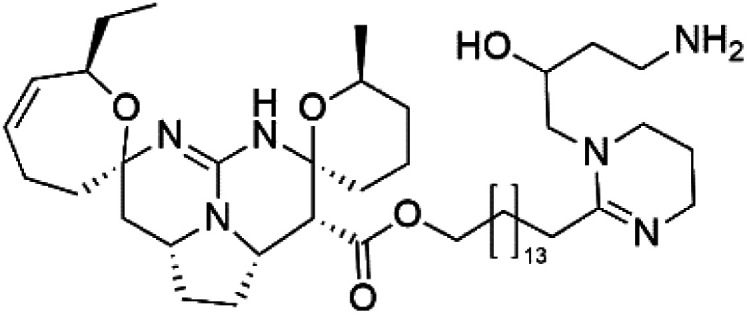			
Unguiculin A (22)	3D7 = 12.86 μM	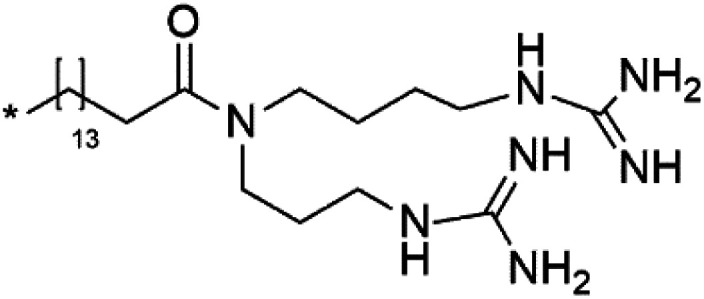			
Ptilomycalins E (23) Ptilomycalins F (24) Ptilomycalins G (25)	3D7 = 0.35, 0.23, and 0.46 μM	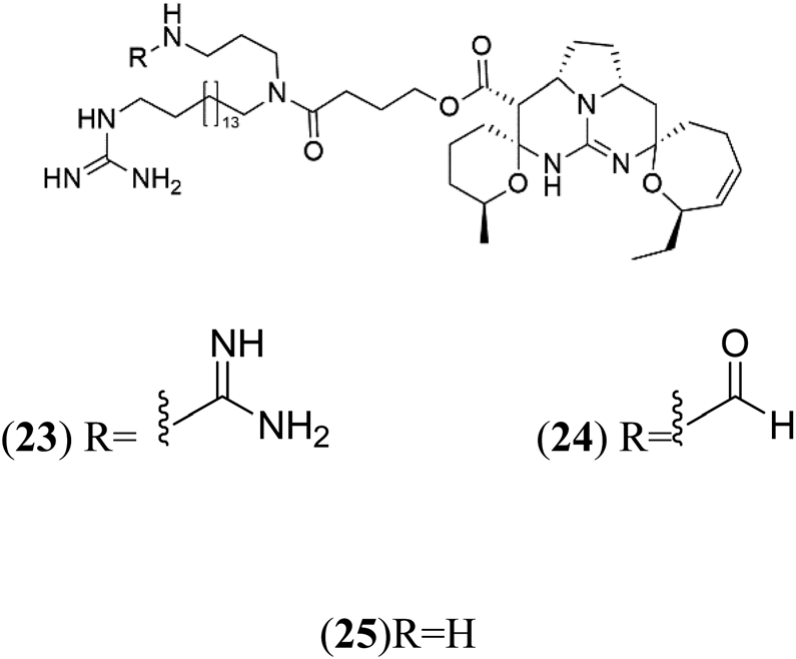			41
Ptilomycalins H (26)	3D7 = 0.46 μM	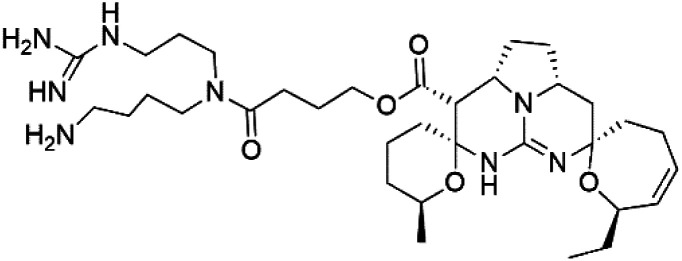			
Opacaline B (27) Opacaline C (28)	*K*1 range of 2.5–14 μM	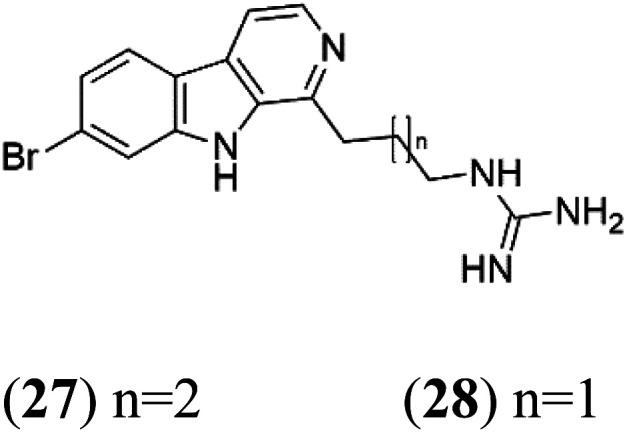	*Pseudodistoma opacum*	New Zealand ascidian	42
Didemnidine A (29) Didemnidine B (30)	K1 = 0.047 μM	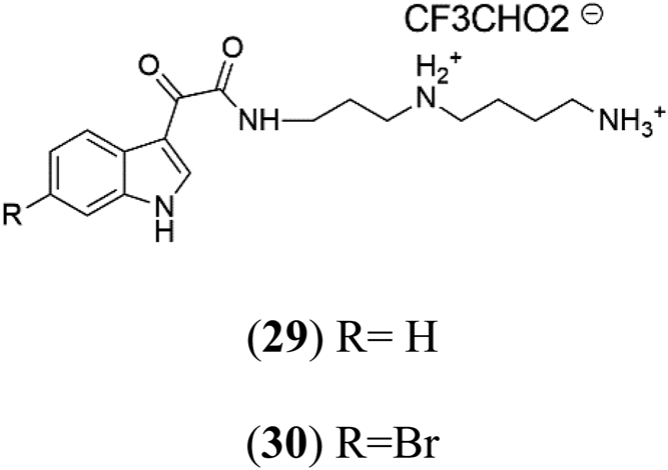	Ascidian *Didemnum* sp.	Marine tunicate	43
Salinosporamide A (31)	3D7 = 11.4 nM FCB = 19.6 nM	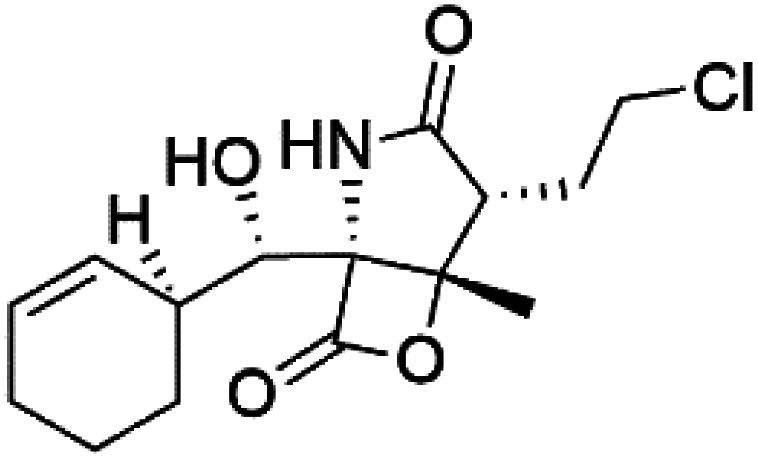	Salinispora tropica	Marine actinomycete bacteria	44
Psammaplysin H (32) Psammaplysin F (33) Psammaplysin G (34)	3D7 = 0.41, 1.92, and 5.22 μM	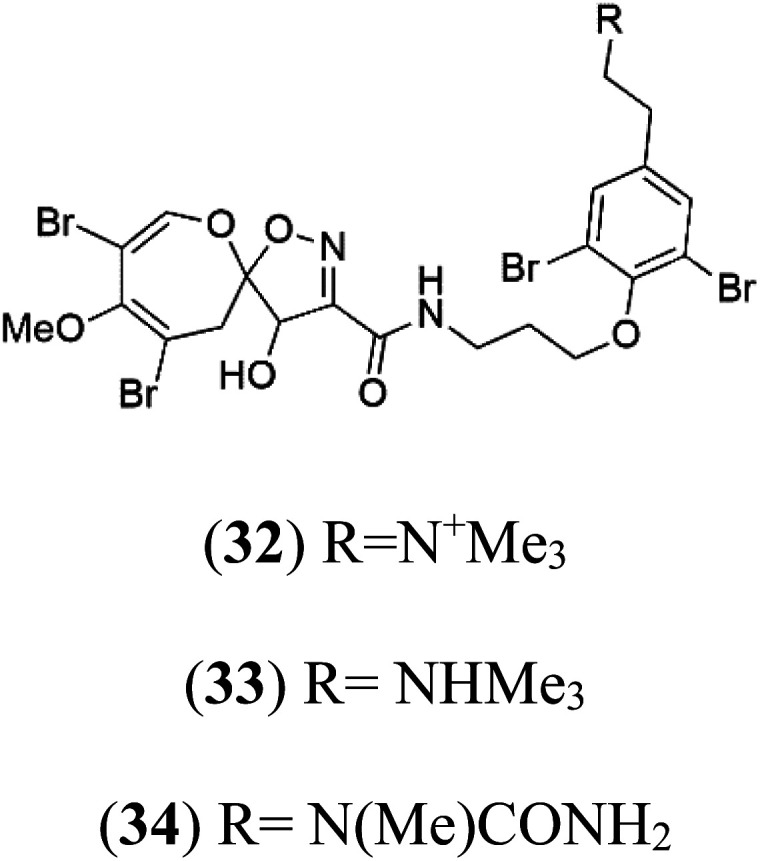	*Aplysinella strongylata*	Sponge	52
Ceratinadin E (35)	K1 = 0.9 μM	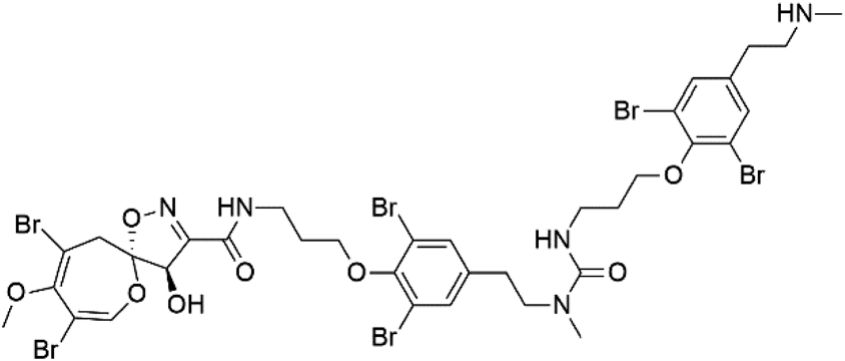	Okinawan Pseudoceratina	Sponge	54
Ceratinadin F (36)	K1 > 8.16 μM	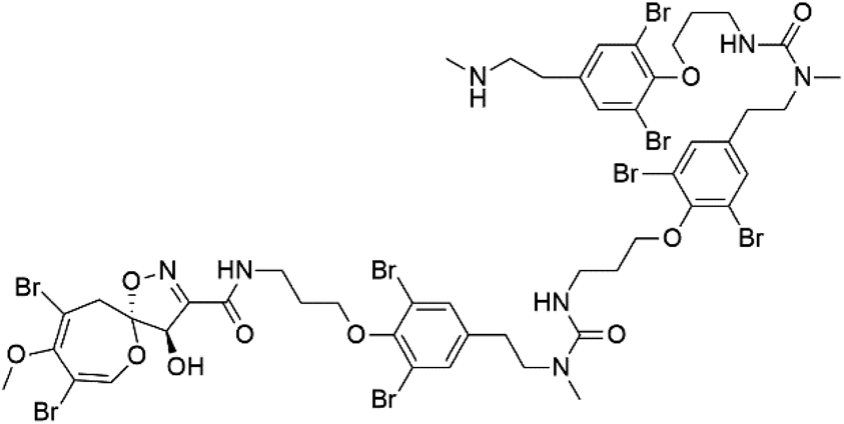			
Tsitsikammamine C (37)	3D7 = 13 nM Dd2 = 18 nM	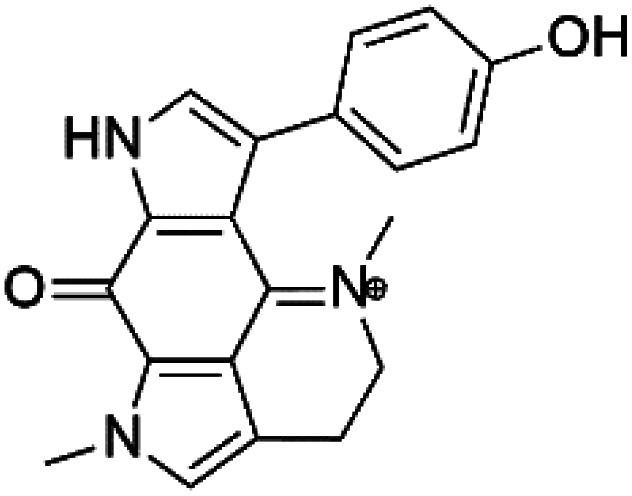	*Zyzzya* sp.	Sponge	55
Makaluvamine J (38)	3D7 = 25 nM Dd2 = 22 nM	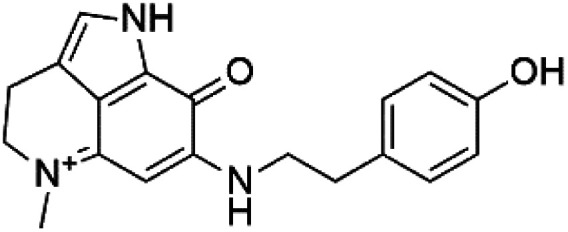			55
Makaluvamine G (39) Makaluvamine L (40)	3D7 = 36, 40 nM Dd2 = 39, 21 nM	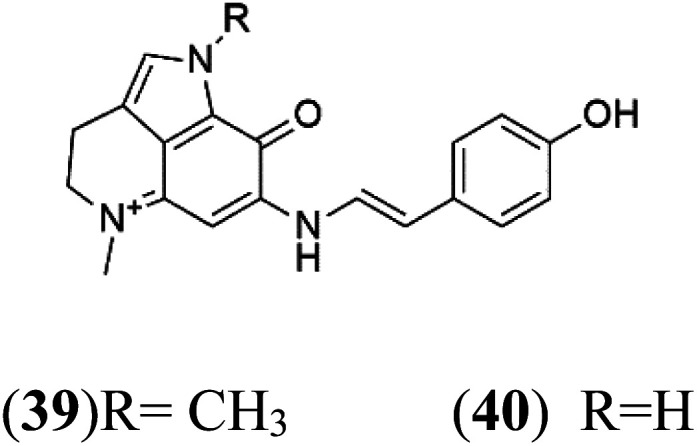			
Makaluvamine K (41)	3D7 = 396 nM Dd2 = 300 nM	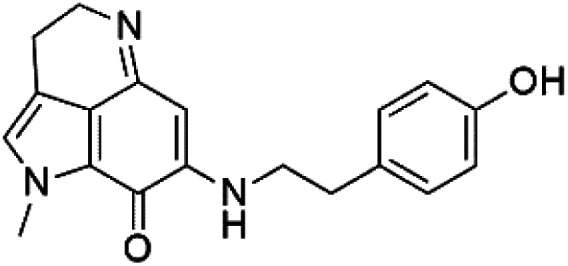			
Damirone A (42) Damirone B (43)	3D7 = 1880 Dd2 = 360 nM	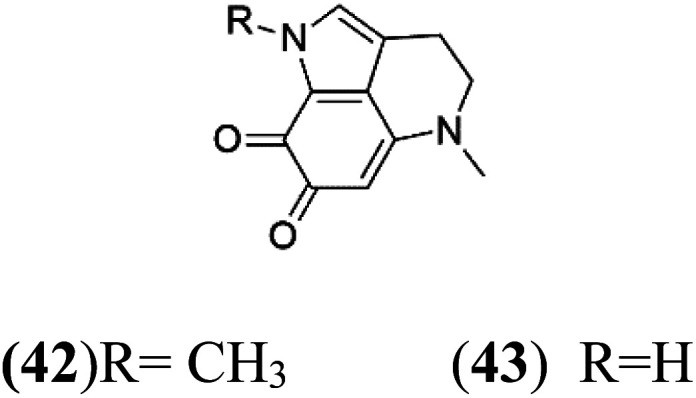			
Dihydrodiscorhabdin B (44)	D6 = 0.17 μM W2 = 0.13 μM	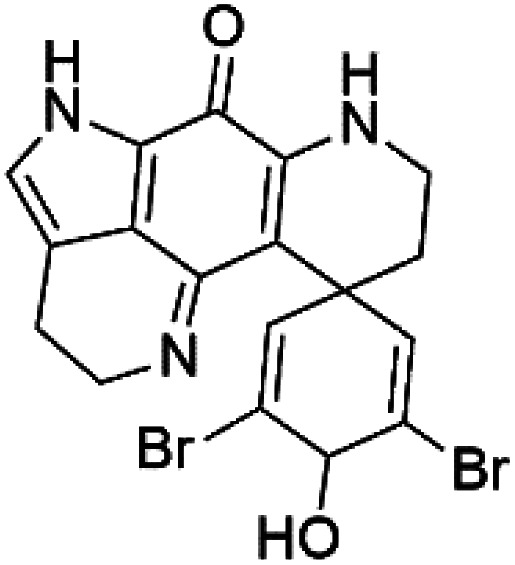	*Latrunculia* sp.	Sponge	56
Discorhabdin Y (45)	EC_50_ = 0.5 μM	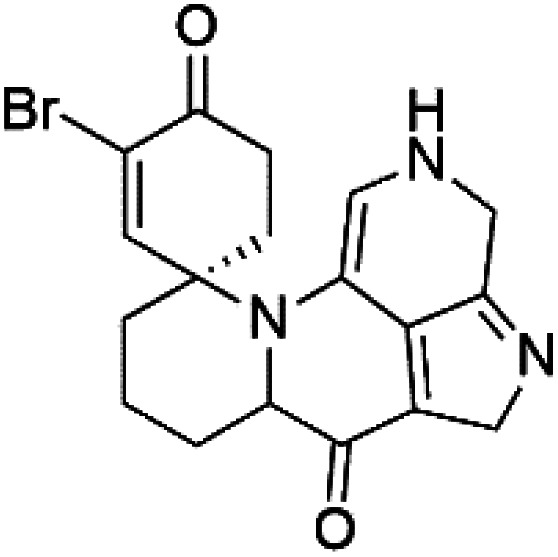			
Discorhabdin A (46)	D6 = 0.05 μM W2 = 0.05 μM	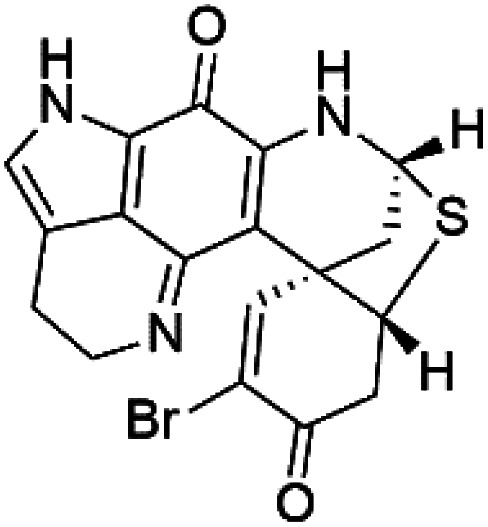			56
Discorhabdin C (47)	D6 = 2.8 μM W2 = 2.0 μM	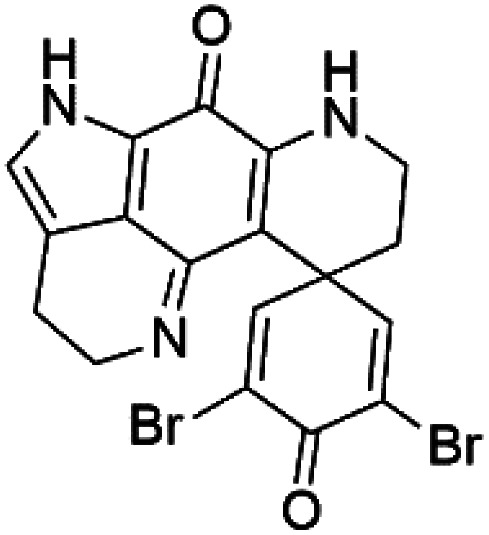			
Discorhabdin E (48)	W2 = 0.2 μM	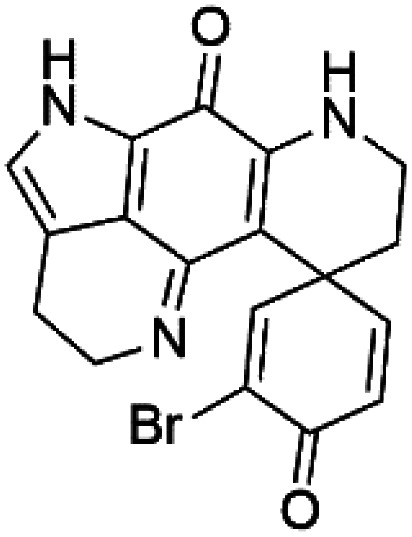			
Discorhabdin L (49)	W2 = 0.13 μM	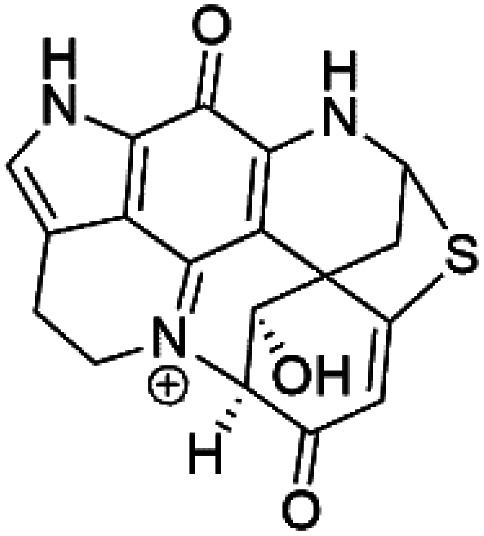			
Dihydrodiscorhabdin C (50)	D6 = 0.17 μM W2 = 0.13 μM	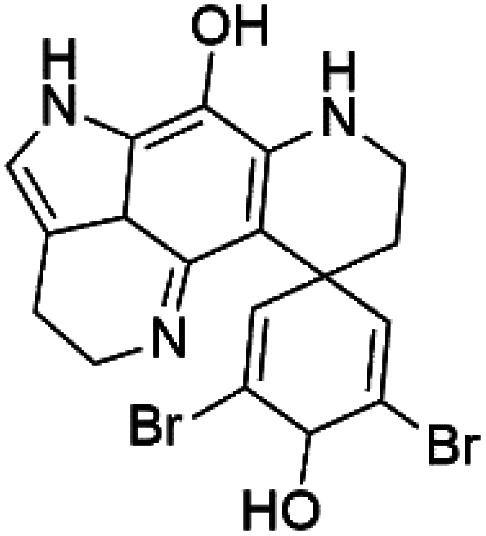			
Girolline (51)	FCM29 = 0.13 μM	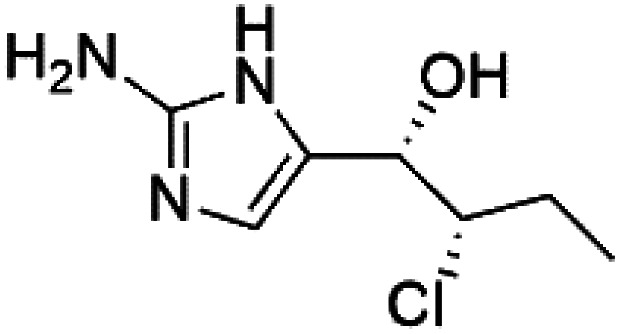	*Cymbastela cantharella*	Sponge	58
Thiaplakortone A (52)	3D7 = 51 Dd2 = 6.6 nM	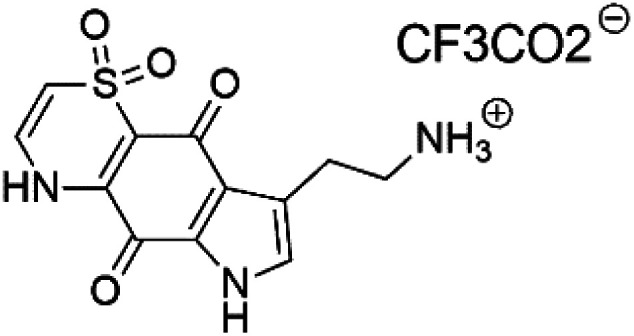	*Plakortis lita*	Sponge	59
Thiaplakortone B (53)	3D7 = 650 Dd2 = 92 nM	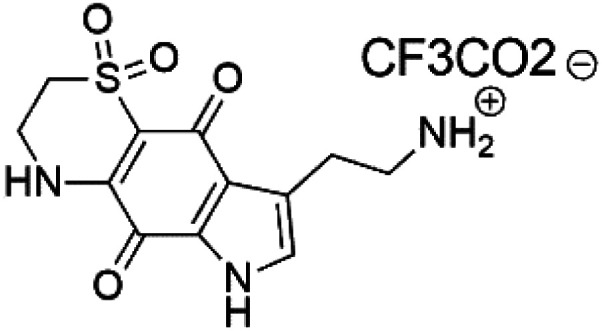			
Thiaplakortone C (54)	3D7 = 309 Dd2 = 171 nM	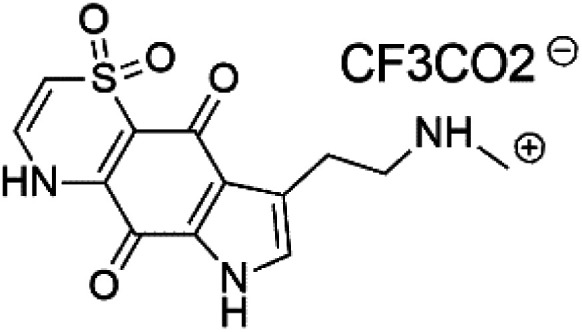			
Thiaplakortone D (55)	3D7 = 279 Dd2 = 159 nM	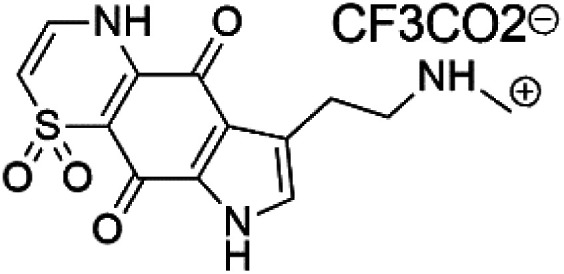			
Monamphilectine A (56)	W2 = 600 nM	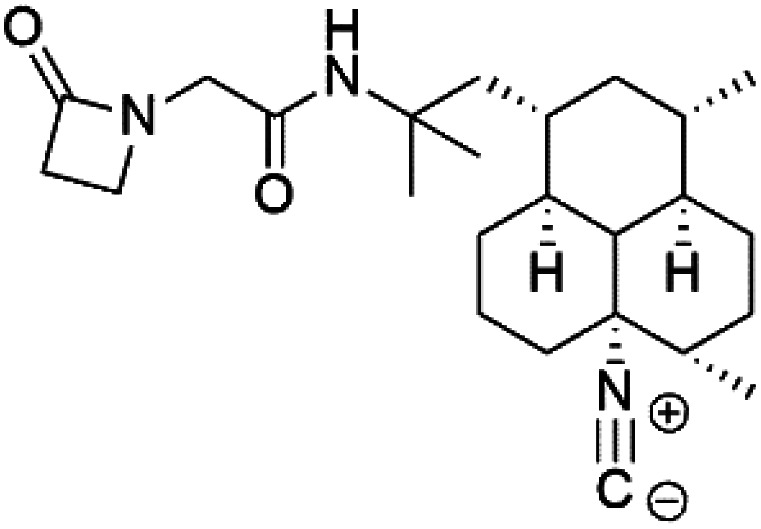	*Hymeniacidon* sp.	Sponge	60
Agelasine J (57)	FcB1 = 6.6 μM	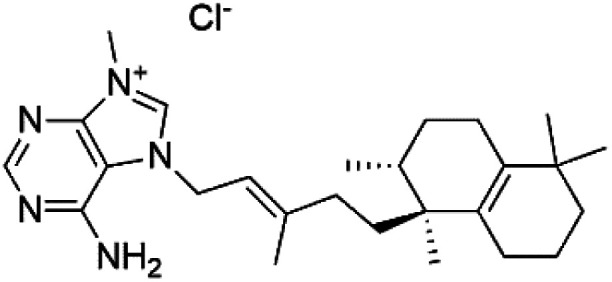	*Agelas mauritiana*	Sponge	61
Agelasine K (58)	FcB1 = 8.3 μM	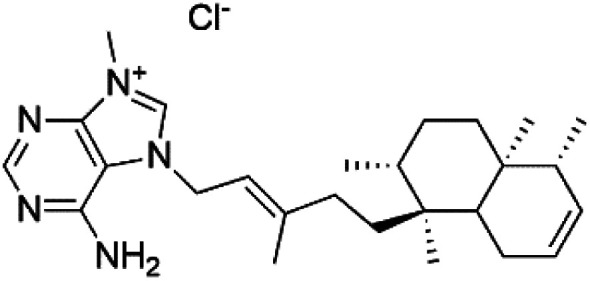			
Agelasine L (59)	FcB1 = 18 μM	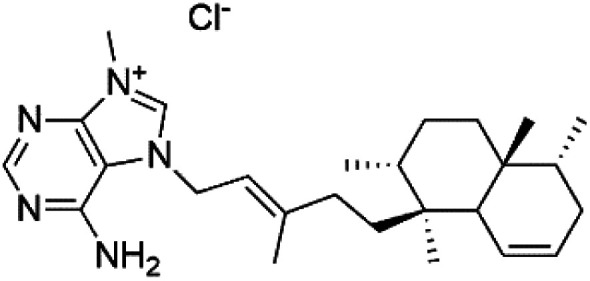			
Netamine G (60)	NA	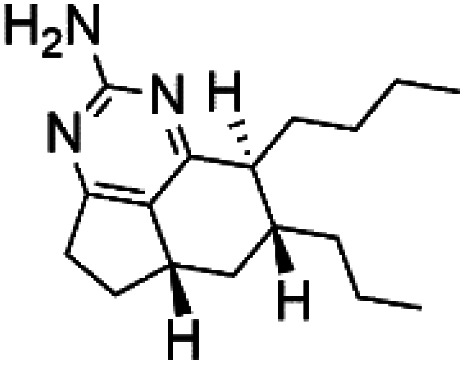	*Biemna laboutei*	Sponge	62
Netamine H (61)		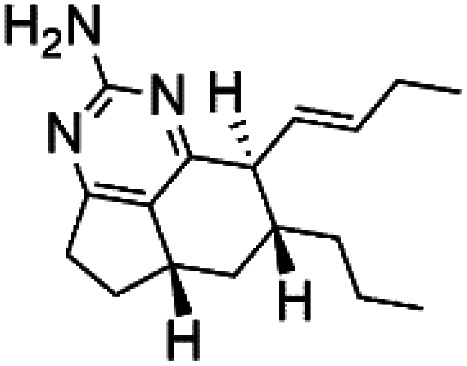			
Netamine I (62)		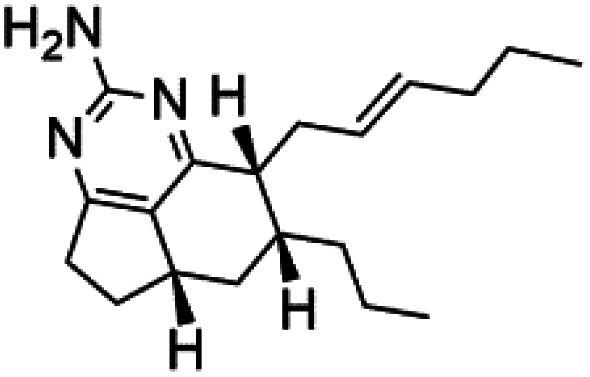			
Netamine J (63)		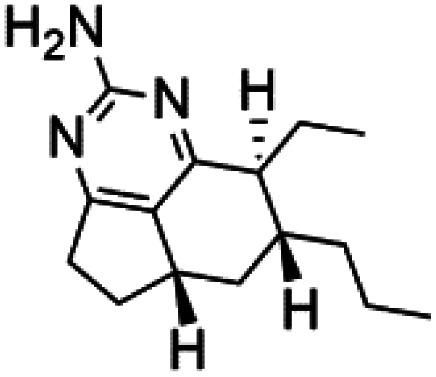			
Netamine K (64)	(64) IC_50_ = 2.4 μM NA for the other compounds (65–67)	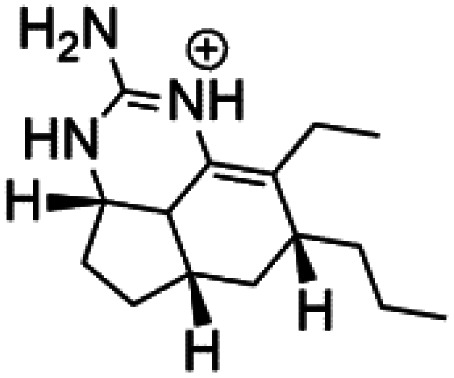			
Netamine L (65)		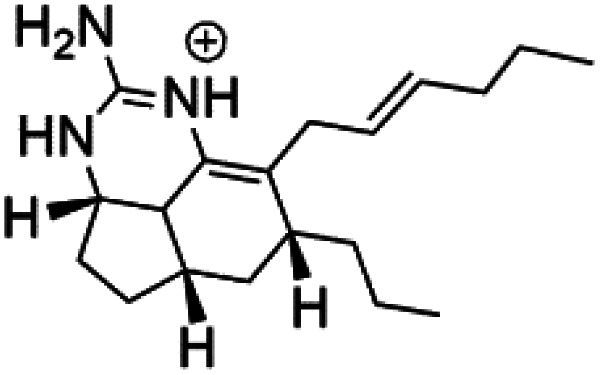			
Netamine M (66)		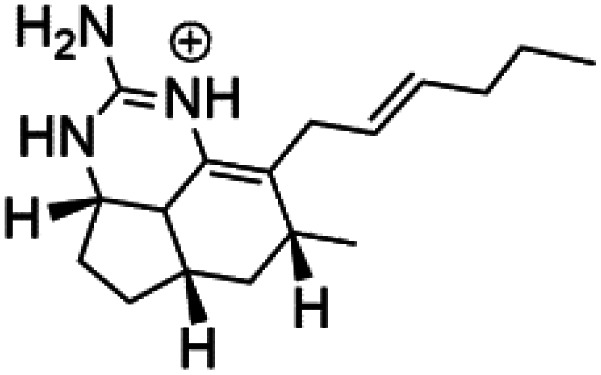			
Netamine N (67)		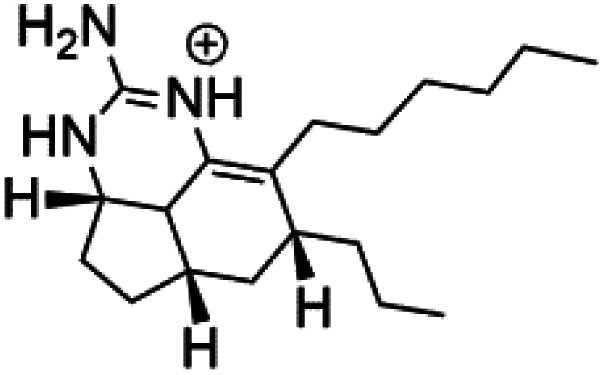	*Biemna laboutei*	Sponge	

a NA:not available.

### 2.1. Manzamines

Manzamines are polycyclic (7–8 rings) alkaloids containing a β-carboline moiety. Manzamine A (1) was first reported from an Okinawan sponge belonging to the *Haliclona* species (family Chalinidae).^27^ They are one of the essential antimalarial alkaloids. In addition to the lack of *in vivo* toxicity, the manzamines demonstrated greater effectiveness as antimalarial agents than the commonly used drugs artemisinin and CQ.^28^ The mechanism of manzamine alkaloids is not completely understood. Still, authors described β-carboline alkaloids as micromolar inhibitors of glycogen GSK-3 by malaria parasites and inhibitors of topoisomerase DNA through intercalation in DNA-base pairs.

Hence, a complete SAR investigation of manzamine alkaloids is necessary to understand the importance of each moiety (β-carboline and pentacyclic ring) and the influence of different substituents on antimalarial activity. Manzamines SAR is summarized into two objectives. The first is the effect of various substitutions on the β-carboline nucleus, and the other is the effect of substitutions on the pentacyclic ring.^29^ The β-carboline moiety of manzamine alkaloids is responsible for antimalarial activity. 9-*N* alkylation of the β-carboline ring decreases antimalarial activity, indicating that 9-NH is necessary for their antimalarial activity. Hydroxyl-group substitution at position 8 of the β-carboline skeleton does not significantly affect its antimalarial activity.

Hence, 8-hydroxymanzamine (2) has the same effect as manzamine A, while manzamine F (3), a related derivative of manzamine A, is inactive. The eight-membered rings differ between the inactive manzamine F and the active manzamine A. The double bond reduction and/or the incorporation of a ketone group into the adjacent carbon is harmful to antimalarial activity. Likewise, hydroxyl group attachment at position 6 instead of position 8 has a negative effect on antimalarial activity, as shown by the lower potency of 6-hydroxy-manzamine A (4).^27^*In vitro* and in *vivo* studies, manzamines A and its 8-hydroxy derivative inhibited *P. falciparum* growth.^30^ Several total syntheses of manzamines have been accomplished.^29,31^

### 2.2. Neo-kauluamine

Neo-kauluamine (5) is a manzamine dimer, with a highly complex molecule composed of two units of manzamine fragmented by ether bonds between the eight-membered rings isolated from an unspecified genus of Indo-Pacific sponge (Petrosiidae, order Haplosclerida). Despite its structural complexity, neo-kauluamine displayed a strong efficacy *in vivo* and is considered an up-and-coming agent in malaria.^27^ Although this structure, like manzamine F (3), lacks the double bond in the eight-membered ring, it showed the same activity as manzamine A. The lack of antimalarial activity for 12,34-oxamanzamine A (6) suggested that the C-12 hydroxyl, the C-34 methine, or the 8-ring conformation are of great importance for the antimalarial activity.^28^

### 2.3. Zamamidines

Zamamidines A–D (7–10) are also manzamine alkaloids obtained from *Amphimedon* sp. sponge (*Niphatidae*). They had inhibitory activities against *P*. *falciparum* (IC_50_ values from 0.0008 to 0.016 μM).^32–34^ Zamamidine C (9) is the most active one of the series. Zamamidine D (10) is the first manzamine alkaloid characterized by having a moiety of 2,2′-methylene bis-tryptamine instead of a unit of β-carboline.^35^

### 2.4. Homofascaplysin A

Homofascaplysin A (11) is also β-carboline alkaloid. It was extracted from the *Hyrtios erecta* sponge (Thorectidae).^36^ This alkaloid presented potent activity against CQ-resistant *P. falciparum* strains (IC_50_ = 0.07 μM) with approximately 10-fold less cytotoxicity.^37^ This potent antiplasmodial activity of this compound demonstrated its potential as a lead structure among antimalarial agents and became a synthesis target for the production of other similar analogues.^38^

### 2.5. Marinacarbolines

Marinacarbolines A–D (12–15), series of β-carboline alkaloids were obtained from the fermentation broth of the marine actinomycete bacteria *Marinactinospora thermotolerans* (Nocardiopsaceae). Marinacarbolines displayed antiplasmodial activities against 3D7 and Dd2 lines of *P. falciparum*, with IC_50_ from 1.92 to 36.03 μM.^39^

### 2.6. Indolactam alkaloids

13-*N*-Demethyl-methylpendolmycin (16) and methylpendolmycin-14-*O*-α-glucoside (17) were derived from *Marinactinospora thermotolerans* (Nocardiopsaceae) fermentation broth. They were also found to exhibit moderate or weak activity against 3D7 (IC_50_ = 20.75 μM and 10.43 μM) and Dd2 (IC_50_ = 18.67 μM and 5.03 μM) strains of *P. falciparum*, respectively.^39^

### 2.7. Crambescidins

Crambescidin 800 (18) was obtained from the Indonesian sponge (*Mycophora* sp. Crambeidae). Crambescidin 800 showed IC_50_ of 160 nM and 240 nM, respectively, against the 3D7 and FCR3 lines of *P. falciparum*.^40^ Also, other alkaloids, including crambescidin 359 (19), crambescidin acid (20), and fromiamycalin (21), were extracted from the sponge *Monanchora unguiculate* collected in Madagascar. Crambescidin 359 was active against the 3D7 line of *P. falciparum* (IC_50_ = 0.52 μM).^41^ Additionally, Unguiculin A (22), an acyclic guanidine alkaloid, was detected in this sponge. In addition to four pentacyclic alkaloids ptilomycalins E–H (23–26) were also isolated. Among them, fromiamycalin (IC_50_ = 0.24 μM), unguiculin A (IC_50_ = 12.86 μM) ptilomycalins E (IC_50_ = 0.35 μM), F (IC_50_ = 0.23 μM), ptilomycalins G and H mixture (IC_50_ = 0.46 μM), respectively exhibited promising activity against *P. falciparum*.^41^

### 2.8. Opacalines

Opacalines are alkyl guanidine-substituted β-carboline-containing metabolites obtained from the New Zealand ascidian *Pseudodistoma opacum* (Pseudodistomidae). Opacalines B (27) and C (28) displayed moderate activity against the CQ-resistant *P. falciparum* strain (IC_50_ range of 2.5–14 μM).^42^

### 2.9. Spermidine

Two indole alkaloids, Didemnidines A (29) and B (30), were obtained from the New Zealand ascidian *Didemnum* sp. (Didemnidae). Among them, Didemnidine B showed mild activity (IC_50_ = 0.047 μM) against *P. falciparum*.^43^

### 2.10. Salinosporamide

Salinosporamide A (31) is a simple γ-lactam spiro-alkaloid isolated from an actinomycete bacteria belonging to the genus *Salinispora tropica* (Micromonosporaceae).^44^ Salinosporamide A is also a cyclic depsipeptide (bicyclic β-lactone γ-lactam peptide).

Salinosporamide A showed potential as an antimalarial candidate. It exhibited a potent parasite proteasome inhibitor and antimalarial activity against *P. falciparum in vitro* (IC_50_ = 11.4 nM) and *in vivo* against *P. yoelii*.^45,46^ By controlling T cell proliferation and leading to cell cycle arrest, Salinosporamide A suppressed T cell activation and regulated the expression of cyclin-dependent kinases.^47^ Recently total synthesis for salinosporamide A molecule has been reported.^46,48,49^

### 2.11. Bromotyrosine alkaloid

Several psammaplysin derivatives were obtained from the Indonesian marine sponge *Aplysinella strongylata* (*Aplysinellidae*).^50,51^ Psammaplysin H (32) is a bromotyrosine alkaloid from a marine sponge *Pseudoceratina* sp. and displayed more than 97% antimalarial activity (0.41 μM concentration).^52^ Psammaplysins H, F (33), and G (34) exhibited antimalarial activity, while Psammaplysins F and G displayed antimalarial activities against the drug-resistant strains of Plasmodium falciparum.^53^ Ceratinadins were bromotyrosine alkaloids that had the 1,6-dioxa-2-azaspiro[4.6] undecane skeleton, Ceratinadins E (35) and F (36), obtained from an Okinawan marine sponge *Pseudoceratina* sp. (Pseudoceratinidae). Ceratinadins E showed antimalarial activities against drug-resistant and drug-sensitive K1 P. falciparum strains.^54^

### 2.12. Pyrrolo-iminoquinones

Pyrroloiminoquinone compounds, Tsitsikammamine C (37), makaluvamines J (38), G (39), L (40), K (41), damirones A (42), and B (43), were extracted from the Australian marine sponge *Zyzzya* sp. (Acarnidae, order Poecilosclerida). All compounds were investigated against 3D7 and Dd2 *P. falciparum* strains. Among them, tsitsikammamine C with IC_50_ = 13 and 18 nM, respectively, inhibited both ring and trophozoite stages of the malaria parasite life cycle. Makaluvamines J, G, and L showed a potent antimalarial activity (IC_50_ < 100 nM) *in vitro* against both strains.^55^ A class of two new brominated pyrroloiminoquinones; dihydrodiscorhabdin B (44) and discorhabdin Y (45), along with six pyrroloiminoquinone alkaloids, discorhabdins A (46), C (47), E (48), and L (49), dihydrodiscorhabdin C (50) were obtained from Alaskan sponge genus *Latrunculia* (Latrunculiidae) among them, discorhabdins A, C, and dihydrodiscorhabdin C displayed antimalarial *in vitro* activity against both D6 and W2 *P. falciparum* strains.^56^

### 2.13. Imidazole alkaloids

Girolline (51), a 2-aminoimidazol derivative initially isolated from a Caledonian sponge *Cymbastela cantharella* (Axinellidae)^57^ showed antimalarial activity against *P. falciparum* (FCM29) strain of (IC_50_ = 0.13 μM) a high *in vivo* activity in a *P*. *vinckei petteri* rodent model.^58^

### 2.14. Thiazine-derived alkaloids

Four thiazine-pyrroloquinone containing tricyclic quaternary alkaloids, Thiaplakortones A–D (52–55), were obtained from the Australian sponge *Plakortis lita* (Plakinidae). Thiaplakortones A–D exhibited potent inhibition against 3D7 CQ-sensitive (IC_50_ = 51 nM, 0.65, 0.309, and 0.279 μM) and Dd2 CQ-resistant *P. falciparum* (IC_50_ = 6.6 nM 0.092, 0.171, and 0.159 μM), respectively.^59^

### 2.15. Terpenoid alkaloids

Several terpenoid alkaloids had been showing reasonable antimalarial activity. For instance, Monamphilectine A (56), a diterpenoid-lactam alkaloid from sponge *Hymeniacidon* sp., displayed action against the *P. falciparum* W2 strain (IC_50_ = 0.60 μM).^60^ Agelasines J (57), K (58), and L (59), three adenine terpenoids, were obtained from the sponge *Agelas mauritiana* (Agelasidae). They have weak antimalarial activity against the *P. falciparum* Columbian FcB1 strain (IC_50_ = 6.6, 8.3, and 18 μM, respectively).^61^ Netamines G–N (^60–67^) and tricyclic alkaloids were obtained from the Madagascar sponge *Biemna laboutei* (Desmacellidae). Netamine K showed antimalarial activity (IC_50_ = 2.4 μM) against *P. falciparum*.^62^

## 3. Terpenoids

All terpenes or terpenoids have fundamental repeating five-carbon isoprene units. Terpenes are classified as hemiterpenes (C_5_), monoterpenes (C_10_), sesquiterpenes (C_15_), diterpenes (C_20_), sesterterpenes (C_25_), triterpenes (C_30_), and tetraterpenes/carotenoids (C_40_).^63^ Marine-derived terpenoids have attracted potential interest similar to the terrestrial analogues represented by the sesquiterpene lactone artemisinin and isonitriles-containing terpenes.^7,64,65^ More than 30 compounds were isolated and showed antimalarial activity from marine organisms. Unique mechanisms were demonstrated, including the inhibitory activity against heme detoxification by isonitrile derivatives. They are discussed in the following subsections, and their chemical structures are shown in Table 3.

**Table 3:** A list of marine-derived terpenes-containing antimalarial drugs showing their IC_50_ against various strains of *Plasmodium* sp., chemical structure and biogenic source

Compound	Antiplasmodial activity (IC_50_ value)	Structure	Source	Marine class	Ref.
Axisonitrile-1 (68) Axamide-1 (69)	W2 = 0.073 μM D6 = 0.61 μM	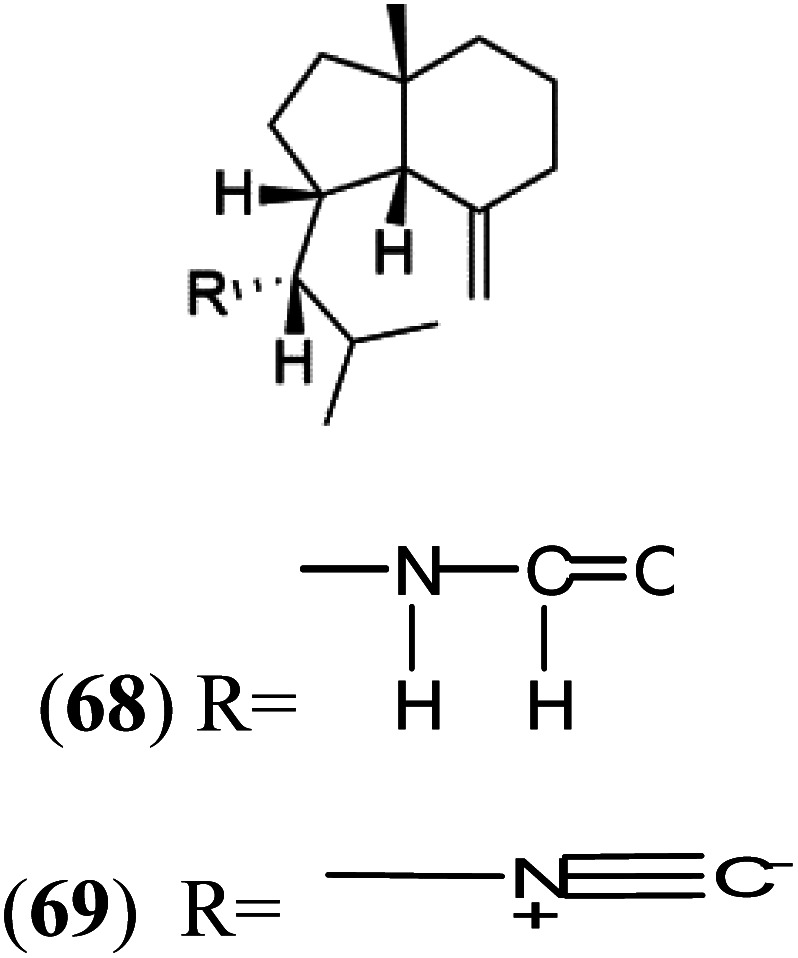	*Axinella cannabina*	Sponge	67 and 69–72
Axamide-2 (70) Axisonitrile-2 (72) Axisothiocyanate-2 (74)		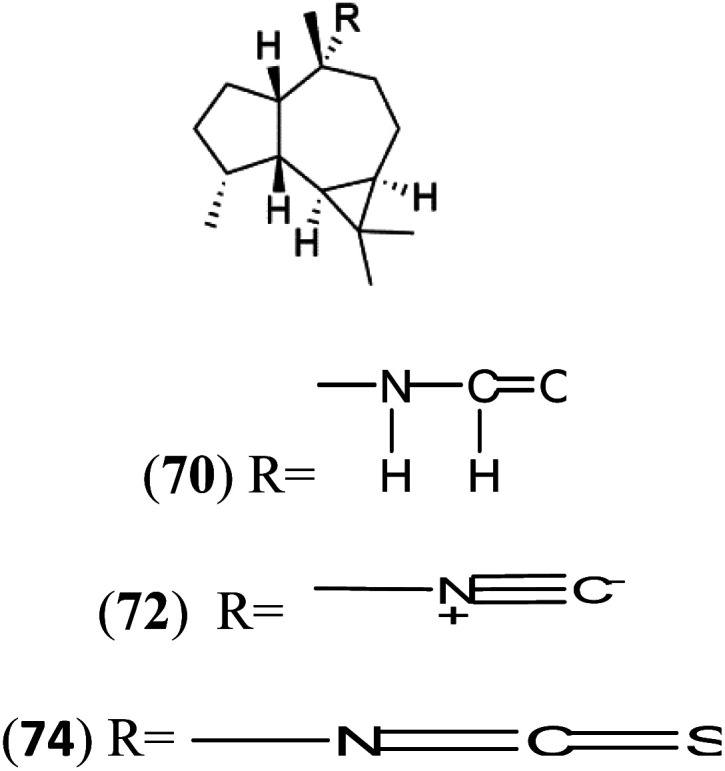			
Axamide-3 (71) Axisonitrile-3 (73) Axisothiocyanate-3 (75)		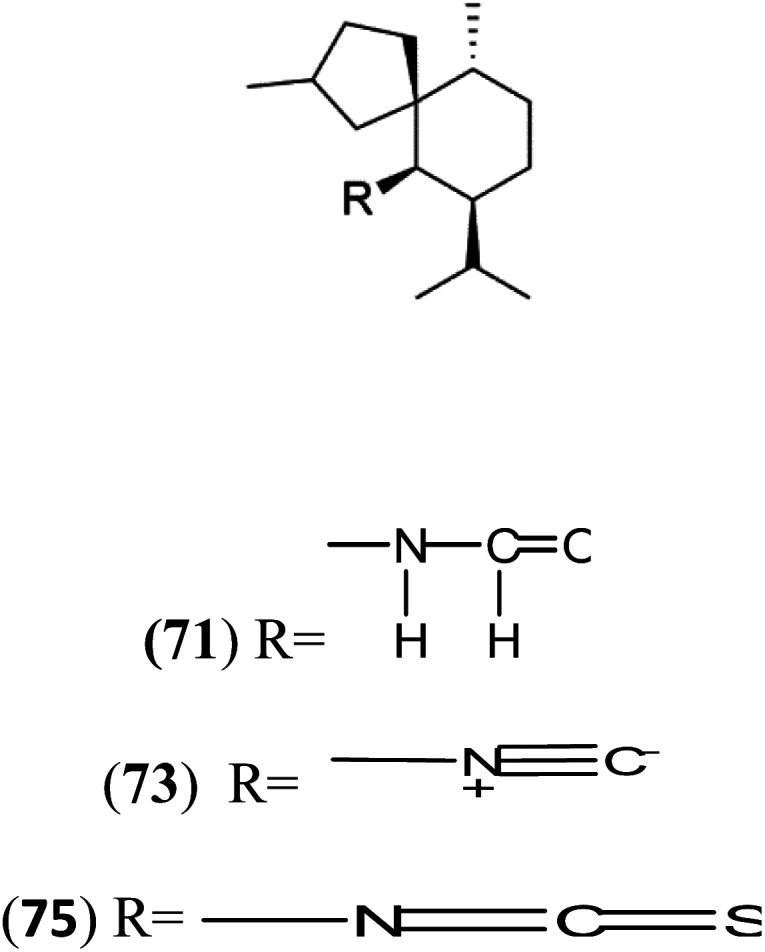			
(−)-8,15-Diisocyano-11(20)-amphilectene (76)	W2 = 15 nM D6 = 16 nM K1 = 90 nM	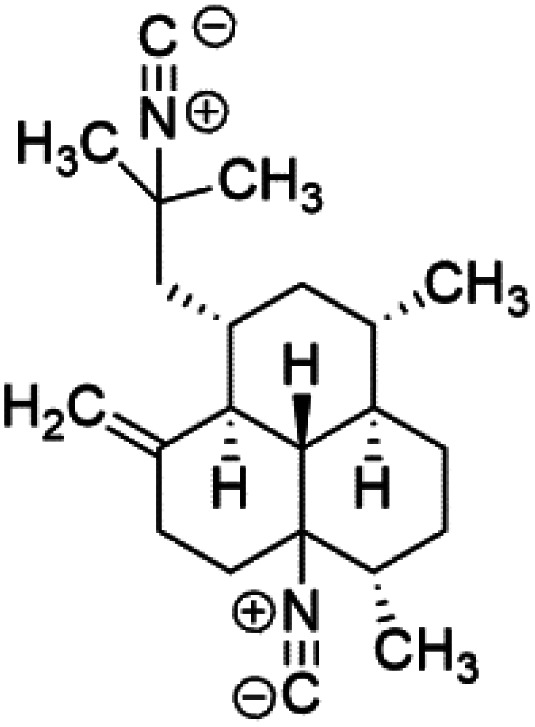	Hymeniacidon amphilecta, Venzea flava	Sponge	73 and 74
Kalihinol A (77)	FCR-3 = 12 μM	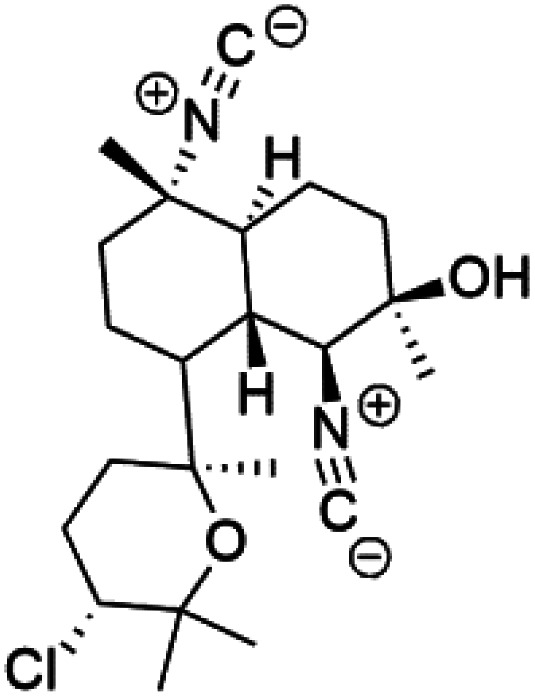	*Acanthella* sp.	Sponge	68
(8*R*)-8-Bromo-10-epi-β-snyderol (78)	D6 = 0.012 and W2 = 0.017 μM	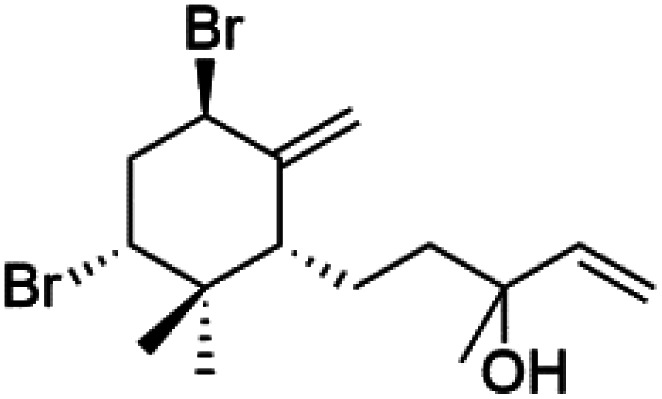	*Laurencia obtusa*	Red alga	77
Chloroaureol (79) Aureol (80) Aureol acetate (81)	(79) D6 = 9.74 μM (80) and (81) NA	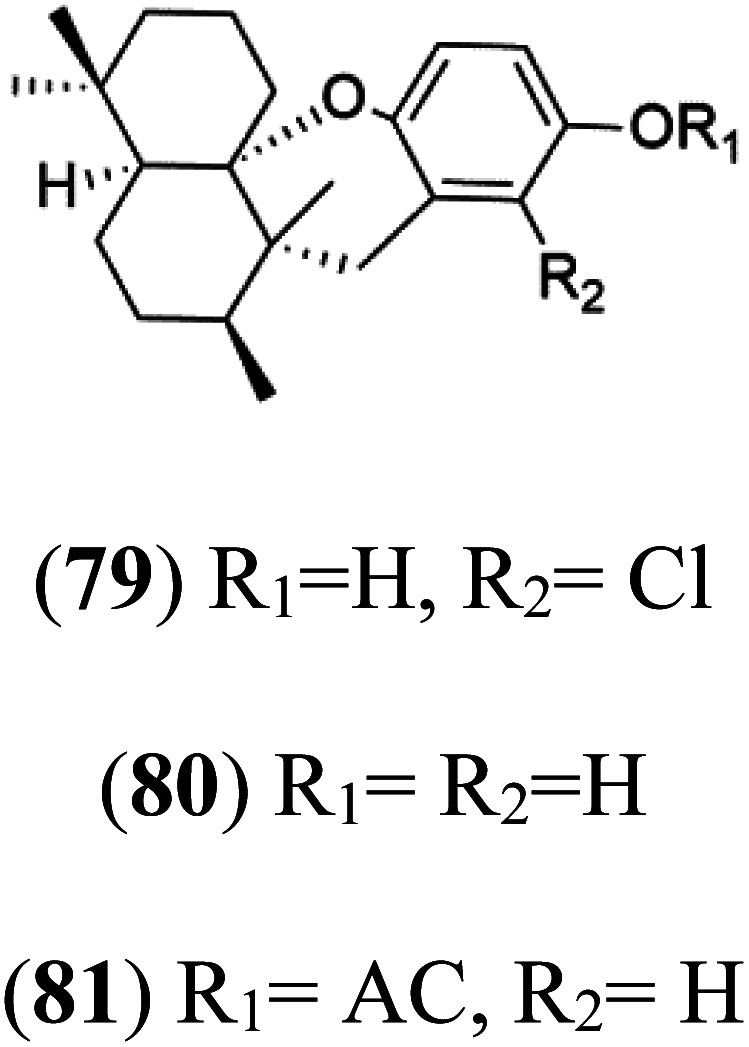	*Smenospongia aurea*	Sponge	78
Pelorol (82)	NF54 = 0.005 and K1 = 0.0019 μM	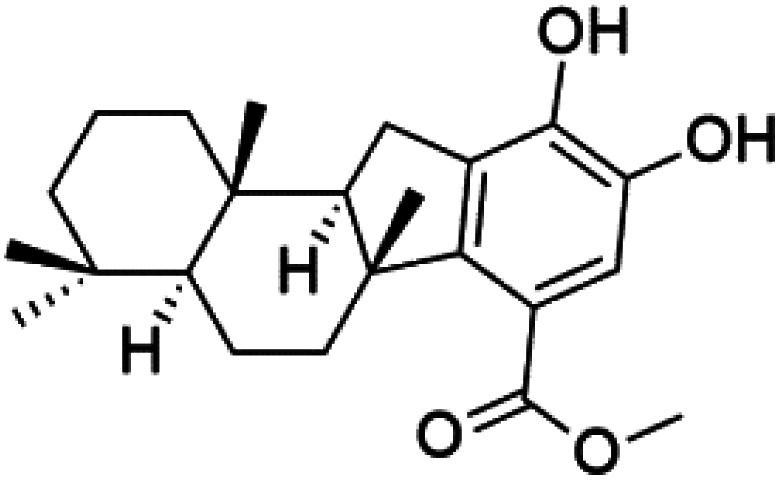	*Dactylospongia elegans*	Sponge	79
Ilimaquinone (83)	NF54 = 0.0026 K1 = 0.0048 μM	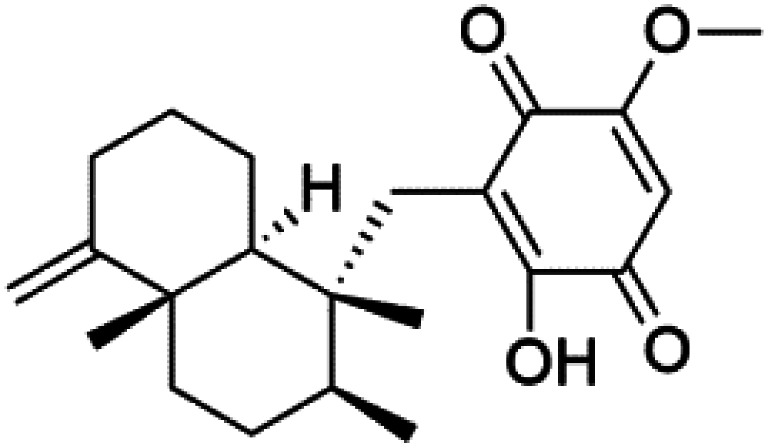			79
Isocyanoclovane (84)	3D7 = 300, 290, and 260 nM Dd2 = 360, 830, and 870 nM	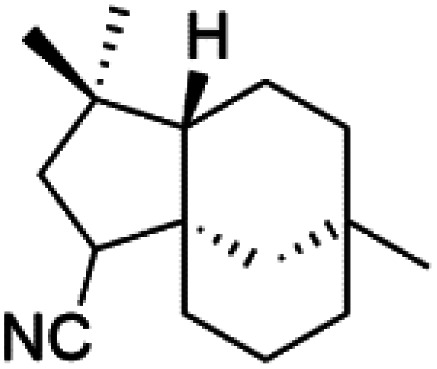	*Phyllidia ocellate*	Nudibranch	80
2-Isocyanoclovene (85)		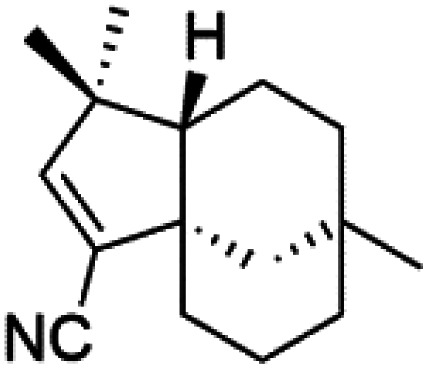			
4,5-Epi-10-isocyanoisodauc-6-ene (86)		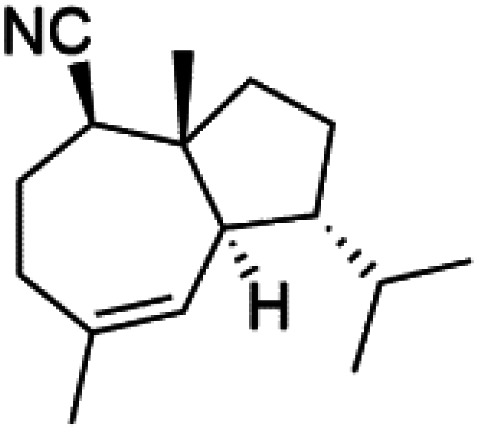			
8a,11-Dihydroxypachydictoyl A (87)	*K*1 = 10.0 μM	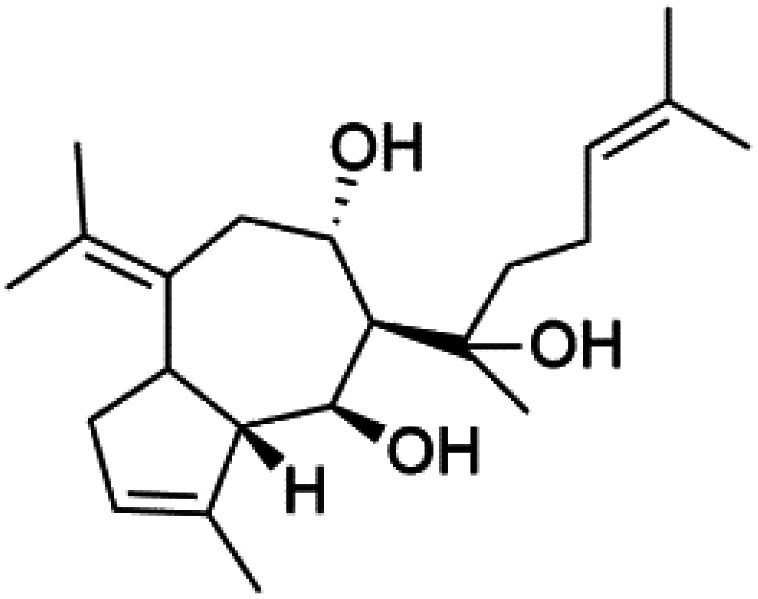	*Dictoyta* sp.	Brown alga	83
4,18-Dihydroxydictyolactone (88)		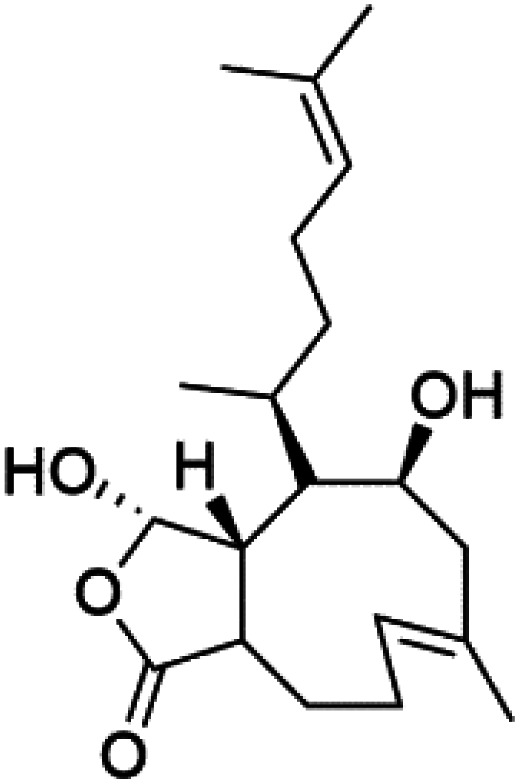			
Laevigatol A (89)	Dd2 IC_50_ < 5.0 μM	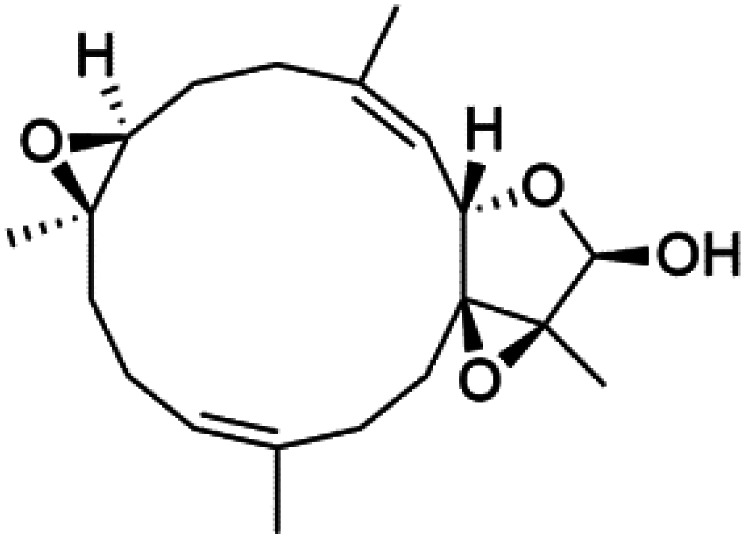	*Pseudopterogorgia elisabethea*	Soft coral	84 and 85
Pseudopterosin V (90)	Dd2 = 2.2 μM	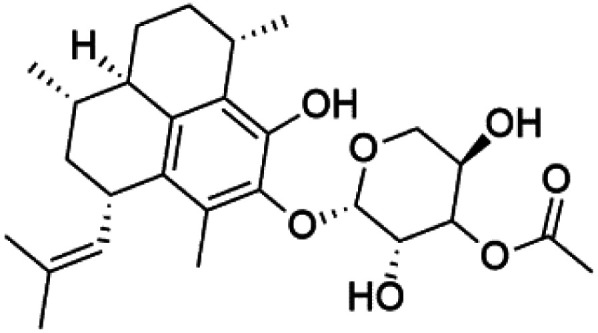	*Pseudopterogorgia elisabethea*	Soft coral	86
Dorisenone D (91)	K1 = 1.3 μM	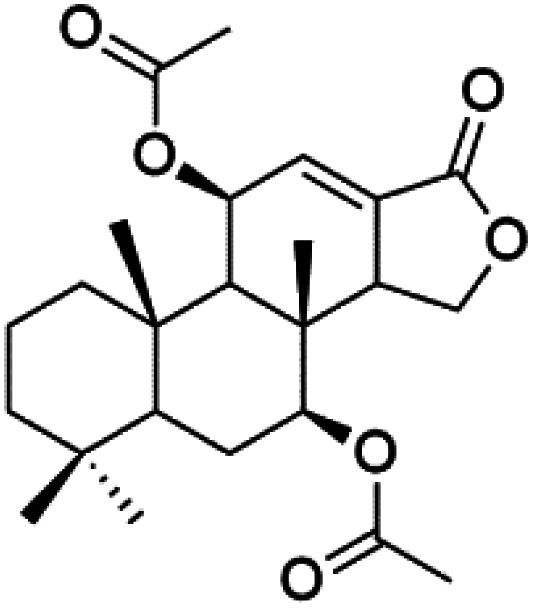	*Dysidea arenaria*	Sponge	87

### 3.1. Sesquiterpene isonitriles

Sesquiterpenoid metabolites containing isonitrile, isothiocyanate, and formamide were isolated for the first time from *Axinella cannabina* sponge (Axinellidae).^66,67^ Isonitrile-containing compounds do their activity by blocking heme (FP) detoxification. The interaction of isonitrile with free heme was demonstrated by forming a coordination complex with the iron center. The drug carrier must possess a solid lipophilic molecular nucleus with at least a tricyclic structure that carries an isonitrile group and establishes further hydrophobic reactions above the ring plane. The interaction of marine isonitrile derivatives with heme can suppress the sequestration of FP into beta-hematin and prevent the peroxidative and glutathione-mediated destruction of FP under conditions designed to imitate the environment inside the malaria parasite.^68^ Axisonitrile-1 (68) was the parent compound of the class of isonitrile containing sesquiterpenoids marine secondary metabolites obtained from *A. cannabina* sponge.^67,69^

Isolation of axisonitrile-1 was followed by other related sesquiterpenoids from the same sponge, as axamide-1 (69), axamide-2 (70), axamide-3 (71), axisonitrile-2 (72), axisonitrile-3 (73), axisothiocyanate-2 (74), axisothiocyanate-3 (75).^70–72^ In 1978, (−)-8,15-diisocyano-11(20)-amphilectene (76) was first reported from *Hymeniacidon amphilecta* (Halichondriidae) and was subsequently shown to demonstrate potent anti-infective activity *in vitro*.^73,74^ The closely related axisothiocyanate-3 was inactive, indicating that the activity depends not only on the carbon skeleton's structural characteristics but also directly on the existence of the isonitrile group. The action was confirmed by comparison among the activities of related compounds. It showed that the biological activity generally depends on the isocyanide functionality and the carbon skeleton structural features. The isocyanide group's location also has a pivotal role.^75^

Bis-isonitrile-containing product and isocyanoterpene members, Kalihinols, were isolated from a Guam sponge, *Acanthella* sp.^68,76^ Many natural Kalihinol products are potent inhibitors of *P. falciparum*. Hence its importance, several Kalihinol analogs were synthesized and investigated using drug-sensitive and resistant *P. falciparum* for blood-stage antimalarial activity.^68^ Kalihinol A (77) showed increased potency and activity against the FCR-3 strain (Kalihinol A EC_50_ = 1.2 nM).^67^. (8*R*)-8-Bromo-10-epi-β-snyderol (78), a sesquiterpene isolated from *Laurencia obtuse* (Rhodomelaceae) red alga displayed activity against *P. falciparum* D6 and W2 strains (D6 = 0.012 and W2 = 0.017 μM), respectively.^77^

6′-Chloroaureol (79), aureol (80) and aureol acetate (81), sesquiterpene-phenol were obtained from *Smenospongia aurea* sponge.^78^ These derivatives displayed reasonable activity against *P. falciparum* D6 strain. Another marine sesquiterpene, pelorol (82), and sesquiterpene quinone, ilimaquinone (83), were extracted from the sponge *Dactylospongia elegans* (Thorectidae). Both showed moderate antiplasmodial activity against K1 and NF54 strains of *P. falciparum*.^79^

Sesquiterpenes, 2-isocyanoclovene (84) and 2-isocyanoclovane (85) were obtained from the Australian nudibranch *Phyllidia ocellate* (Phyllidiidae). They showed activity against 3D7 (IC_50_ = 300, 290, and 260 nM, respectively) and Dd2 *P. falciparum* clones (IC_50_ = 360, 830, and 870 nM, respectively).^80^

### 3.2. Diterpene isonitriles

A phytochemical investigation of the *Cymbastela hooperi* sponge provided 15 diterpenes containing isonitriles isocyanate, isothiocyanate, and isonitrile functionalities, which showed higher antimalarial effect and moderate toxicity.^81^ The activity of analogues with isocyanate and isothiocyanate functionality was up to ten times lower, demonstrating that the isonitrile group improved activity. An analogue containing only the formamide functional group but no isonitrile group was also ineffective against *P. falciparum*, implying that the formamide group isn't necessary for antiplasmodial efficacy.^82^ Monamphilectine A (56) was a diterpenoid β-lactam alkaloid separated from a *Hymeniacidon* sp. sponge (Halichondriidae). Monamphilectine A displayed a potent antimalarial activity,.^60^ While (−)-8,15-diisocyano-11(20)-amphilectene (86), re-isolated from the *Svenzea flava* sponge (Scopalinidae), was used as a precursor to synthesize five new products, all of which were tested against laboratory colonies of *P. falciparum* and *Mycobacterium tuberculosis* H_37_Rv.^75^

In addition, 8a,11-dihydroxypachydictoyl A (87), and 4,18-dihydroxydictyolactone (88) were diterpenoids isolated from *Dictoyta* sp. of the brown alga, which displayed antimalarial activity (IC_50_ = 10.0 μM) against K1 strain of *P. falciparum*.^83^

Moreover, soft corals and echinoderms living in Vietnamese seas provided several diterpenes. Among them is laevigatol A (89), which had a moderately antiplasmodial activity with an IC_50_ < 5.0 μM.^84,85^ Also, a series of diterpene glycosides was obtained from the Caribbean soft coral *Pseudopterogorgia elisabethea* (Gorgoniidae). Among them pseudopterosin V (90) exhibited an antimalarial activity (IC_50_ = 2.2 μM) against CQ-resistant *P. falciparum* colonies.^86^

### 3.3. Other terpenoids

Screening of marine sponges extracts from *Spongia*, and *Ircinia* genera revealed thr presence of broad-spectrum antiprotozoal meroterpenes, linear triterpenoid, and squalene, with inhibitory effects on *P. falciparum* and *Trypanosoma*. The dorisenone D (91), a dimeric C_21_ meroterpenoid obtained from *Dysidea arenaria* sponge (Dysideidae), may become a promising antiplasmodial compound.^87^

## 4. Endoperoxide-containing compounds

One of the most fundamental advances in malaria chemotherapy was the discovery and manufacturing of endoperoxide-containing drugs. Undoubtedly, the artemisinin discovery was the beginning of research in this area. Artemisinin is a cadinane sesquiterpene lactone establishing a 1,2,4-trioxane moiety isolated from sweet wormwood *Artemisia annua* L. leaves (Asteraceae). Artemisinin showed nanomolar potency against CQ-resistant *Plasmodium* strains. The endoperoxide linkage is essential for antimalarial activity. One of the artemisinin derivatives lacking the endoperoxide bridge showed no antimalarial activity.^88^

These drugs containing endoperoxide were purported to interact *via* endoperoxide bond with the iron(ii) center of the heme unit released in the food vacuole during the digestion of hemoglobin and lead to peroxide bridge cleavage the consequent formation of oxygen-centered radicals. Because of an intramolecular rearrangement, these reactive species were converted into free C-centered radicals, toxic to the parasite through the alkylation of sensitive macromolecular targets. A sarco-endoplasmic reticulum Ca^2+^ dependent ATPase of *P. falciparum* has been proposed as a possible target for these active species.^89,90^ Although, most likely, artemisinin activity is not mediated by interaction with a single enzyme. It had been proposed that the Fe^2+^-containing species interacting with the endoperoxide bond is not heme.^91^ The marine antimalarial drug-containing endoperoxide was divided according to their structural feature into peroxyketal and non-peroxyketal, as demonstrated below, and the chemical structures are listed in Table 4.

**Table 4:** A list of marine-derived endoperoxide-containing antimalarial drugs^a^

Compound	Antiplasmodial activity (IC_50_ value)	Structure	Source	Marine class	Ref.
Peroxyplakoric acids A_3_ (92)	FCR3 = 150 and 120 nM, respectively	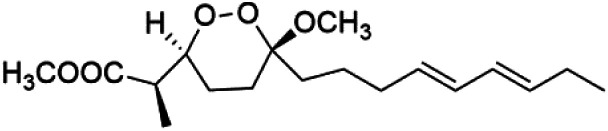	*Plakortis* sp.	Sponge	92 and 93
Peroxyplakoric acid B_3_ (93)		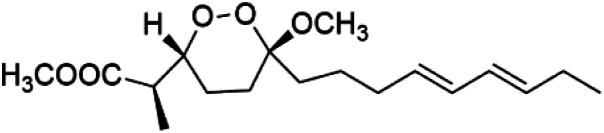			
Plakortin (94)	W2 = 0.16 nM	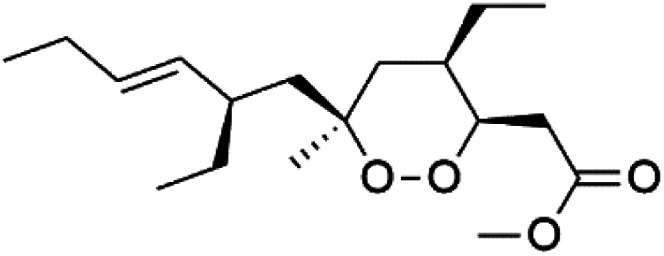	*Plakortis halichondroides, Plakortis simplex*	Sponge	96
Dihydroplakortin (95)	D10 = 1.12 μM W2 = 0.76 μM	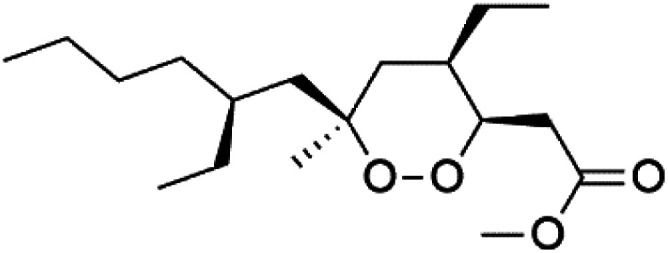			
3-Epiplakortin (96)	NA	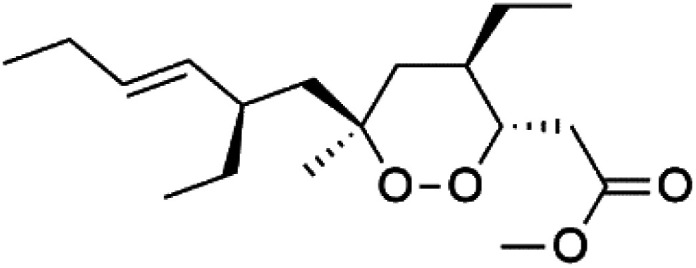			
Plakortide Q (97)	D10 = 1000 nM W2 = 520 nM	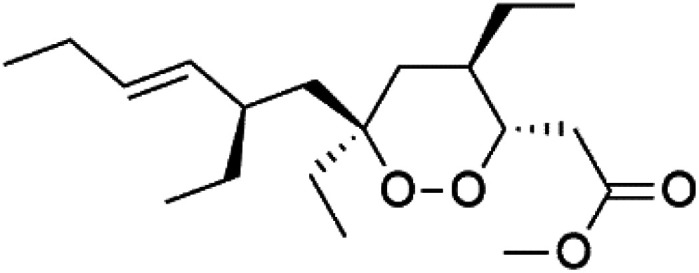			
Plakortide E (98)	D6 = 1.37 nM W2 = 1.11 nM	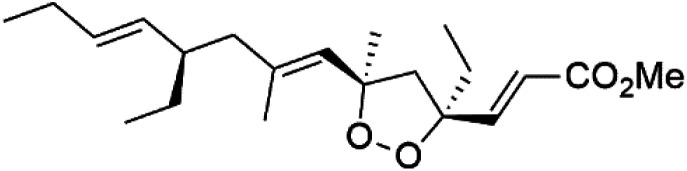			
Plakortide L (99)	NA	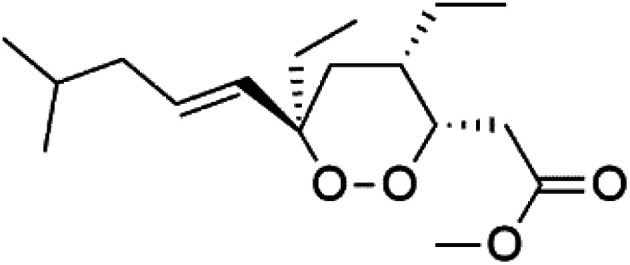	*Plakortis* sp.	Sponge	97 and 98
Plakortide O (100)	NA	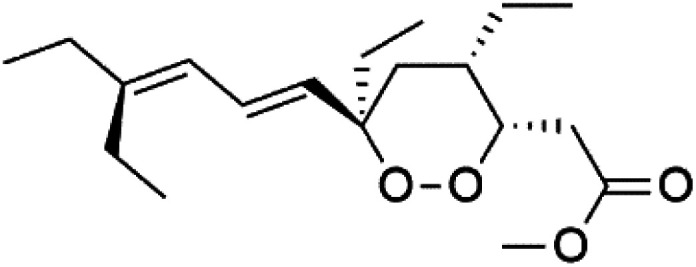			
Plakortide P (101)	NA	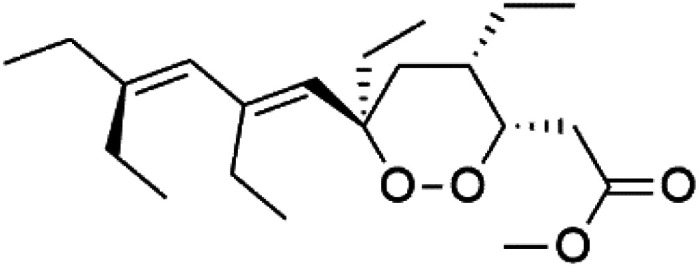			
Plakortin diol (102)	FcM29 = 800 nM	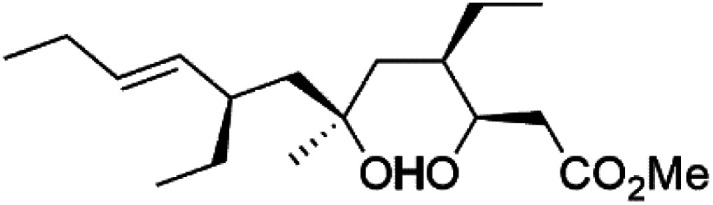			101
Plakortin hydroxyl (103) Plakortin methoxy (104) Plakortin acetoxy (105)	D10 = 1.26 nM	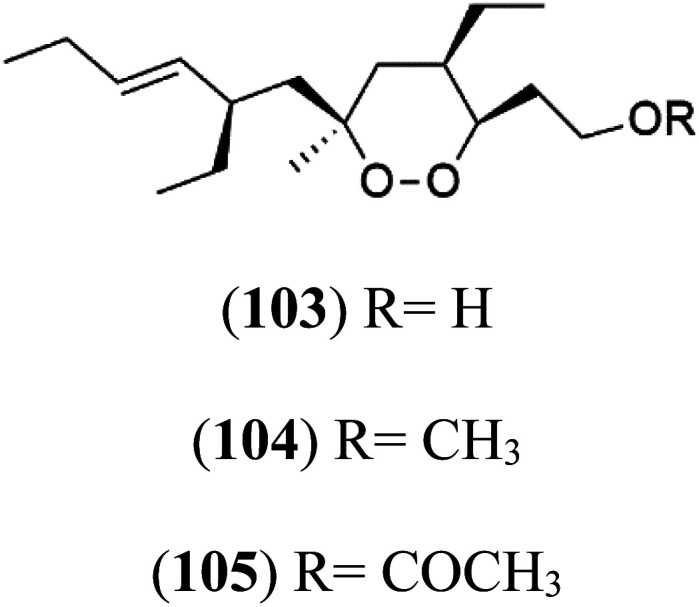			

a NA: not available.

### 4.1. Peroxyketal

Peroxyplakoric acids are the parent compound in the class of peroxyketals/3-alkoxy-1,2-dioxane derivatives, and its methyl esters; peroxyplakoric acids A_3_ (92) and B_3_ (93). It was extracted from the Okinawan sponge of *Plakortis* sp. (Plakinidae).^92,93^ Peroxyketals derivatives showed potent activity *in vitro* (IC_50_ = 150 and 120 nM against *P. falciparum* FCR3) and a good selective toxicity index. The long alkyl side chain in these derivatives is important for antimalarial activity as the synthetic analog containing methyl group instead of the nonadienyl group was completely nonactive. It has been observed that transforming the ester group into an amide group increases *in vivo* antimalarial potency.^94^

### 4.2. Non-peroxyketal

Plakortin (94) is a simple 1,2-dioxane metabolite, and was isolated from *Plakortis halichondroides*.^95^ Plakortin and its analogues, named dihydroplakortin (95), 3-epiplakortin (96), plakortide Q (97), plakortide E (98), were re-isolated from the Caribbean sponge *Plakortis simplex*.^96^ All these compounds, except plakortide E, displayed good antimalarial activity against (D10) CQ-sensitive and (W2) CQ-resistant strains of *P. falciparum*, with no cytotoxicity and a more potent activity on the (W2) strain (IC_50_ in D6 = 1.37 nM, and W2 = 1.11 nM). Plakortide E was found to be inactive. It could be ascribed to a five-membered ring presence instead of a six-membered ring and/or the crowded substituents at carbons flanking the endoperoxide linkage.^97,98^ Currently, plakortin is among pre-clinically investigated antiplasmodial candidates.^99^

On the other side, plakortide L (99), was extracted from a Jamaican sponge *Plakortis* sp.^97,98^ Plakortide O (100) and plakortide P (101), were isolated from plakortis halichondrioides, presented mild antimalarial activity *in vitro* (IC_50_ > 0.023 μM). Despite of their similarities with the plakortin, the configurational changes around the dioxane ring and/or the differences in the alkyl side chains are responsible for the observed decrease of activity.^100^ It was established that the role of the endoperoxide system in the antimalarial activity of plakortin derivatives is pivotal, as the diol derivative (102) with an open peroxide ring exhibited no antimalarial activity, when the ester group substitution to the corresponding hydroxyl (103), methoxy (104), or acetoxy (105) derivatives affected the antimalarial activity or the selectivity of these derivatives.^101^

## 5. Quinones, polyketides, phenols, and acids

Several phenolic compounds with antimalarial activity have been isolated from different marine sources. For instance, from *Hyrtios* sponge (Thorectidae), 5-oxopuupehenol (106) was isolated. It exhibited antimalarial activity against D6 and W2 clones of *P. falciparum*.^102^ In addition, three xanthones were isolated from the marine fungi *Chaetomium* sp. (Chaetomiaceae), *i.e.*, chaetoxanthones A (107), B (108), and C (109). Chaetoxanthones A and B contain a dioxane-tetrahydropyran moiety, while chaetoxanthone C is a chlorinated xanthone containing a tetrahydropyran ring. Among them, chaetoxanthone B exhibited a significant antimalarial activity (IC_50_ = 1.4 nM).^103^

A family of monoterpene-quinones, xestoquinone, and alisiaquinones, showed promising antimalarial activities. Xestoquinone (110) was obtained from a Vanuatu Pacific marine sponge *Xestospongia* sp., while alisiaquinone A (111), B (112), C (113), and alisiaquinol (114) were extracted from an unidentified deep-water Caledonian sponge. Xestoquinone was a selective active inhibitor of a protein kinase (Pfnek-1) of *P. falciparum* with IC_50_ = 1 μM. Alisiaquinone C presented activity against F32, FcB1, and FcM29 of *P. falciparum* (IC_50_ = 0.15, 0.21, and 0.08 μM, respectively).^104,105^ From the marine-derived fungus *Fusarium* sp., a series of secondary metabolites such as 9α-hydro-xyhalorosellinia A (115), bostrycin (116), nigrosporin B (117), javanicin (118), and anhydrofusarubin (119) were isolated.^106^

Among marine fungi, phytochemical investigation of *Halorosellinia oceanica* BCC 5149 resulted in isolation of different compounds such as 2-hexylidene-3-methylsuccinic acid, cytochalasin Q, 5-carboxymellein, 2-hexylidene-3-methylsuccinic acid 4-methyl ester, and halorosellinic acid. Cytochalasin Q, 5-carboxymellein, halorosellinic acid, and its acetonide derivative showed antimalarial activity with IC_50_ values of 17, 4, 13, and 19 μg mL^−1^, respectively.^107^ Also, the tetramic acid called vermelhotin was isolated from an unidentified fungus CRI247-01 (a member of the order Pleosporales). Vermelhotin exhibited moderate antiplasmodial activity 1–10 μg mL^−1^.^108^ Besides, marine sponges (*e.g.*, a Vanuatu marine sponge *Pseudoceratina* sp.) produced homogentisic acid derivative acting as protein kinase inhibitors Pfnek-1 with an IC_50_ about 1.8 μM and moderately active *in vitro* against a FcB1 *P. falciparum* strain (IC_50_ = 12 μM).^109^ Homogentisic acid is among pre-clinically investigated antiplasmodial candidates.^99^

Furthermore, (*S*)-curcuphenol (120) is a sesquiterpene phenol isolated from the Jamaican sponge *Didiscus oxeata*, displayed *in vitro* activity against D6 and W2 strains of *P. falciparum* (MIC = 0.017 and 0.008 μM, respectively).^110^ (2*Z*,6*R*,8*R*,9*E*)[3-ethyl-5-(2-ethyl-hex-3-enyl)-6-methyl-5*H*-furan-2-ylidene]-acetic acid methyl ester (121), a polyketide was isolated from *Plakortis angulospiculatus* sponge. It showed mild antimalarial activity against D6 and W2 *P. falciparum* colonies (IC_50_ = 6.6 nM on both strains).^111,112^

Also, gracilioether A–C (122–124), a polyketal peroxide obtained from the crude extract of the deep sea *Agelas gracilis* sponge (Agelasida), presented an antimalarial activity (IC_50_ = 28.22 μM).^113^

Recently, the angucyclines Actinosporins E, H, G (125–127), Tetrangulol (128), and Capillasterquinone B (129) have been isolated based on antimalarial guided fractionation of the co-cultured fermentation of the marine bacterium *Actinokineospora spheciospongiae* (Pseudonocardiaceae). Nevertheless, these compounds have not been reported under axenic conditions. Upon antimalarial screening, they displayed activity ranging from IC_50_ = 0.019 to 0.028 μM.^114^ The chemical structure and source are summarized in Table 5.

**Table 5:** A list of marine-derived quinones, polyketides, and phenols antimalarial drugs showing their IC_50_ against various strains of *Plasmodium* sp., chemical structure and biogenic source

Compound	Antiplasmodial activity (IC_50_ value)	Structure	Source	Marine class	Ref.
5-Oxopuupehenol (106)	D6 = 5.8 μM W2 = 3.8 μM	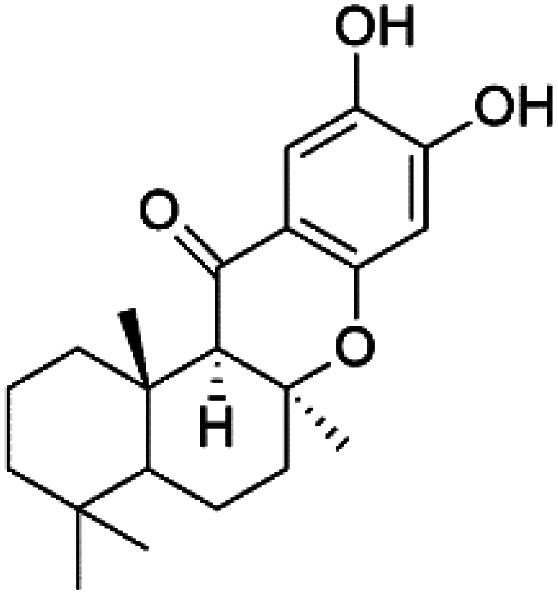	*Hyrtios* sp.	Sponge	102
Chaetoxanthone A (107) Chaetoxanthone B (108)	K1 = 1.4 nM	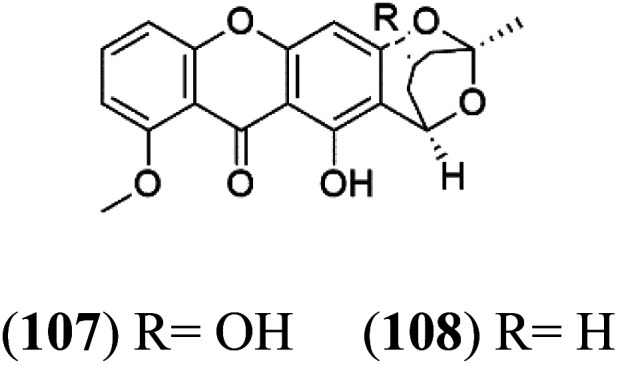	*Chaetomium* sp.	Marine fungi	103
Chaetoxanthone C (109)	NA	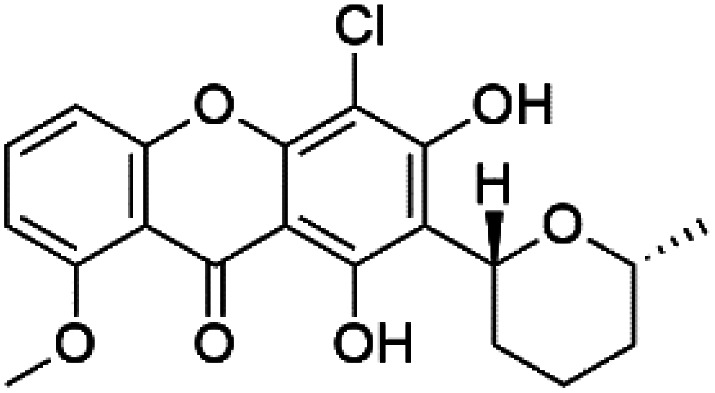			
Xestoquinone (110)	FcB1 = 3.0 μM	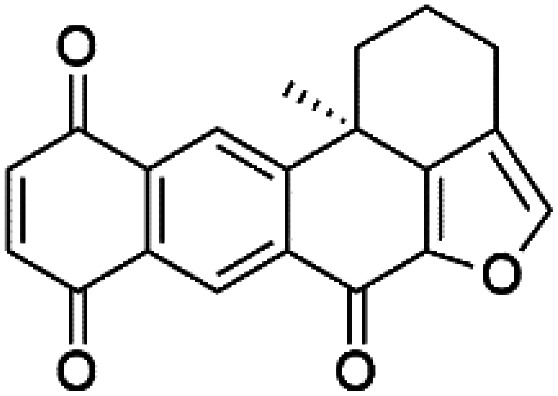	*Xestospongia* sp.	Sponge	104 and 105
Alisiaquinone A (111) alisiaquinone B (112)	FcMC29 = 8.50, 2.60 μM FcB1 = 7.40, 8.40 μM F32 = 9.10, 7.10 Mm, respectively	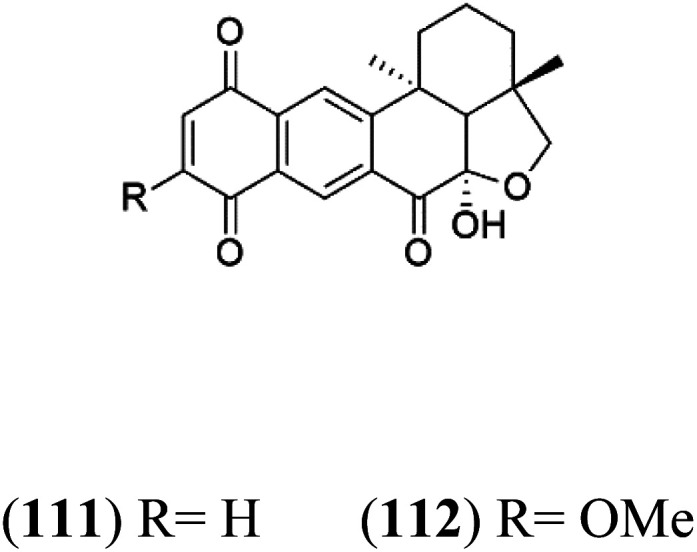	Unidentified deep-water caledonian sponge	Sponge	104 and 105
Alisiaquinone C (113)	FcMC29 = 0.08 μM FcB1 = 0.21 μM F32 = 0.15 μM	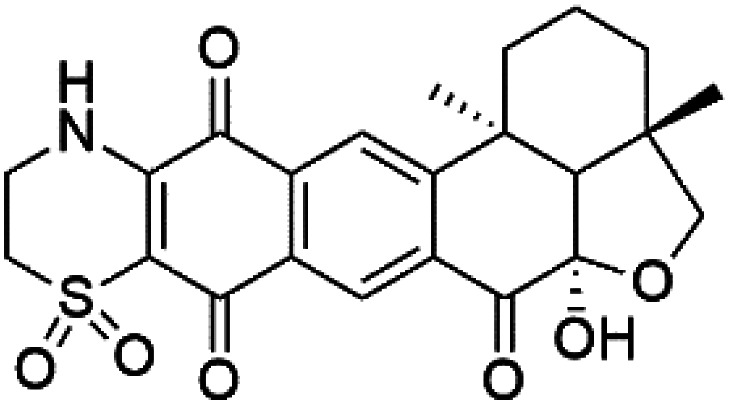			
Alisiaquinol (114)	FcMC29 = 7.90 μM FcB1 = 6.40 μM F32 = 9.90 μM	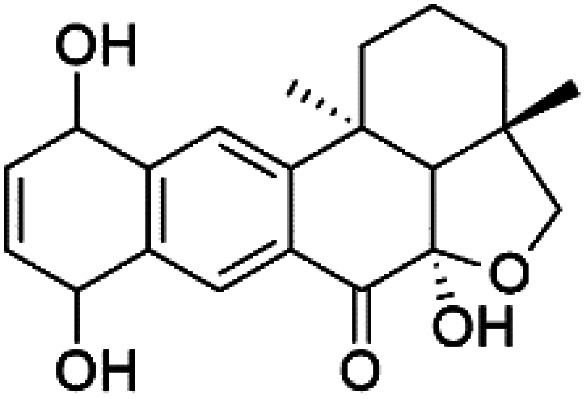			
9α-Hydro-xyhalorosellinia A (115)	K1 = 25 μM	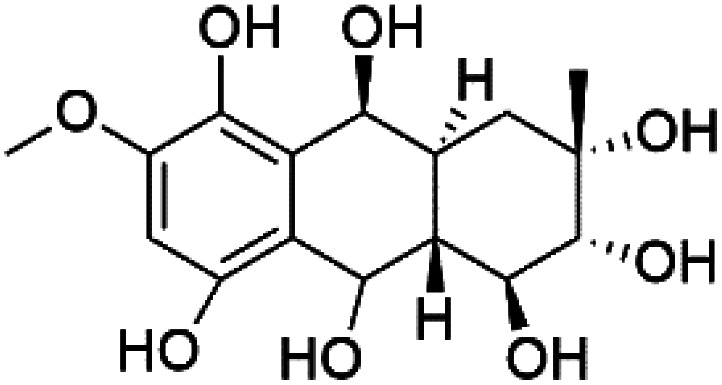	*Fusarium* sp.	Marine fungi	106
Bostrycin (116) Nigrosporin B (117)	K1 = 9.8 μM (116) NA for (117)	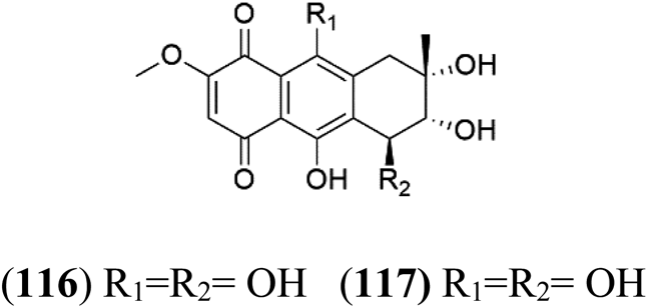			
Javanicin (118)	K1 = 12 μM	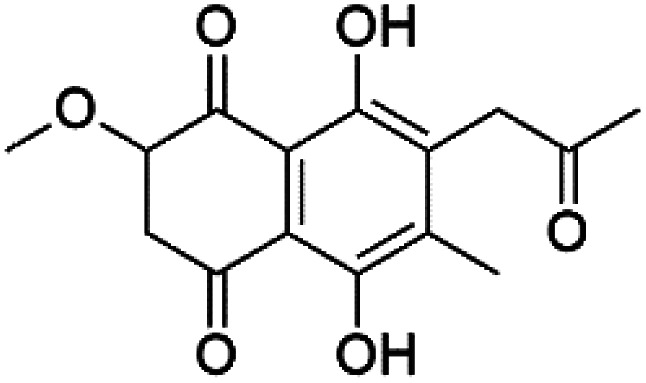			
Anhydrofusarubin (119)	K1 = 14 μM	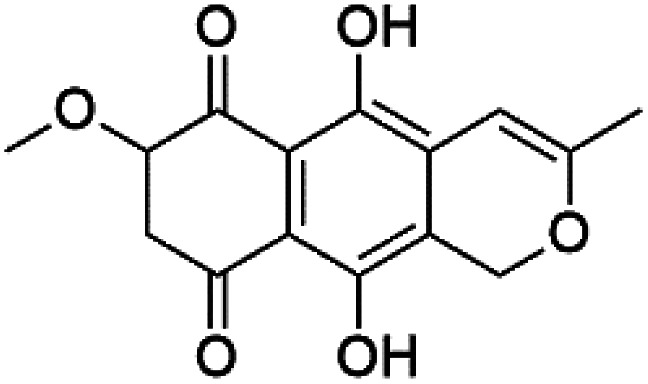			
(*S*)-Curcuphenol (120)	D6 = 0.017 μM W2 = 0.008 μM	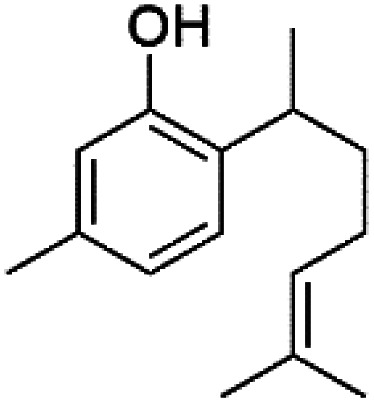	*Didiscus oxeata*	Sponge	110
(2*Z*,6*R*,8*R*,9*E*)[3-ethyl-5-(2-ethyl-hex-3-enyl)-6-methyl-5*H*-furan-2-ylidene]-acetic acid methyl ester (121)	D6 = 6.6 nM W2 = 6.6 nM	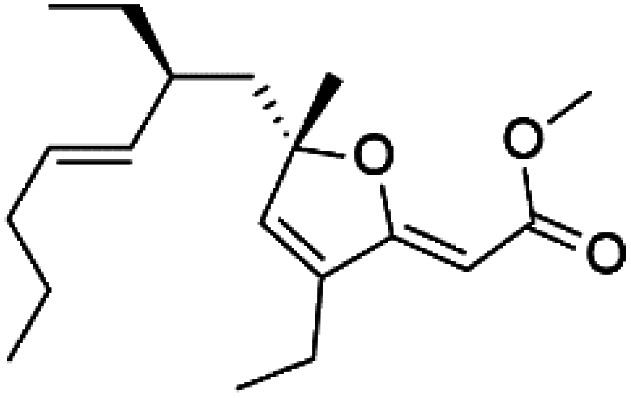	*Plakortis angulospiculatus*	Sponge	111 and 112
Gracilioether A (122)	ItG = 28.22 μM	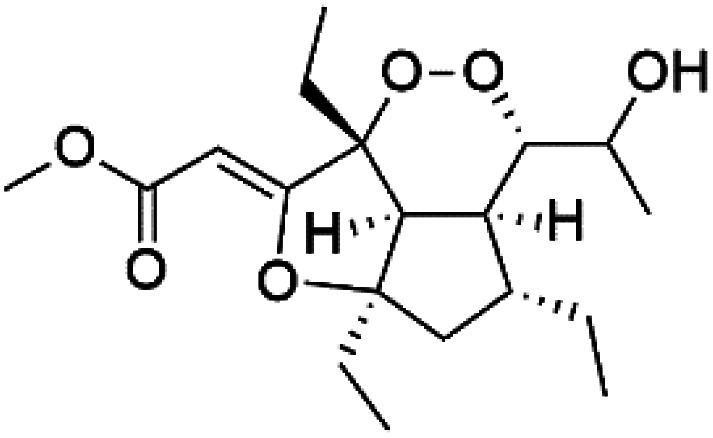	*Agelas gracilis*	Sponge	113
Gracilioether B (123)	ItG = 1.56 μM	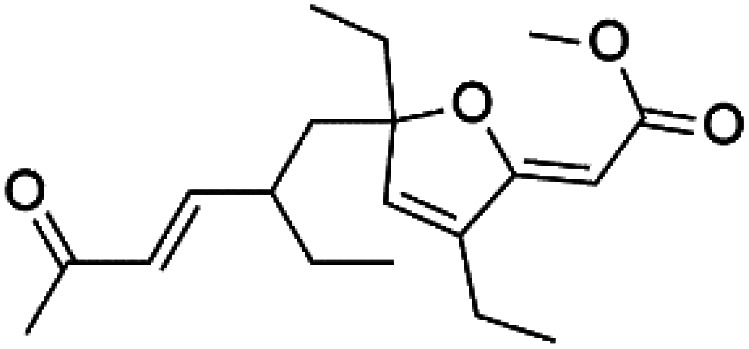			
Gracilioether C (124)	ItG = 31.02 μM	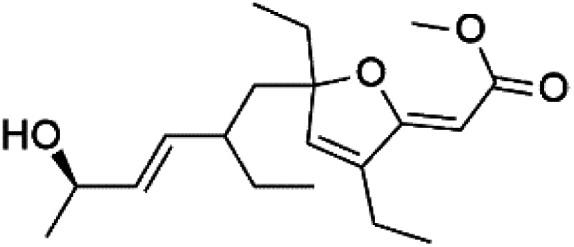			
Actinosporin E (125)	IC_50_ = 0.019 to 0.028 μM	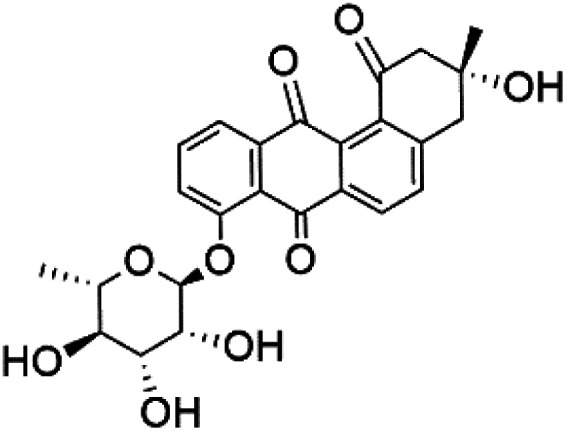	*Actinokineospora spheciospongiae*	Actinomycete bacteria	114
Actinosporin H (126)		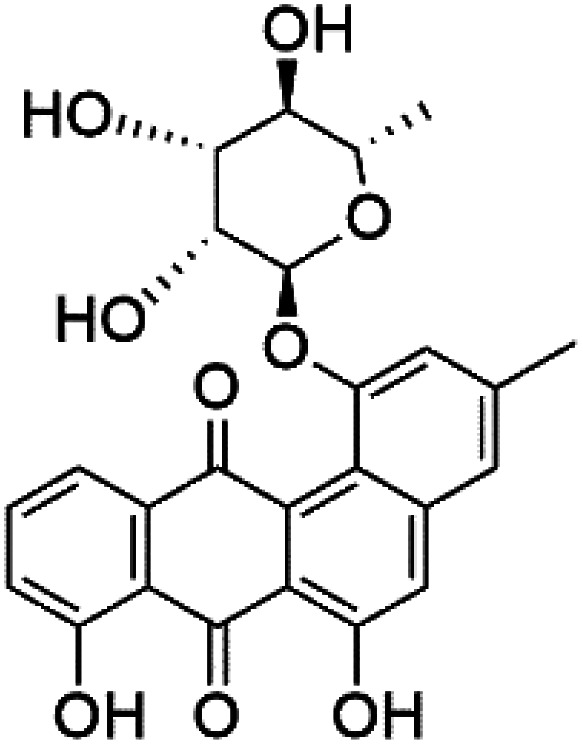			
Actinosporin G (127)		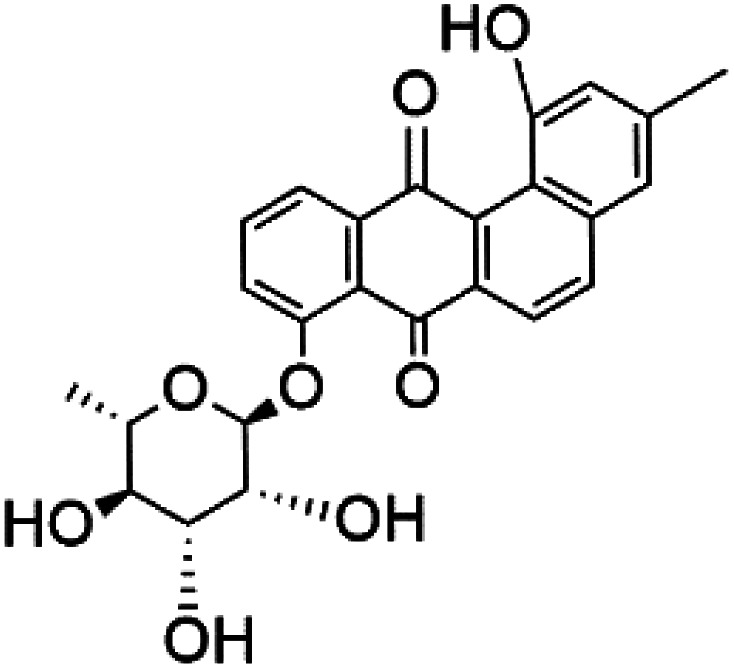			
Tetrangulol (128)		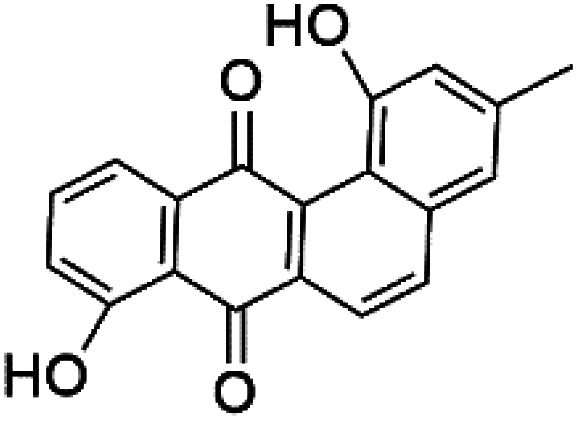			
Capillasterquinone B (129)		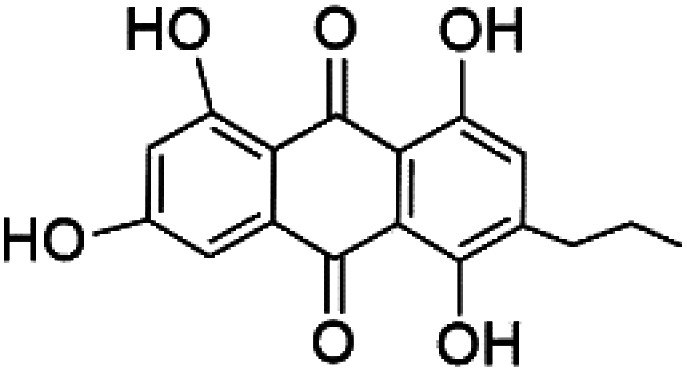			

## 6. Macrolides

Bastimolide A, a 40-membered ring polyhydroxy macrolide with 10 stereocentres and a rare *tert*-butyl terminus (130), was obtained from *Okeania hirsuta* marine *Cyanobacterium*. It displayed highly potent activity against four multidrug-resistant TM90-C2A, TM90-C2B, W2, and TM91-C235 (IC_50_ = 0.089, 0.11, 0.18, 0.34 nM, respectively) *P. falciparum* strains.^115^ Bastimolide B (131), polyhydroxy macrolide with a long aliphatic chain containing terminal *tert*-butyl group. It exhibited strong antimalarial activity against CQ-sensitive *P. falciparum* strain HB3 (IC_50_ = 5.7 μM).^116^ X-ray crystallography was used to determine the macrocyclic lactone's planar structure and absolute configuration, which consists of a 1,3-diol, one 1,3,5-triol, and six 1,5-diols. The rare *tert*-butyl group near the lactone ester in the bastimolide structure is thought to protect the lactone ring against hydrolysis.^117^

In addition, Palstimolide A, a polyhydroxy macrolide, was obtained from a tropical cyanobacterium Leptolyngbya sp. (Leptolyngbyaceae). Palstimolide A (132) had structural similarities to bastimolides. It displayed potent antimalarial activity against the blood stage of P. falciparum Dd2 strain (IC_50_ = 172.5 nM).^118^

Moreover, bromophycolides A (133), D (134), R (135), S (136), T (137), and U (138), diterpene benzoates macrolides were obtained from *Callophycus serratus* red algae (Onagraceae). Bromophycolides having 15- and 16-membered rings have been demonstrated to be effective antimalarials. No significant effects of the lactone ring size on activity were observed between 15- and 16-membered lactone rings. Some bromophycolides target haem crystallization, implying that haemozoin production is inhibited as a mechanism of action.^119^ Bromophycolide A presented good activity against drug-resistant Dd2 strain (IC_50_ = 377 nM), drug-sensitive 3D7, and HB3 strains (IC_50_ = 499 and 493 nM), respectively.^120^ The chemical structures of these compounds belonging to macrolides are traced in Table 6. Bromophycolides have been mentioned among drugs that are investigated in pre-clinical trials.^99^

**Table 6:** A list of marine-derived macrolides antimalarial drugs showing their IC_50_ against various strains of *Plasmodium* sp., chemical structure and biogenic source

Compound	Antiplasmodial activity (IC_50_ value)	Structure	Source	Marine class	Ref.
Bastimolide A (130)	TM90-C2A = 0.089TM90-C2B = 0.11 W2 = 0.18 TM91-C235 = 0.34 nM	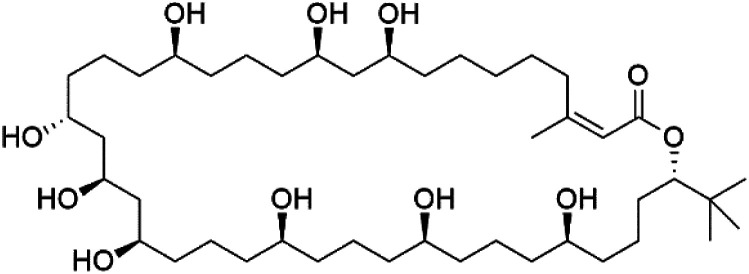	*Okeania hirsuta*	Cyanobacteria	115
Bastimolide B (131)	HB3 = 5.7 μM	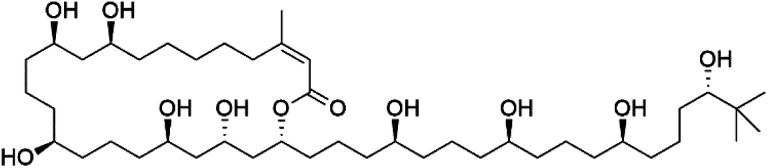			116
Palstimolide A (132)	Dd2 = 172.5 nM	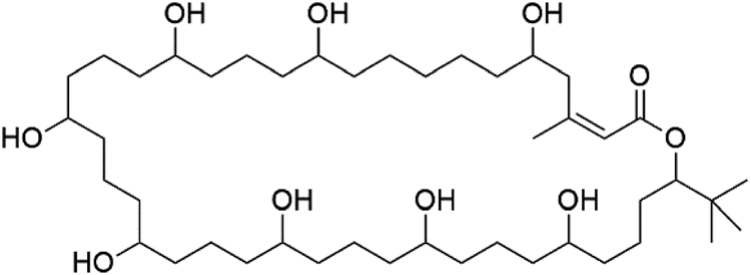	*Leptolyngbya* sp.	Cyanobacteria	118
Bromophycolide A (133)	Dd2 = 377 nM, 3D7 = 499 nM HB3 = 493 nM	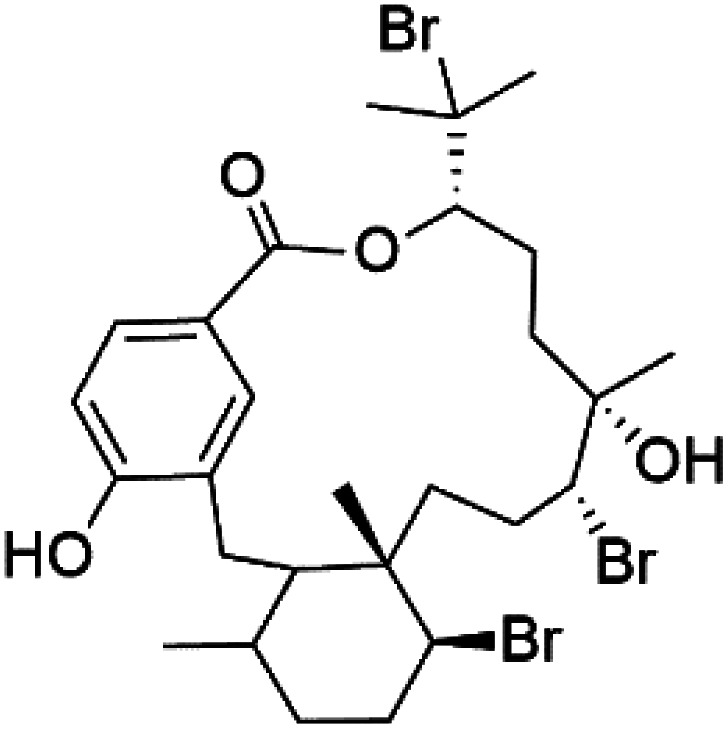	*Callophycus serratus*	Red alga	119
Bromophycolide D (134)		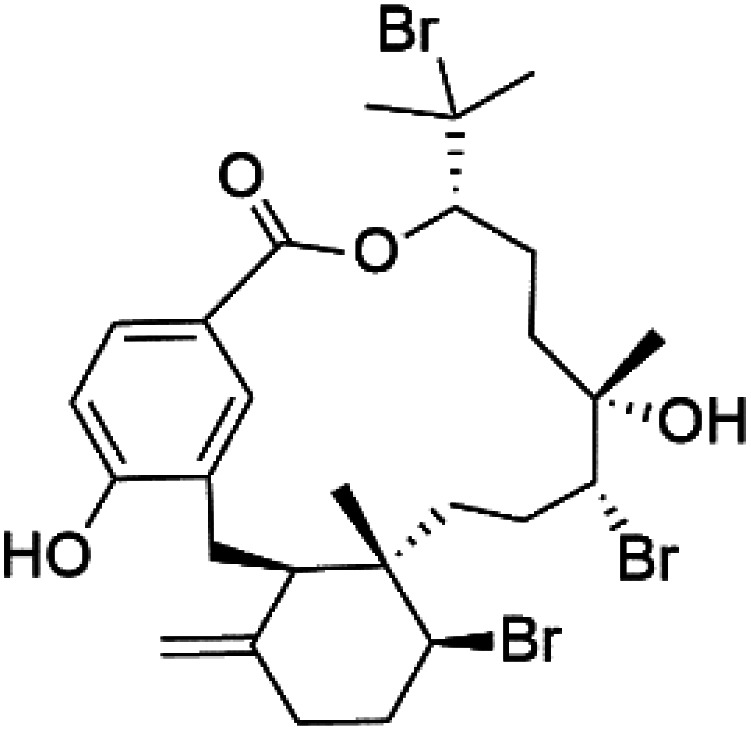			
Bromophycolide R (135)		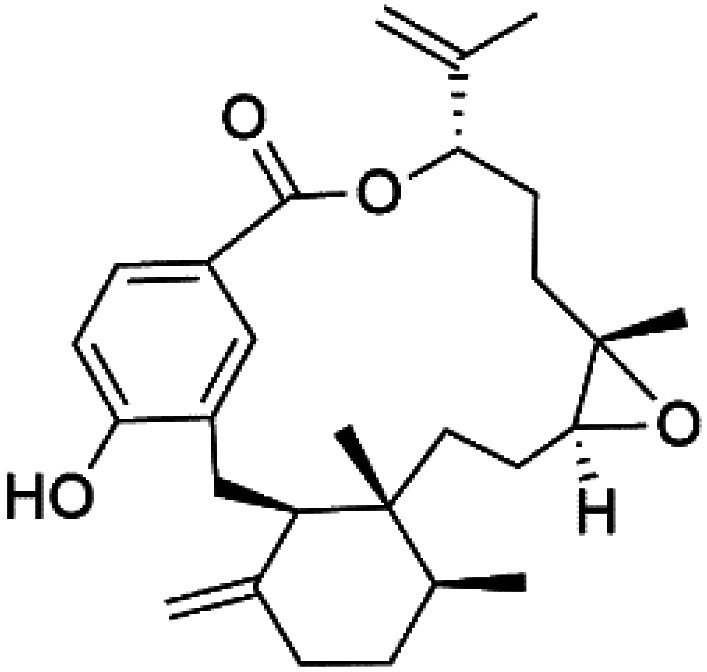			
Bromophycolide S (136)		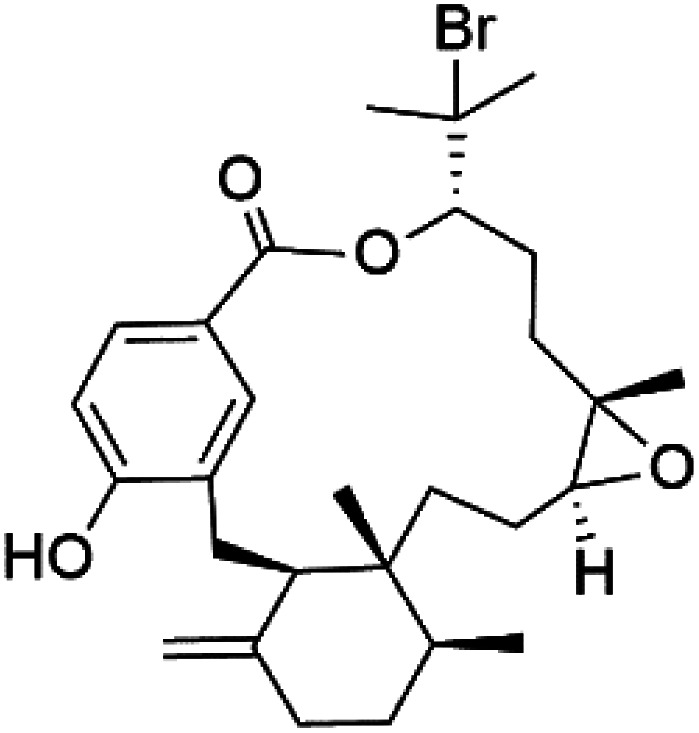			
Bromophycolide T (137)		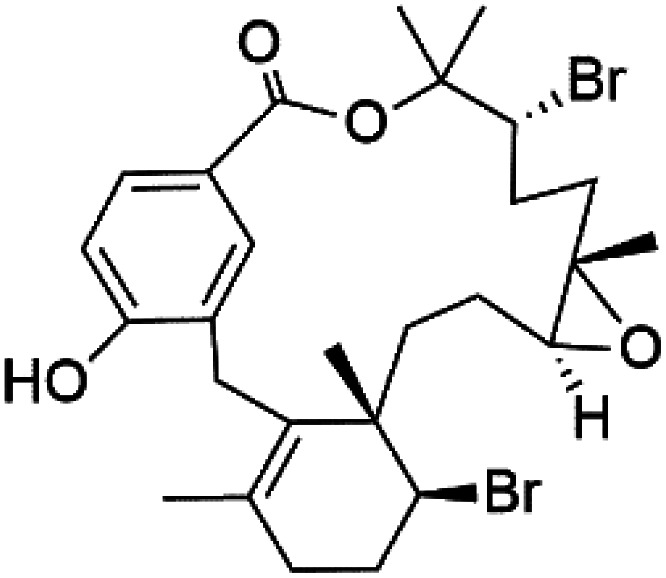			
Bromophycolide U (138)		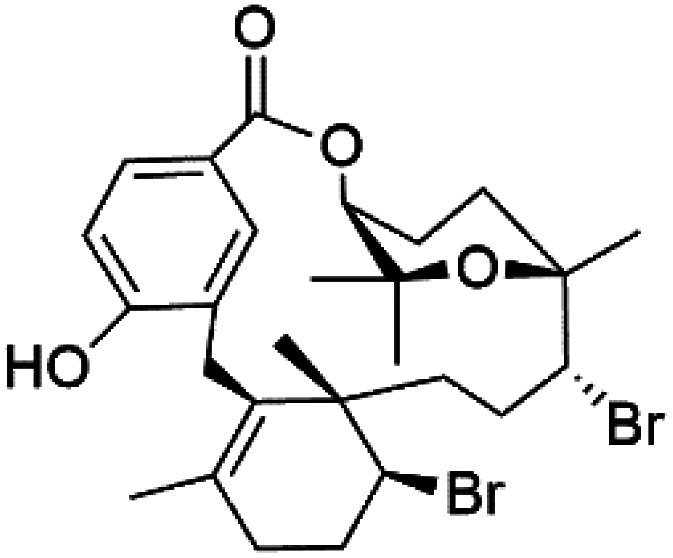			

## 7. Peptides and depsipeptides

Peptides are short cyclic or acyclic chains between two and fifty amino acids, linked by amide covalent bonds (peptidic bonds). At the same time, depsipeptides are cyclic or acyclic compounds of α-amino and α-hydroxycarboxylic acids linked to each other by esters and amides units.^121^ Several peptides and depsipeptides from marine sources were reported for antiplasmodial activity are, summarized in Table 7.

**Table 7:** A list of marine-derived peptides and depsipeptides antimalarial drugs showing their IC_50_ against various strains of *Plasmodium* sp., chemical structure and biogenic source^a^

Compound	Antiplasmodial activity (IC_50_ value)	Structure	Source	Marine class	Ref.
Balgacyclamide A (139)	K1 IC_50_ = of 9.0 and 8.2 μM, respectively	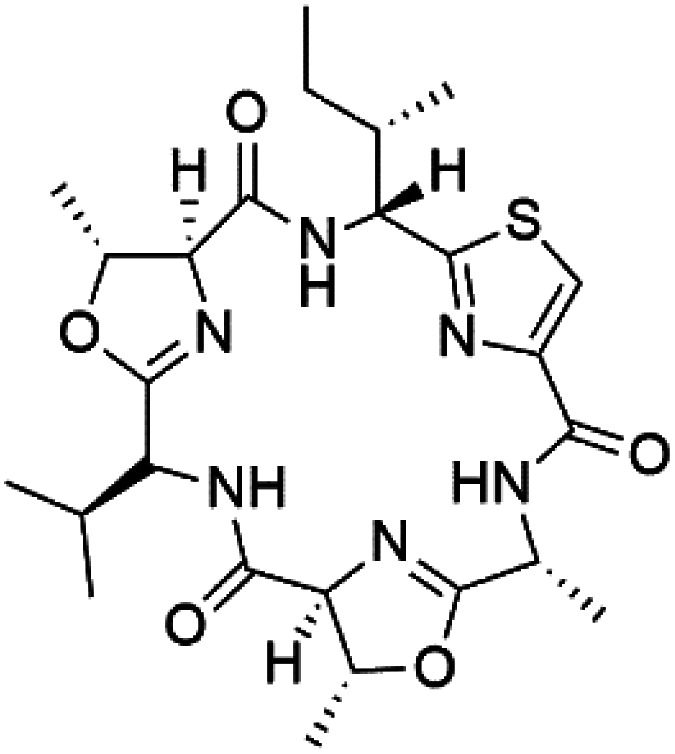	*Microcystis aeruginosa*	Cyanobacteria	122
Balgacyclamide B (140)		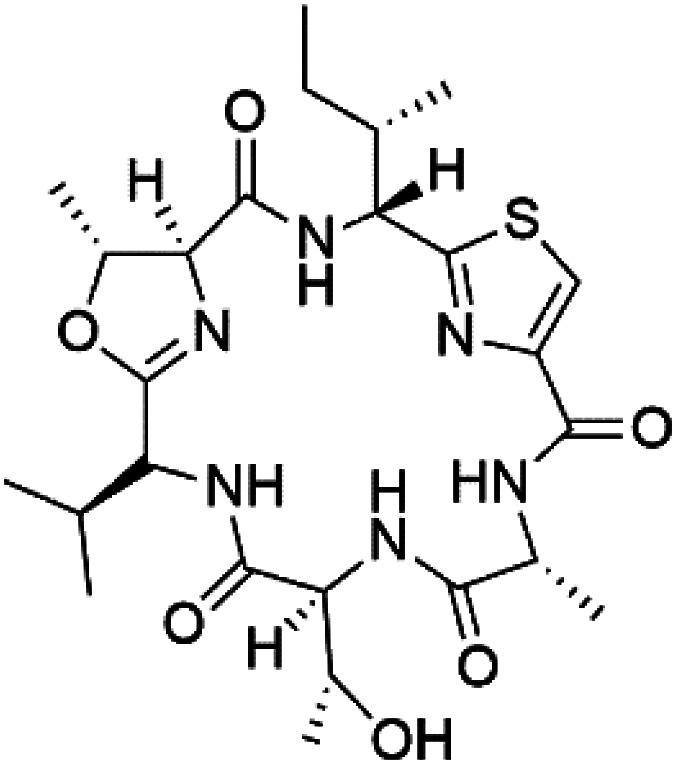			
Balgacyclamide C (141)	NA	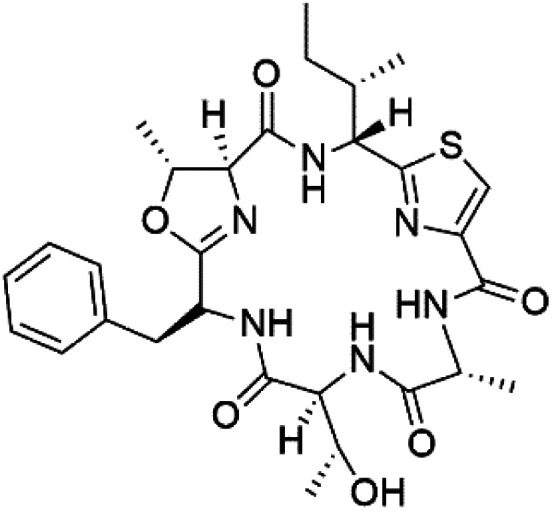			
Aerucyclamide A (142)	K1 = 5.0, 0.7, 2.3, and 6.3 μM, respectively	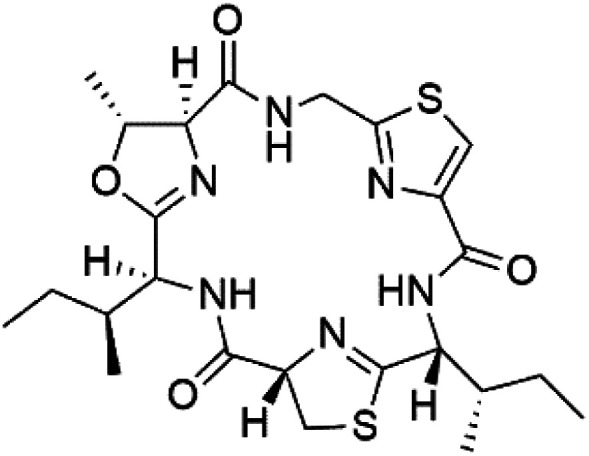	*Microcystis aeruginosa*	Cyanobacteria	123 and 124
Aerucyclamide B (143)		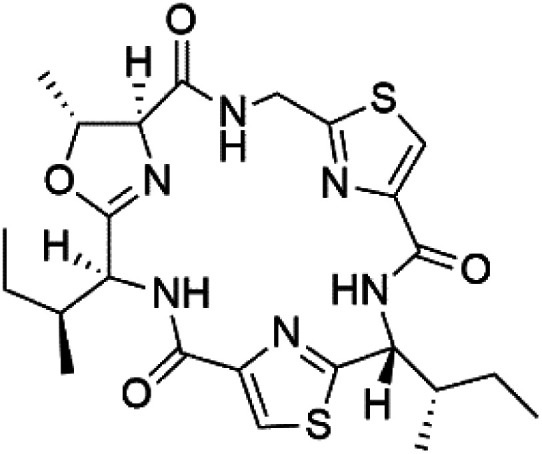			
Aerucyclamide C (144)		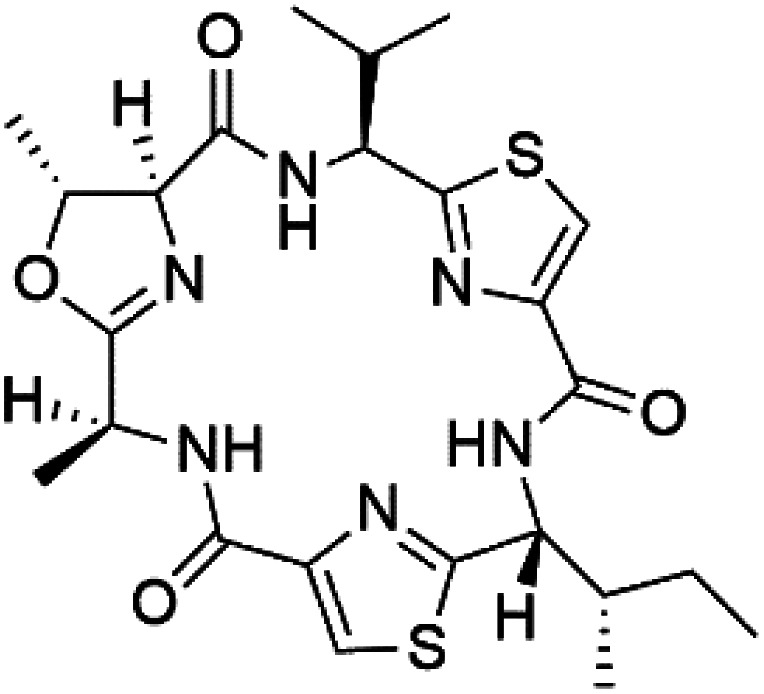			
Aerucyclamide D (145)		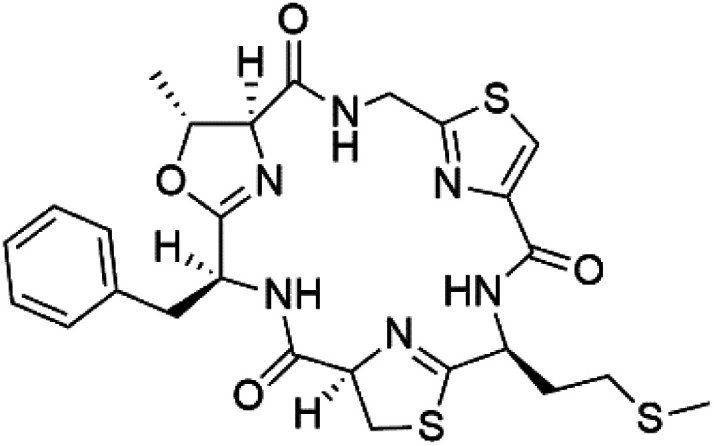			
Mollamide B (146)	D6 = 0.0029 μM W2 = 0.003 μM	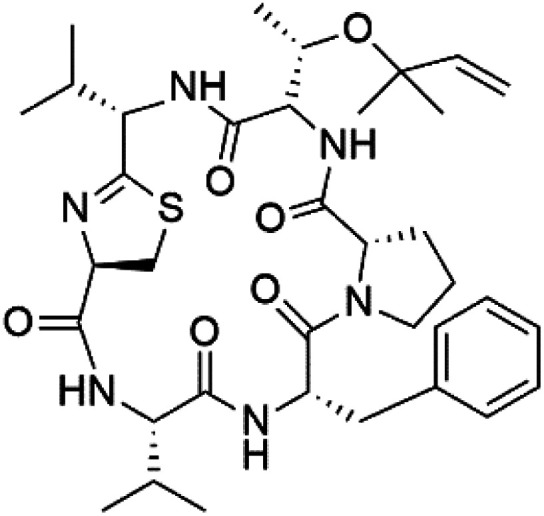	*Didemnum mole*	Tunicate	125
Dolastatin 10 (147)	FCH5·C2 = 0.1 nM	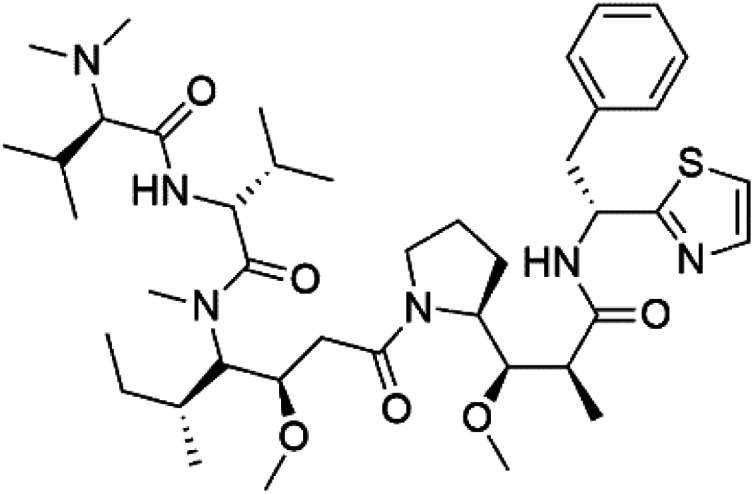	*Symploca* sp.	Cyanobacteria	126 and 127
Dragonamide A (148)	3D7 = 7.7, 7.0, and 6.0 μM	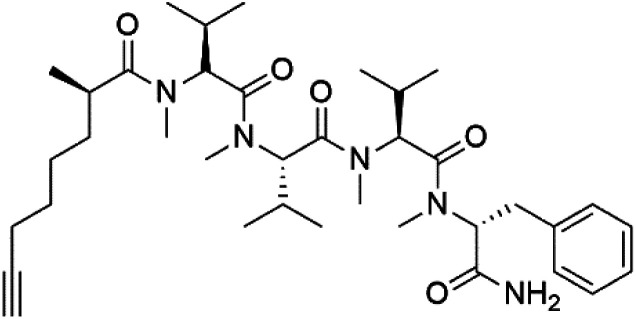	*Moorea producens*	Cyanobacteria	128 and 129
Dragonamide B (149)		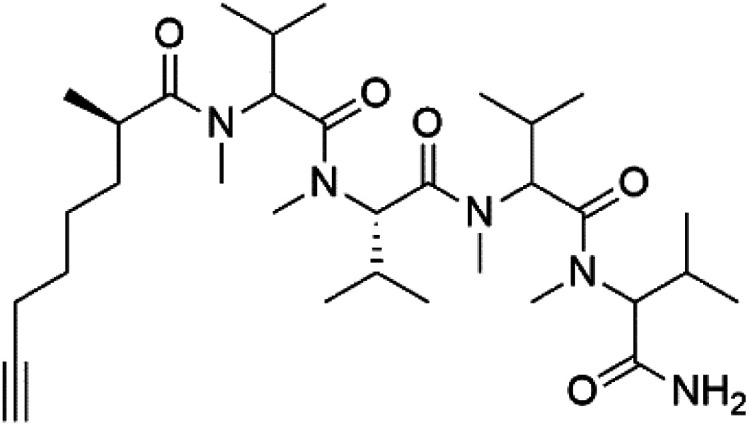			
Dragomabin (150)		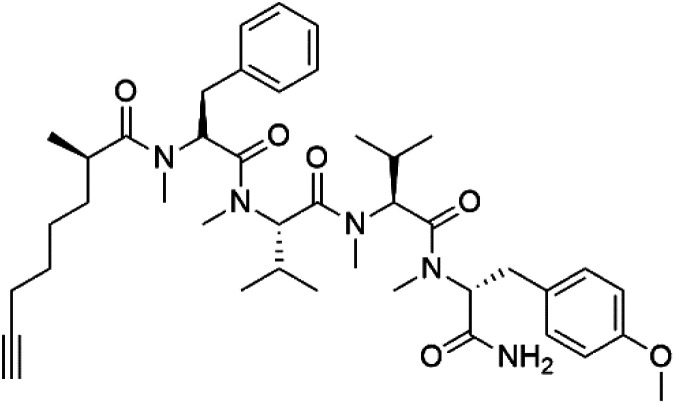			
Carmabin A (151)	3D7 = 4.3 μM	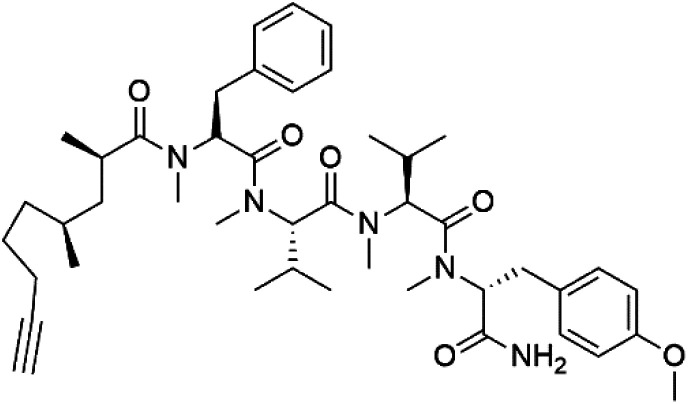			
Malyngamide X (152)	K1 ED_50_ = 5.44 μM	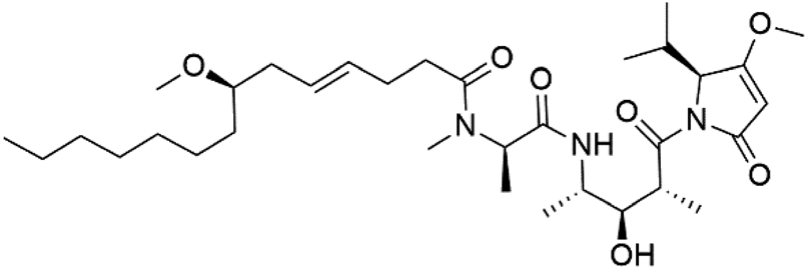	*Bursatella leachii*	Thai sea hare	130
Romidepsin (153)	∼150 nM	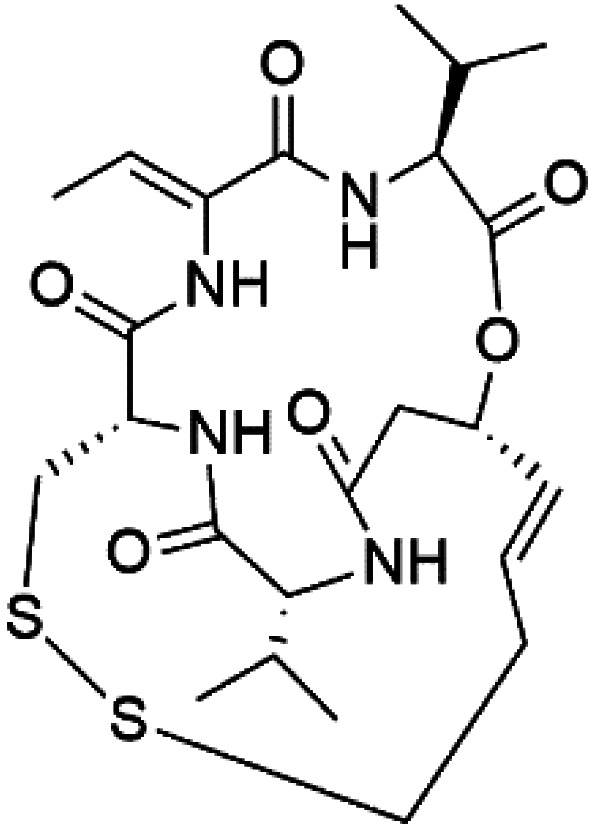	*Violaceous* sp	Cyanobacteria	131 and 132
Venturamide A (154)	W2 = 8.2 and 5.2 μM	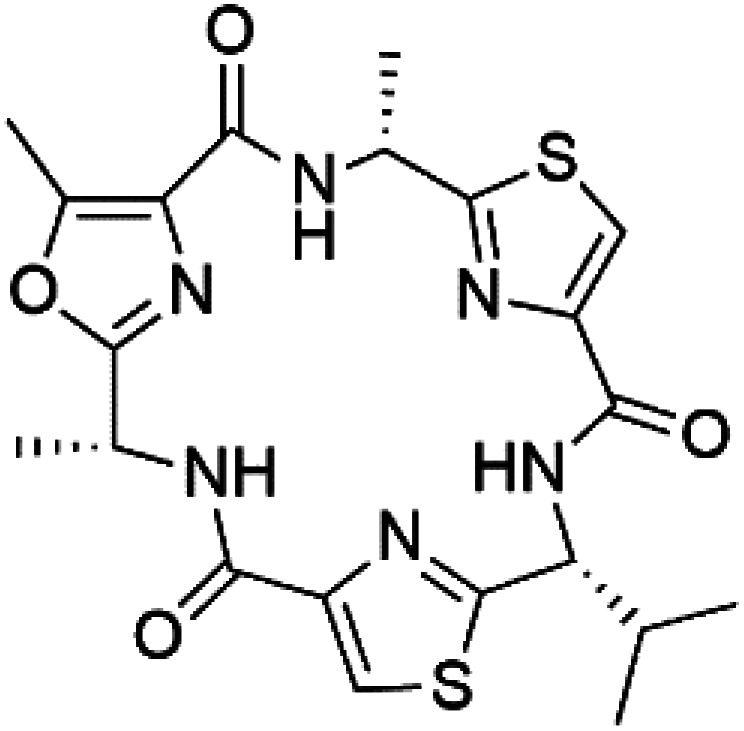	*Oscillatoria* sp.	Cyanobacteria	133
Venturamide B (155)		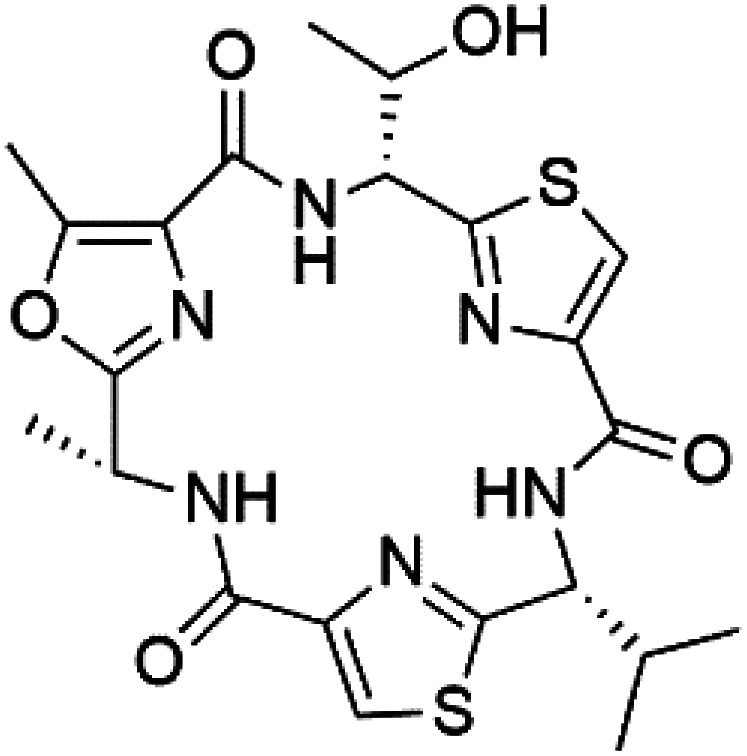			
Companeramide A (156)	D6 = 570 and 220 nM Dd2 = 1000 and 230 nM	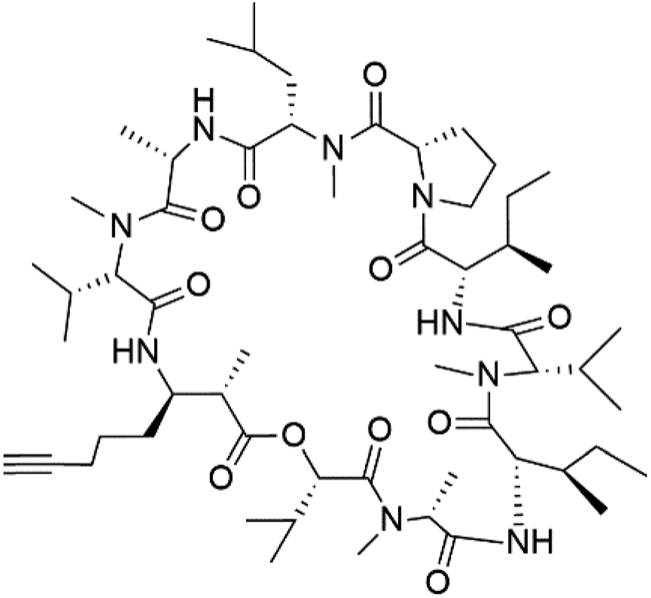	Panamanian marine *Cyanobacterium* sp.	Cyanobacteria	134
Companeramide B (157)		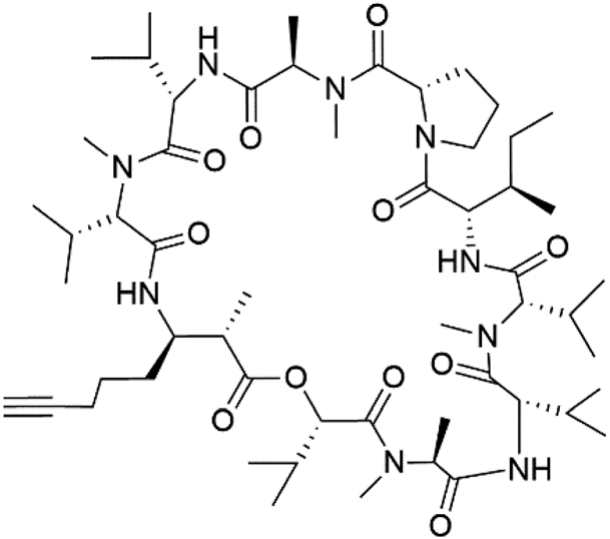			
Lagunamide A (158)	NF54 = 190, 910 and 290 nM	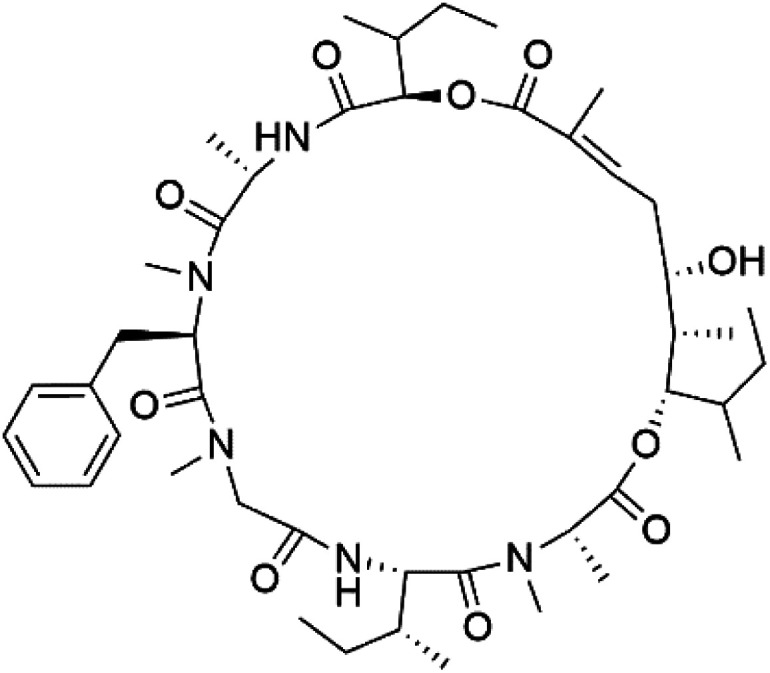	*Lyngbya majuscule*	Cyanobacteria	135 and 136
Lagunamide B (159)		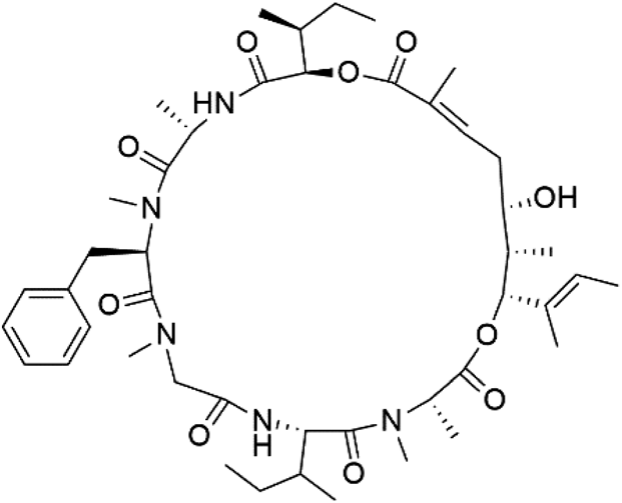			
Lagunamide C (160)		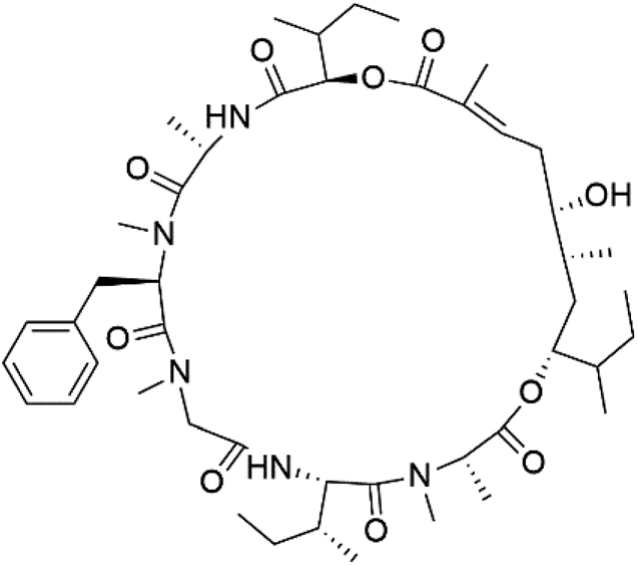			
Mollemycin A (161)	3D7 = 7 nM Dd2: 9 nM	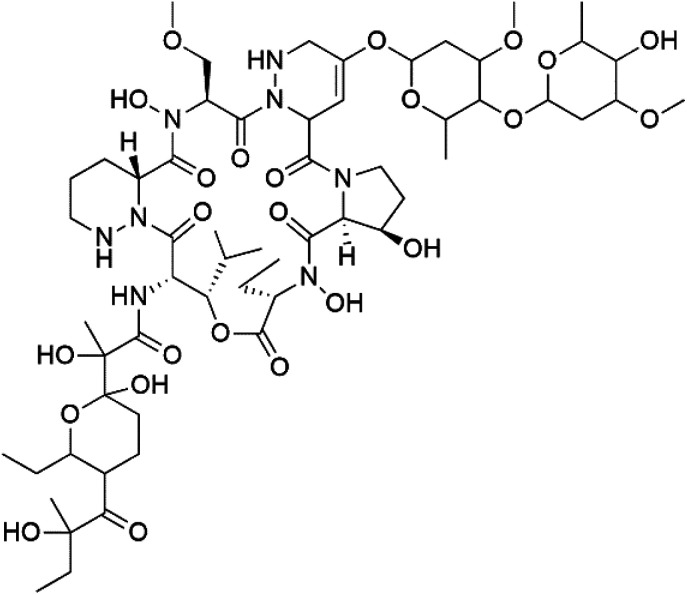	*Streptomyces* sp.	Marine actinomycetes	137
Symplocamide A (162)	W2 = 0.95 μM	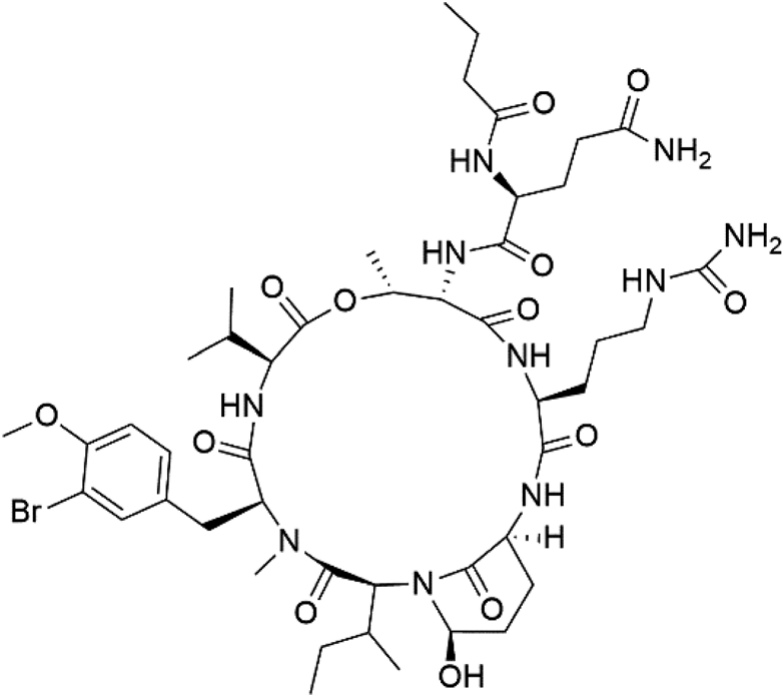	*Streptomyces ballenaensis* and *S. bangulaensis*	Marine actinomycetes	138 and 139
Actinoramide A (163)	HB3 = 190 nM Cp250 = 210 nM Dd2 = 220 nM 7G8 = 160 nM GB4 = 340 nM	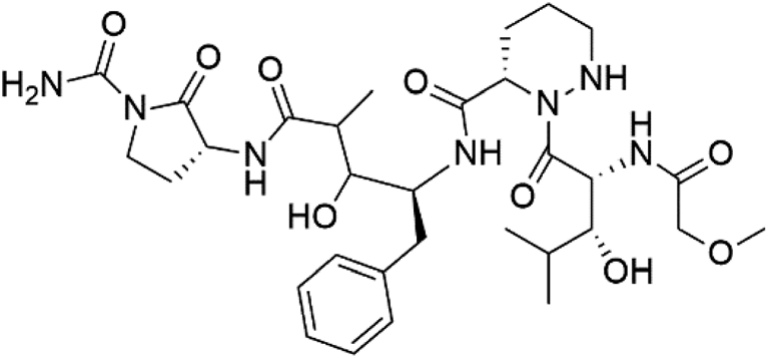			140
Symplostatin 4 (164)	3D7 ED_50_ = 74 nM	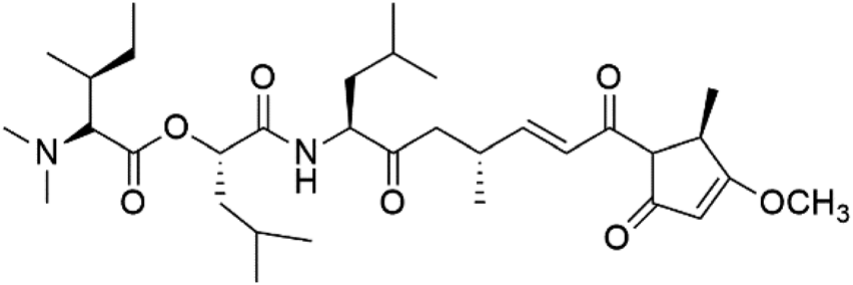	*Symploca* sp	Cyanobacteria	141 and 142
Gallinamide A (165)	3D7 = 50.1 ± 7.6 nM W2 = 8.4 μM	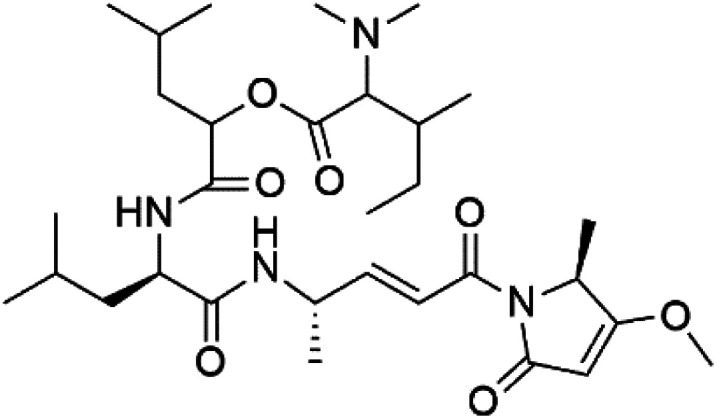	*Schizothrix* sp.	Cyanobacteria	144
Viridamide A (166)	W2 = 5.8 μM	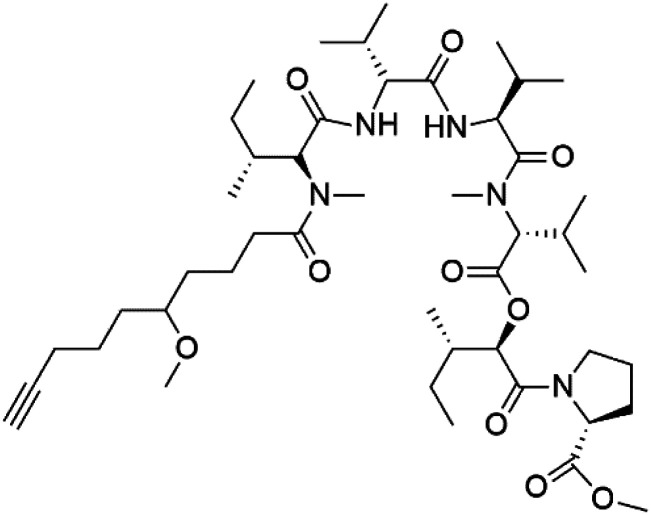	*Oscillatoria nigro-viridis*	Cyanobacteria	145

a NA: not available.

Even though a good number of peptides and depsipeptides have presented good antimalarial activity, their mechanism of action is not well understood. Some of them displayed a strong inhibitory effect on some key enzymes present in the malaria parasite; moreover, the relation between the inhibition and their antimalarial activity remains unestablished.^58^

### 7.1. Cyclic peptides

Three new heterocyclic macrocyclic peptides, Balgacyclamides A–C (139–141), were isolated from *Microcystis aeruginosa* (Microcystaceae). Balgacyclamides A and B displayed potent activity against the CQ-resistant strain K1 of *P. falciparum* (IC_50_ = of 9.0 and 8.2 μM), respectively.^122^ A class of modified hexacyclopeptides, aerucyclamides A–D (142–145) were obtained from the cyanobacterium *Microcystis aeruginosa*. They presented a potent activity against the K1 strain of *P. falciparum* (IC_50_ = 5.0, 0.7, 2.3, and 6.3 μM, respectively).^123,124^ Mollamide B (146), thiazoline hexapeptides found in the Indonesian tunicate *Didemnum mole* (Didemnidae). It showed a moderate antimalarial activity against D6 and W2 strains (IC_50_ = 0.0029 and 0.003 μM, respectively) of *P. falciparum*.^125^

### 7.2. Acyclic peptides

Dolastatin 10 (147), an acyclic peptide extracted from cyanobacterium *Symploca* species^126,127^ exhibited strong activity against *P. falciparum* 3D7 colonies, with IC_50_ = 74 nM. In addition, four acyclic lipopeptides, dragonamides A (148), B (149), dragomabin (150), and carmabin A (151) have been isolated from the cyanobacterium *Moorea producens* (Cyanobacteriaceae) (formerly *Lyngbya majuscula*). dragomabin, carmabin A and dragonamide A displayed good antimalarial activity (IC_50_ = 6.0, 4.3 and 7.7 μM, respectively).^128,129^

Morover, malyngamide X (152) is the first (7*R*)-lyngbic acid connected to a new tripeptide backbone. It was obtained from *Bursatella leachii* (Aplysiidae), a Thai sea hare, presented a moderate antimalarial activity with a half effective dose (ED_50_) = 5.44 μM against *P. falciparum* (K1) multidrug-resistant strain.^130^

### 7.3. Cyclic depsipeptides

Romidepsin (153), a cyclic depsipeptide histone deacetylase (HDAC) inhibitor, is responsible for the observed anti-Plasmodium activity of *Chromobacterium* species.^131,132^ Venturamides A (154) and B (155), two compounds from thiazole and oxazole cyclodepsipeptides class obtained from the cyanobacterium *Oscillatoria* sp., presented activity against (IC_50_ = 8.2 and 5.2 μM, respectively) *P. falciparum* W2 strain.^133^

Two cyclodepsipeptides, companeramides A (156) and B (157), were obtained from a Panamanian marine *Cyanobacterium* sp. (Cyanobacteriaceae). Exhibited strong antiplasmodial activity against D6 strain *in vitro* (IC_50_ = 0.57 and 0.22 μM, respectively).^134^ Three cytotoxic cyclic depsipeptides, lagunamides A–C (158–160), were isolated from *Lyngbya majuscule* (Oscillatoriaceae). The planar lagunamide macrocyclic scafold consists of peptide and polyketide substructures, and the main diferences are in the polyketide part. Lagunamides A and B are 26-membered macrocycles, while lagumanide C has an additional methylene carbon in the polyketide structure. Lagunamides A–C, showed potent activity against *P. falciparum* NF54 strain (IC_50_ = 0.19, 0.91, and 0.29 μM, respectively).^135,136^ The double bond in the side chain of lagunamide B might be responsible for the lower activity.^117^ Mollemycin A (161), a glycol-hexadepsipeptide-polyketide isolated from a marine-derived *Streptomyces* sp. CMBM0244 (Streptomycetaceae), exhibited a potent and selective growth inhibitory activity against drug-sensitive 3D7 and multidrug-resistant Dd2 clones of *P. falciparum* (IC_50_ = 7.0 and 9.0 nM, respectively).^137^ Symplocamide A (162), cyclodepsipeptide was extracted from the marine *Cyanobacterium symploca* sp. (Cyanobacteriaceae), showed potent antimalarial activity against W2 strain (IC_50_ = 0.95 μM) of *P. falciparum*.^138,139^ Cyclic tetrapeptide, actinoramide A (163) was isolated from marine actinomycetes *Streptomyces ballenaensis*, and *S. bangulaensis* (Streptomycetaceae). This tetrapeptide had potent activity against clones of drug-resistant *P. falciparum* including Cp250, Dd2, 7G8, and GB4 (IC_50_ = 210, 220, 160, and 340 nM, respectively) and drug-sensitive HB3 clone (IC_50_ = 190 nM).^140^

### 7.4. Acyclic depsipeptides

Symplostatin 4 (164), an acyclic depsipeptide extracted from *Symploca* sp. (Microcoleaceae). Symplostatin 4 showed a significant activity against 3D7 strain (ED_50_ = 74 nM) of *P. falciparum*.^141,142^ It displayed its activity on *P. falciparum* falcipains in infected red blood cells, indicating inhibition of the hemoglobin degradation pathway as a possible mode of action.^143^

A further cyanobacterial acyclic depsipeptide derivative, named gallinamide A (165) obtained from tropical reef *Schizothrix* sp. (Cyprinidae). Gallinamide A showed a moderate *in vitro* antimalarial activity against the W2 strain (IC_50_ = 8.4 μM) of *P. falciparum*.^144^ Viridamide (166), a lipodepsipeptide obtained from the cyanobacterium *Oscillatoria nigro-viridis* (Oscillatoriaceae), showed the activity against *P. falciparum* (IC_50_ = 5.8 μM).^145^

## 8. Phosphotriesters

A new class of antimalarials with long-chain bicyclic phosphotriesters, salinipostins A–K (167–177), Table 8, were obtained from *Salinospora* sp. bacteria (Micromonosporaceae). SAR fndings indicated that an increase in alkyl chain length attached to the phosphoester oxygen and vinyl carbon led to increased activity while branching of the alkyl causes a slight reduction in activity. The most active compound salinipostin A, did not afect parasite schizonts, indicating that it acts by disrupting the processes required for the establishment or growth of intracellular parasites. Salinispostin A did not inhibit haemozoin formation but cause cellular disorganization and disintegration of internal structure.^146^ These compounds showed different activity against *P. falciparum* W2 strain. Salinipostins A and D displayed the most potent activity (IC_50_ = 50 and 82 nM) followed by salinipostins I, B, F, C, G, E and H (IC_50_ = 0.126, 0.139, 0.266, 0.1415, 1.52, 3.22, 8.70 μM), respectively. Only, salinipostins K and J displayed weak activity.^146^

**Table 8:** A list of marine-derived phosphotriesters, polyether, and steroidal glycosides antimalarial drugs showing their IC_50_ against various strains of *Plasmodium* sp., chemical structure and biogenic source^a^

Compound	Antiplasmodial activity (IC_50_ value)	Structure	Source	Marine class	Ref.
Salinipostin A (167) Salinipostin F (172) Salinipostin I (175)	W2 = 50, 266, and 126 nM	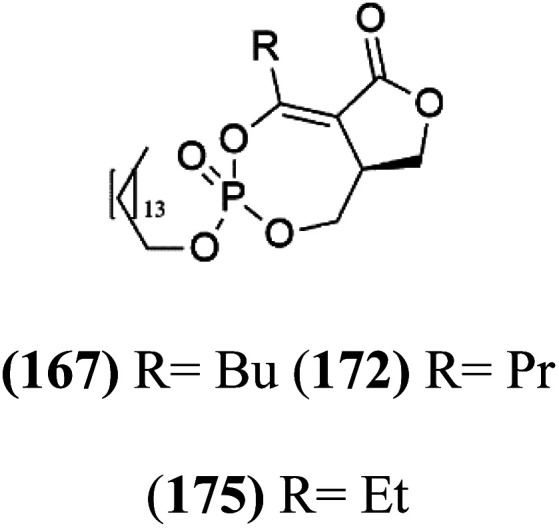	*Salinospora* sp.	Marine actinomycetes	146
Salinipostin B (168) Salinipostin D (170) Salinipostin G (173) Salinipostin J (176)	W2 = 139, 82, 1.52 μM, respectively (176) NA	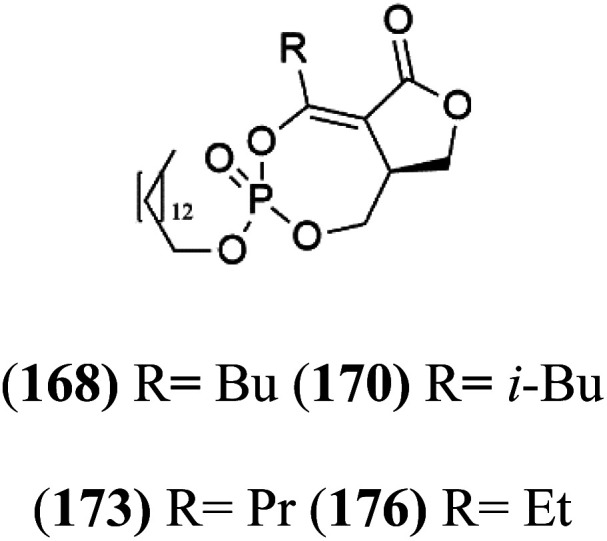			
Salinipostin C (169) Salinipostin E (171) Salinipostin H (174) Salinipostin K (177)	W2 = 415, 3.22, 8.70 μM, respectively (177) NA	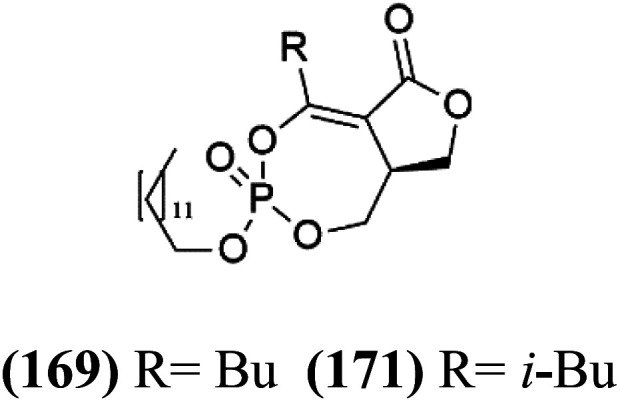			
Monensin (178)	D6 and W2 0.15 to 0.3 nM	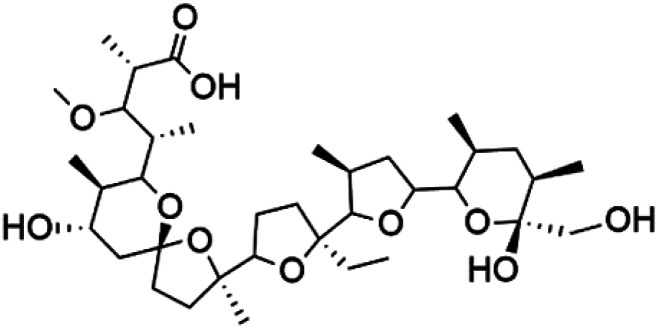	*Streptomyces cinnamonensis*	Marine actinomycetes	147 and 148
Pandaroside E (179)	W2 = 0.78.0.05 and 0.038 μM	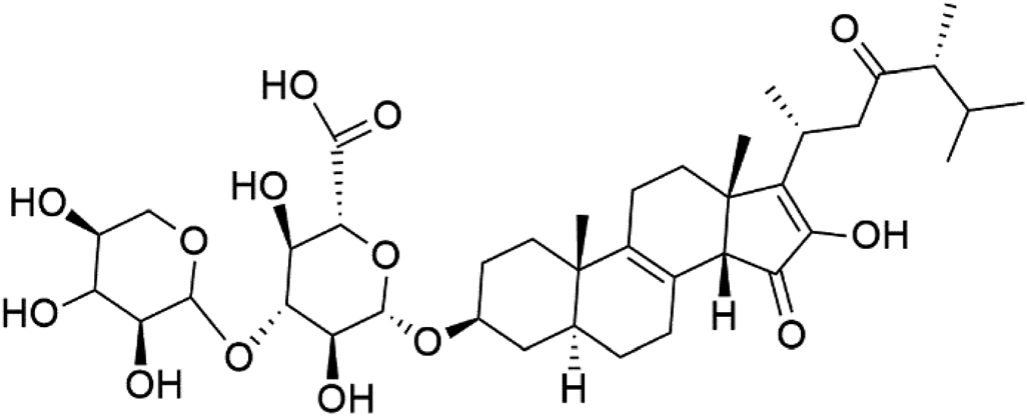	*Pandaros acanthifolium*	Sponge	150
Pandaroside G (180)		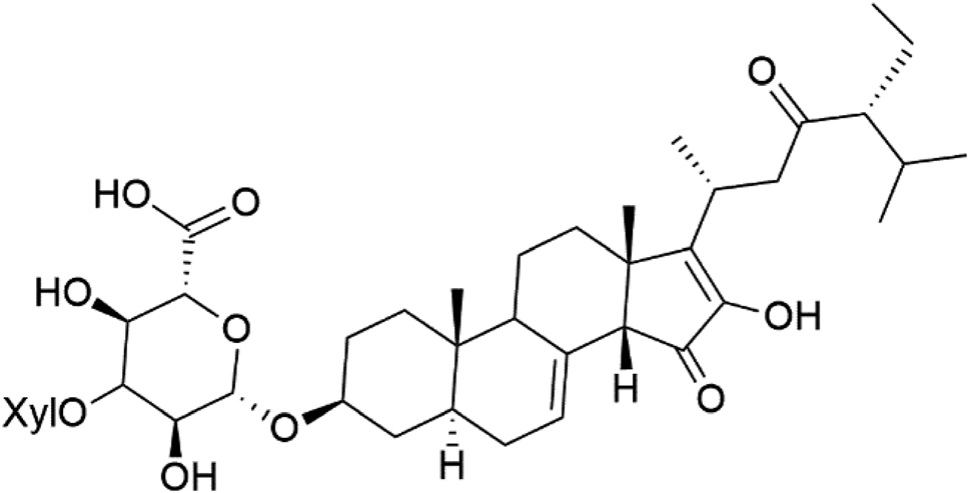			
Pandaroside H (181)		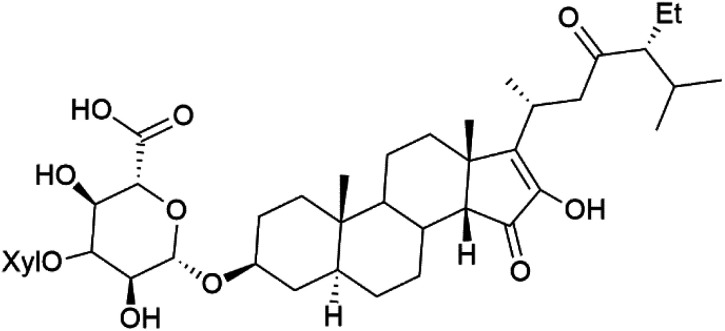			

a NA: not available.

## 9. Miscellaneous compounds

### 9.1. Polyethers

A polyether ionophore isolated from *Streptomyces cinnamonensis* (Streptomycetaceae) named Monensin (178), Table 8. Monensin has been displayed a strong antimalarial activity against *P. falciparum*.^147,148^ In a recent study, a polyether metabolite was extracted from Streptomyces sp. strain H668. This polyether displayed *in vitro* antimalarial activity against both D6 and W2 strains of P. falciparum with IC_50_ values from 0.15 to 0.3 nM.^149^

### 9.2. Steroid glycosides

The steroidal glycosides pandaroside E (179), G (180), and H (181) (Table 8), had been isolated from the Caribbean sponge *Pandaros acanthifolium* (Microcionidae). They strongly inhibited the growth of *P. falciparum* at low sub-micromolar concentrations.^150^

## 10. Conclusion

Malaria is among the crucial VBDs affecting the global health, based on the WHO official reports. It is easily progressed to impairment of important human body organs, including the liver, and death in case improper diagnosis and treatment. This emergency has acquired a special interest among health care providers and researchers to find more effective and safer medicaments, especially against the multi-resistant strain of *Plasmodium* sp. for the people of the developing countries. Particularly, natural-derived treatment of infectious diseases, including malaria is still the most convenient, safe, effective, and diverse. Marine organisms have attracted great potential in the last few decades as a promising and non-traditional source of bioactive compounds. Moreover, recent technological advances have led to isolate and identify thousands of marine-derived compounds belonging to various chemical classes. A total of 181 compounds derived from different marine sources, including sponges, cyanobacteria, marine algae, and actinomycetes, were reviewed in the current research possessing potential antimalarial activities with unique SAR and targeting different growth stages, including ring and trophozoite stage. More than half of the compounds belong to three major chemical classes comprising alkaloids, terpenoids, and polyketides. Such chemical diversity, potency, and less cytotoxicity are recognized as great start point for further SAR and clinical investigations of antimalarial candidates. The current article assumed that marine-derived natural products can also open up novel resources of bioactive compounds for novel candidates for management of other infectious diseases, exploring the oceans and seas treasures. Three compounds, including bromophycolides, plakortin, and homogentisic acid, are investigated as antimalarial drugs in pre-clinical trials and may be approved and marketed soon.

## Conflicts of interest

Authors declare that there are no known conflicts of interest associated with this publication and there has been no significant financial support for this work that could have influenced its outcome.

## List of abbreviations

CQ: Chloroquine
EC_50_: Half maximal effective concentration
ED_50_: Half maximal effective dose
FB: Iron-protoporphyrin IX
FDA: Food and Drug Administration
GSK-3P: Glycogen synthase 3
IC_50_: Half-maximal inhibitory concentration
MIC: minimum inhibitory concentration
SAR: Structure-activity relationships
VBDs: Vector-borne diseases
*P. falciparum* D6: A West African clone
*P. falciparum* W2: The Indochina clone W2
WHO: World Health Organization

## Supplementary Material
